# Cangfu Daotan decoction treats PCOS-IR through the IL6/JAK2/STAT3/FOXO4 signaling pathway

**DOI:** 10.3389/fendo.2025.1661000

**Published:** 2026-01-07

**Authors:** Wenhan Ju, Qianwen Zhang, Yue Wang, Keying Pan, Yuan Li, Shuai Zhao, Fang Lian

**Affiliations:** 1 Guanghua Hospital Affiliated to Shanghai University of Traditional Chinese Medicine, Shanghai, China; 2 Shandong University of Traditional Chinese Medicine, Jinan, China; 3 Affiliated Hospital of Shandong University of Traditional Chinese Medicine, Jinan, China

**Keywords:** polycystic ovary syndrome, insulin resistance, FOXO signaling pathway, stat3, cangfu daotan decoction

## Abstract

**Objective:**

This study aimed to investigate the protective effects and underlying mechanisms of Cangfu Daotan Decoction (CDD) in both *vivo* and *in vitro* models of polycystic ovary syndrome with insulin resistance (PCOS-IR).

**Materials and methods:**

Active compounds in CDD were identified using UPLC-HRMS. Network pharmacology and molecular docking analyses were employed to predict key molecular targets. A nd a high-fat diet. *In vitro*, KGN cells were used to simulate granulosa cell dysfunction associated with PCOS-IR. The regulatory effects of CDD on the IL6/JAK2/STAT3/FOXO4 signaling pathway were further evaluated.

**Results:**

Fifteen active compounds in CDD were preliminarily identified. Ninety-four potential target genes related to the treatment of PCOS-IR were screened. Network pharmacology and molecular docking analyses indicated strong binding affinities between STAT3 and several active compounds of CDD. CDD improved ovarian function and reduced insulin resistance in PCOS model mice. *In vitro*, CDD also enhanced glucose intake in granulosa cells under PCOS-IR conditions. Both *in vivo* and *in vitro* experiments demonstrated that CDD significantly suppressed activation of the IL6/JAK2/STAT3/FOXO4 signaling pathway.

**Conclusion:**

CDD may improve ovarian function and insulin resistance in PCOS-IR mice by modulating the IL6/JAK2/STAT3/FOXO4 signaling pathway.

## 1 Introduction

Polycystic ovary syndrome (PCOS), which affects approximately 15% of women worldwide (1), is a common reproductive endocrine disorder characterized by menstrual irregularities and infertility. The diagnosis of PCOS is a diagnosis of one of exclusion and requires the presence of at least two of the following three cardinal features: 1) clinical or biochemical hyperandrogenism; 2) ovulatory dysfunction; 3) polycystic ovarian morphology (2). Beyond reproductive abnormalities, PCOS can exert systemic effects, influencing multiple organ systems throughout the body (3). Clinical manifestations associated with PCOS include obesity, hirsutism, acne, hypertension, non-alcoholic fatty liver disease, and sleep-related breathing disorders. The etiology of PCOS remains incompletely understood; in combination with its complex and heterogeneous presentation, this complexity renders complete clinical cure currently unattainable.

Insulin resistance (IR) refers to reduced insulin efficiency in promoting glucose intake and utilization. Compensatory hyperinsulinemia occurs when the body secretes excessive insulin to maintain glucose homeostasis (4). The prevalence of IR is reported to be as high as 75% among patients with PCOS (5).Women with PCOS exhibit increased IR in adipose tissue, which is closely associated with systemic IR but not pancreatic β-cell function or adipocyte *GLUT4* gene expression (6). The development of IR adversely affects follicular maturation and ovulation. Elevated insulin levels activate insulin receptors in the pituitary gland, thereby promoting the secretion of luteinizing hormone (LH) (7). Hyperinsulinemia also decreases circulating sex hormone-binding globulin (SHBG) levels and increases free testosterone concentrations (8). In PCOS, hyperandrogenism, IR, and obesity interact to exacerbate the clinical manifestations. Insulin facilitates glucose transport into cells, where glucose catabolism generates adenosine triphosphate (ATP) to support cellular proliferation, differentiation, and other physiological processes. Granulosa cells provide metabolic energy to developing oocytes; therefore, locally produced ovarian insulin may play a role in regulating granulosa cell function and, consequently, oocyte quality. Human studies have demonstrated that gene and protein expression profiles in granulosa cells from patients with PCOS-IR differ significantly from those in healthy women (9, 10).

Traditional Chinese medicine (TCM) represents one of the most important therapeutic approaches for managing PCOS in China. For instance, a 2025 comprehensive review highlighted that dietary and herbal interventions may improve both metabolic and reproductive outcomes in PCOS patients (11). Specific natural compounds such as myo-inositol, resveratrol, berberine and quercetin exhibit multiple-target effects on hormonal regulation, insulin signalling and ovarian morphology in PCOS models (12). Pre-clinical studies of curcumin and its formulations likewise demonstrate promising results in ameliorating ovarian dysfunction and metabolic disturbance in PCOS models (13). In parallel, recent meta-analytical data published in 2024 on cinnamon supplementation revealed significant reductions in weight, HOMA-IR and LDL-cholesterol in women with PCOS, underscoring the translational potential of herbal interventions (14).

Network pharmacology provides a novel methodological framework for elucidating the pharmacological mechanisms of traditional medicines from a holistic and systems-level perspective, making it particularly suitable for studying the mechanisms of TCM formulas. Cangfu Daotan Decoction (CDD) was first documented in *Summary for Extending Offspring* in the Ming Dynasty in China, and it has been used to treat obesity-related female infertility. CDD is formulated according to the TCM principles of strengthening the spleen, resolving phlegm, tonifying the kidney, and regulating menstruation. CDD comprises *Atractylodes lancea* (Thunb.) DC. rhizome, *Cyperus rotundus* L. rhizome, *Poria cocos* (Schw.) Wolf, *Citrus reticulata* Blanco pericarp, *Pinellia ternata* (Thunb.) Breit. rhizome, *Arisaema heterophyllum* Blume rhizome, *Glycyrrhiza uralensis* Fisch. root and rhizome, *Zingiber officinale* Roscoe rhizome, immature *Citrus aurantium* L. fruit, and *Massa Fermentata* (Shenqu). Modern pharmacological studies have shown that CDD possesses significant anti-inflammatory activity, lowers blood glucose levels, and exerts therapeutic effects in PCOS (15–17). Our previous study revealed that CDD increases *PKP3* promoter methylation and decreases *PKP3* expression in patients with PCOS. In PCOS model rats and *in vitro* cultured granulosa cells, CDD was found to inhibit disease progression by disrupting the PKP3/ERCC1/MAPK signaling axis within ovarian granulosa cells (18). As an important link in the pathogenesis of PCOS, the effect of CDD intervention on local IR in ovarian is still unclear. Therefore, this study aimed to investigate protective effects and potential mechanisms of CDD in both *in vivo* and *in vitro* models of PCOS with PCOS-IR.

## 2 Materials and methods

### 2.1 Ethics statement

This study was conducted in compliance with the Declaration of Helsinki and in the Department of Reproduction and Genetics of the Affiliated Hospital of Shandong University of Traditional Chinese Medicine, which was approved by the Medical Ethics Committee within the healthcare organization (no. AF/SC-08/02.0) and registered in the China Clinical Trial Registry (no. ChiCTR2100053691). Informed consent documentation was provided by all subjects before sample collection. Animal experiments were ratified by the Animal Ethics Committee of Affiliated Hospital of Shandong University of Traditional Chinese Medicine (no. 2021021).

### 2.2 Animals and care

Sprague Dawley (SD) rats aged 6–8 weeks were purchased from Beijing Vital River Laboratory Animal Technology Co., Ltd. (license NO. SCXK (Beijing) 2019-0001). All rats weighed 200–230 g and were housed under controlled conditions (20-25 °C, 50-65% humidity, 12 h light/dark cycle) with free access to food and water. All procedures complied with the Animal Ethics Committee guidelines of the Affiliated Hospital of Shandong University of Traditional Chinese Medicine (approval no. 2021021).

### 2.3 Preparation of CDD-containing sera

CDD was based on 12g *Atractylodes lancea* (Thunb.) DC. rhizome (Batch No.18025832), 12g *Cyperus rotundus* L. rhizome(Batch No.18021402), 12g *Poria cocos* (Schw.) Wolf(Batch No.18025281, 18024511, and 18017581), 12g *Citrus reticulata* Blanco pericarp(Batch No.18016601), 9 g *Pinellia ternata* (Thunb.) Breit. rhizome(Batch No. 18025761), 9g *Arisaema heterophyllum* Blume rhizome(Batch No.18017471), 6g *Glycyrrhiza uralensis* Fisch. root and rhizome(Batch No.18024562), 6 g *Zingiber officinale* Roscoe rhizome(Batch No.18007141), 9g immature *Citrus aurantium* L. fruit(Batch No.18026232), and 9g *Massa Fermentata* (Shenqu) (Batch No.17021982).

All medicinal herbs were purchased by the Affiliated Hospital of Shandong University of Traditional Chinese Medicine and identified by Professor Lian Fang. Before preparing the drug containing serum, the medicinal materials were tested by our hospital’s traditional Chinese medicine Pharmacy to meet the requirements of the Pharmacopoeia of the People’s Republic of China (2022 edition) (19). *Atractylodes lancea* (Thunb.) DC. rhizome contains more than 0.2% atractylodin; *Cyperus rotundus* L. rhizome contains more than 0.8% volatile oil; *Poria cocos* (Schw.) Wolf contains more than 2.5% alcohol-soluble extract; *Citrus reticulata* Blanco pericarp contains more than 2.5% hesperidin; *Pinellia ternata* (Thunb.) Makino rhizome contains more than 7.5% water-soluble extract; *Arisaema heterophyllum* Blume rhizome contains more than 9.0% alcohol-soluble extract; *Glycyrrhiza uralensis* Fisch. root and rhizome contain more than 0.45% glycyrrhizin and more than 1.8% glycyrrhizic acid (C_42_H_62_O_16_); *Zingiber officinale* Roscoe rhizome contains more than 0.05% 6-gingerol; immature *Citrus aurantium* L. immature fruit contains more than 4.0% naringin and more than 3.0% neohesperidin; *Massa Medicata Fermentata* (Shenqu) contains more than 0.05% alcohol-soluble extract.

Twenty rats were randomly divided into CDD and normal control groups, with 10 rats in each group. Both groups were administered by gavage; the normal control group was administered saline, whereas the CDD group was administered CDD (6g/kg/d) (18). The drug was administered twice daily for 7 consecutive days. Blood samples were collected from the abdominal aorta one hour after the final administration. The serum was centrifuged, inactivated in a water bath for 30 min, filtered through a 0.22 μm microporous membrane to remove bacteria, and frozen at –80 °C.

### 2.4 Identification of CDD active ingredients by ultra performance liquid chromatography -high resolution mass spectrometry

The CDD extract, CDD-containing serum and blank control serum were extracted by acetonitrile vortex, cleaned-up by salting-out and PRiME HLB solid-phase extraction, and then chromatographed on a Waters ACQUITY UPLC HSS T3 column (100 mm×2.1 mm, 1.8 μm) with a gradient elution using aqueous ammonium acetate and acetonitrile at a flow rate of 0.4 mL/min at 35 °C, and an injection volume of 2 μL. The data were collected by an electrospray ionization (ESI) source in both the positive and negative ion modes. The data were analyzed by comparing them with reference standards using similarity evaluation, cluster analysis (CA), principal component analysis (PCA), orthogonal partial least squares methoddiscriminant analysis (OPSM), and the results were analyzed by the following methods.

### 2.5 Clinical follicular fluid collection

Ten patients diagnosed with PCOS combined with insulin resistance (PCOS-IR) who underwent *in vitro* fertilization (IVF) for assisted conception between January 1, 2022, and December 1, 2023, were enrolled in this study. The diagnosis of PCOS was established according to the Rotterdam Criteria (2): (1) oligo-ovulation or anovulation was a necessary criterion; (2) one of the following conditions was present: clinical manifestations of hyperandrogenism or hyperandrogenemia, ultrasound suggestive of polycystic ovaries (more than 12 follicles measuring 0.2–0.9 cm in at least one ovary or an ovary volume greater than or equal to 10 cm^3^); (3) except for other diseases that may trigger ovulatory disorders and hyperandrogenism. IR was defined using Homeostasis Model Assessment of Insulin Resistance (HOMA-IR) [fasting plasma glucose (FPG) (mmol/L) × fasting insulin (FINS) (µIU/mL)/22.5]. In previous clinical studies on PCOS conducted in China, a HOMA-IR value ≥ 2.29 was considered IR (20). Patients with any other endocrine disorders associated with PCOS (Cushing’s syndrome, congenital adrenocortical hyperplasia, hyperprolactinemia, and abnormal thyroid function) were excluded from this study. Patients with abnormal cardiovascular, cerebrovascular, hepatic, renal, and hematopoietic functions or during the acute infectious phase of genitourinary inflammation were excluded from the study.

Additionally, ten infertile women who underwent intracytoplasmic sperm injection (ICSI) due to severe oligozoospermia in their partners were recruited as controls.

All patients received a gonadotropin-releasing hormone (GnRH) agonist protocol. Those with oligomenorrhea were pretreated with Diane-35 before ovarian stimulation. A single intramuscular injection of 1.0-1.75 mg Triptorelin Acetate for Injection (3.75/cartridge, Ipsen Pharma Biotech) was administered for downregulation on day 21–23 of the menstrual cycle before IVF, with dosage adjusted according to body surface area. After 14 days, serum follicle-stimulating hormone (FSH), luteinizing hormone (LH), and estradiol (E_2_) levels were measured, together with endometrial thickness and follicle diameter, to assess pituitary suppression. Successful downregulation was defined as follicle diameter < 5 mm, endometrial thickness ≤ 5 mm, E_2_ < 30 pg/mL, FSH < 5 mIU/mL, LH < 5 mIU/mL. After successful downregulation, treatment was initiated with 225–300 IU of recombinant follicle-stimulating hormone β Injection (Puregon^®^,600 IU/count, N.V. Organon)/d and an appropriate amount of injectable urinary FSH (75 IU/count, Livzon Pharmaceutical Group Inc.) intramuscularly. Transvaginal ultrasonography and serum hormone assays were performed every 2–4 days, and the dose was adjusted according to follicular development up to the trigger day. When the leading follicle reached a diameter of 18 mm, ovulation was triggered by an intramuscular injection of 250 µg of recombinant human chorionic gonadotropin (Ovidrel™, 250 µg/vial) between 8:30 and 10:00 p.m. Oocyte retrieval was performed 34–36 hours later under transvaginal ultrasound guidance. Follicular fluid was collected and centrifuged at 3,000 rpm for 10 minutes for subsequent analysis.

### 2.6 Network pharmacology analysis and molecular docking validation

We obtained target information for 15 effective chemical components in CDD from the Traditional Chinese Medicine System Pharmacology Database (TCMSP; https://tcmsp-e.com/tcmsp.php) (21), ChEMBL (22, 23) (https://www.ebi.ac.uk/chembl/) and BindingDB (24) (www.bindingdb.org/) database. Disease gene sets for PCOS, IR were screened in Genecards (https://www.genecards.org/), OMIM (https://omim.org/), and DisGeNET (https://disgenet.com/) databases. After removing irrelevant chemical components, the gene set was imported into Cytoscape 3.10.1 to construct a chemical-target gene network map. The PPI network was obtained by importing the relevant target genes into the STRING database (https://string-db.org/), and mapping was completed using Cytoscape 3.10.1. The mol2 structure of the active ingredient was obtained as a ligand from the TCMSP database, and the protein structure was obtained as a receptor from the PDB database (25) (https://www1.rcsb.org/). Autodock Vina software was used to verify molecular docking (26, 27). The network diagram was imported into Cytoscape in tsv format for beautification and topological characterization, and the core 10 genes were screened by Cytoscape’s plugins Cytohubba plugins (28, 29).

### 2.7 Enrichment analysis

To further investigate the role of the differentially expressed genes in biological signaling pathways, the differentially expressed genes were imported into the DAVID 6.8 database (https://davidbioinformatics.nih.gov/) for gene ontology (GO) and Kyoto encyclopedia of genes and genomes (KEGG) pathway enrichment analyses (30, 31). GO enrichment analysis included biological process (BP), molecular function, and cellular component, and the species was set to be “Homo sapiens.” P < 0.05 was considered the threshold value. Protein-protein interaction (PPI) networks of differentially expressed genes were constructed by STRING (https://string-db.org/) online analysis tool (32).

### 2.8 Animal model establishment, grouping, and drug administration

All animal experiments were approved by the Experimental Animal Ethics Committee of Shandong University of Traditional Chinese Medicine (Approval No. 2021021). Ninety female C57BL/6 mice were randomly assigned to either a control group (n = 20) or a model group (n = 70). Letrozole (MCE, 112809-51-5) was dissolved in 0.5% sodium carboxymethyl cellulose (CMC-Na) (MCE, 9004-32-4) to prepare the gavage solution. Mice in the model group received a daily oral dose of 1 mg/kg letrozole combined with a high-fat diet for 21 consecutive days to induce the PCOS-IR model (33, 34). Mice in the control group were gavaged with an equal volume of 0.5% CMC-Na solution and fed a standard diet. The high-fat diet used for model induction contained 60% kcal from fat, 20% kcal from carbohydrate, and 20% kcal from protein (Research Diets, D12492, USA). The main source of fat was lard, and the carbohydrate source was maltodextrin and sucrose. The control diet contained 10% kcal from fat, 70% kcal from carbohydrate, and 20% kcal from protein.

During the final 10 days of letrozole administration, estrous cycles were monitored daily. After 21 days, three mice from each of the control and model groups were randomly selected, anesthetized with isoflurane, and blood samples were collected from the retro-orbital plexus. The mice were then euthanized via cervical dislocation, and ovarian tissues were sectioned and stained with hematoxylin–eosin (H&E) to confirm successful induction of the PCOS-IR model. Mice exhibiting disrupted estrous cycles and characteristic ovarian morphology were considered successfully modeled and selected for subsequent experiments.

A total of 45 successfully induced PCOS-IR mice were randomly assigned to three groups: PCOS-IR group (n = 15), low-dose CDD group (CDD-L, n = 15), and high-dose CDD group (CDD-H, n = 15). Additionally, 15 normal mice were selected as a control group (n = 15) for subsequent experiments. The CDD dosage for mice was calculated based on the human equivalent dose, using the body surface area conversion method (35). The conversion formula is: Animal dose (mg/kg) = Human dose (mg/kg) × conversion factor. Assuming a daily clinical dose of 96 g for a 70 kg adult, the mouse equivalent dose is approximately 12.48 g/kg, which corresponds to 9.1 times the human dose. The low and high doses used in this study were equivalent to 1× and 3× the clinical dose, respectively. Starting on day 21, mice in the CDD-L and CDD-H groups were gavaged daily with 12.48 g/(kg·d) and 37.44 g/(kg·d) of CDD, respectively. Mice in the control and PCOS-IR groups received an equivalent volume of normal saline. The treatment lasted for 14 days. During this period, body weight was recorded daily, and vaginal smears were performed to monitor the estrous cycle. After the final administration, mice were fasted for 12 hours with free access to water. Fasting blood glucose was measured via tail vein sampling. Subsequently, based on the body weight of each mouse, 20% glucose solution was administered by oral gavage at a dose of 2 g/kg. Blood glucose levels were then measured again at 15, 30, 60, 90, and 120 minutes. Finally, the mice were anesthetized with isoflurane, and blood samples were collected from the retro-orbital plexus. Finally, the mice were euthanized by cervical dislocation, and ovarian tissues were harvested for further analysis. The left ovary was fixed in 4% paraformaldehyde for histopathological evaluation, and the right ovary was snap-frozen in liquid nitrogen and stored at -80°C for subsequent Western blotting and RT-qPCR analyses.

### 2.9 Hormonal and biochemical analysis

Serum levels of luteinizing hormone (LH; BYabscience, BY-EM221468), anti-Müllerian hormone (AMH; BYabscience, BY-EM220514), follicle-stimulating hormone (FSH; BYabscience, BY-WJZF1604), estradiol (E_2_; BYabscience, BY-WJZF0048), SHBG(BYabscience, BYHS100917), testosterone (BYabscience, BY-WJZF0053) and insulin (BYabscience, FINSBY-EM220778) were measured using enzyme-linked immunosorbent assay (ELISA) kits according to the manufacturer’s instructions. Serum glucose levels were analyzed using a fully automated biochemical analyzer.

### 2.10 Ovarian tissue sectioning

Ovarian tissues were harvested and fixed in 4% paraformaldehyde for 24 hours. Following paraffin embedding, the samples were sectioned into 5 μm-thick slices and mounted onto glass slides. Standard histological processing was then performed, including deparaffinization, rehydration, hematoxylin and eosin (H&E) staining, dehydration, and mounting. The stained sections were examined and imaged using a NanoZoomer S60v2 digital slide scanner (Hamamatsu, Japan) for morphological evaluation.

### 2.11 KGN cell culture and transfection

The KGN cell line was originally isolated and characterized from a patient with invasive ovarian granulosa cell carcinoma in Japan. This cell line exhibits steroidogenic activity similar to that of normal human granulosa cells, including the ability to synthesize estradiol and progesterone, and has been widely used in studies of ovarian physiology and pathology (36). KGN cells were obtained from iCell Bioscience Inc. (Shanghai, China) and cultured in Dulbecco’s modified Eagle medium (DMEM)/F12 supplemented with 10% follicular fluid obtained from clinical patients, at 37 °C in a humidified incubator with 5% CO_2_. Cells were seeded into 6-well plates at a density of 3×10^5^ cells per well for subsequent experiments. When the cells reached 60% confluence, si-negative control (NC), si-*STAT3*, and si-*FOXO4* were transfected using Lipofectamine 3000 (Invitrogen). The KGN cells were harvested 48 h after transfection for subsequent analysis.siRNA was synthesized by Beijing Syngenbio Co., LTD. with the following sequence:si-NC:5’-UUCUCCGAACGUGUCACGUdTdT-3’, si-STAT3: 5’-ACGUUAUCCAGUUUUCUAGdTdT-3’, si-FOXO4:5’-ACAUAUCAAGAUCUAGAUCCUdTdT-3’.

### 2.12 Glucose intake

KGN cells were incubated in medium containing 17.5 mM glucose (C_0_) for 24 h. After incubation, the supernatant was collected, and the residual glucose concentration (C_1_) was determined using a glucose oxidase assay kit (Invitrogen, A22189). The cells from the same wells were lysed, and total protein concentration was measured using the BCA assay. Glucose uptake was calculated according to the formula (C_0_ − C_1_)/protein content and expressed as mmol/g protein.

### 2.13 CCK-8

The viability of KGN cells was determined using the Cell Counting Kit-8 (Beyotime, C0037). Cells were seeded in 96-well plates at a density of 1 × 10^4^ cells/well and cultured overnight to allow adherence. The following day, the medium was replaced with culture medium containing graded concentrations of follicular fluid (0%, 10%, 20%, 30%, 40%, and 50%) or CDD-containing serum (0%, 10%, 15%, 20%, 25%, and 30%). After 24 h of incubation, 10 μL of CCK-8 reagent and 100 μL of fresh basal medium were added to each well. The plates were incubated at 37 °C for 2 h, and the absorbance was measured at 450 nm using a microplate reader.

### 2.14 Fluorescence reporter gene experiment

KGN cells were seeded into 24-well plates at a density of 5×10^4^ cells per well and allowed to adhere overnight. Cells were then transiently transfected with a STAT3 promoter-driven fluorescent reporter plasmid using Lipofectamine 3000 (Invitrogen). After 6 h of transfection, the medium was replaced with fresh culture medium containing the indicated treatments, including CDD-containing serum (0%, 10%, 15%, 20%). Following 24 h of incubation, fluorescence intensity was measured using a microplate reader (excitation/emission: 485/535 nm). Relative promoter activity was calculated by normalizing the fluorescent signal to total protein content or co-transfected Renilla luciferase. Each experiment was performed in triplicate, and results were expressed as the mean ± standard deviation.

### 2.15 Quantitative real-time polymerase chain reaction

The sequences of the *IL6*, *JAK2*, *STAT3*, *FOXO4*, and *GAPDH* primers are presented in **Table 1**. Total RNA was isolated from KGN cells using TRIzol^®^ Reagent kit (item #15589226, USA, Invitrogen) and subjected to reverse transcription using the ReverTra Ace^®^ RT-qPCR kit (Toyobo). Quantitative real-time PCR was conducted using SYBR Green qPCR Master Mix following the manufacturer’s instructions. The relative expression of all mRNAs was normalized to that of GAPDH. All experiments were repeated three times, and the relative gene expression was calculated using the 2^–ΔΔCt^ method.

**Table 1:** Primers used for quantitative real-time polymerase chain reaction.

Gene	Primer	Primer sequence
*STAT3 (Human)*	Primer F	GGAGAAGGACATCAGCGGTAAGA
	Primer R	CCTCCTTGGGAATGTCAGGATAG
*FOXO4 (Human)*	Primer F	ACCTGGAGTGTGACATGGATAAC
	Primer R	GAGGGCTCAAGGGTAAAGAGTAG
*IL6 (Human)*	Primer F	AACATGTGTGAAAGCAGCAAAGA
	Primer R	CTCTGGCTTGTTCCTCACTACTC
*JAK2 (Human)*	Primer F	GGTGGAGAACGAGAACAGAGTTA
	Primer R	CACTCCAAAGCTCCAAACATCTG
*GAPDH (Human)*	Primer F	CCTTCCGTGTCCCCACT
	Primer R	GCCTGCTTCACCACCTTC
*Stat3 (Mouse)*	Primer F	AGAGCTGGCTGACTGGAAGAGG
	Primer R	TTGTTGGCGGGTCTGAAGTTGAG
*Foxo4 (Mouse)*	Primer F	CCAGCCATGACAGAATGCCTCAG
	Primer R	TGAAGTCCAGTCCCTCACCATCC
*Il6 (Mouse)*	Primer F	CTTCTTGGGACTGATGCTGGTGAC
	Primer R	AGGTCTGTTGGGAGTGGTATCCTC
*Jak2 (Mouse)*	Primer F	GTGTGGAGATGTGCCGCTATGAC
	Primer R	AGTCTCGGAGGTGCTCTTCAGTG
*Gapdh (Mouse)*	Primer F	TGGTGAAGCAGGCATCTGAG
	Primer R	GTTGCTGTTGAAGTCGCAGG

### 2.16 Western blot

Total protein was extracted from cells using radioimmunoprecipitation assay (RIPA) buffer. Protein concentrations were determined using the Rapid Gold BCA Protein Quantification Kit (Boster, China). After quantification, equal amounts of protein (50 µg per sample) were separated by sodium dodecyl sulfate-polyacrylamide gel electrophoresis (SDS-PAGE) and transferred onto polyvinylidene fluoride (PVDF) membranes. After transfer, the membranes were briefly rinsed with TBST and then blocked with 5% skim milk powder in TBST for 2 h at room temperature. The membranes were then incubated overnight at 4 °C with primary antibodies against IL6 (1:1000, 66146-1-AP, Proteintech), IL6 (1: 500, GB11117, Servicebio), JAK2 (1:5000, EPR108(2), Abcam), STAT3 (1:2000, 10253-2-AP, Proteintech), p-STAT3 (1:1000, 80199-2-RR, Proteintech), FOXO4 (1:1000, 21535-1-AP, Proteintech), β-actin (1:20000, 66009-1-Ig, Proteintech) and GAPDH (1:5000, 60004-1-Ig, Proteintech). The PVDF membrane was washed thrice with TBST and then immersed in a secondary antibody (1:5000, Goat Anti-Rabbit, AS014, ABclonal) incubation solution and incubated for 1 h at room temperature. Finally, the proteins were visualized with an electrochemiluminescence solution and analyzed using Image J software. Quantitative bar graphs represent mean ± SD of three independent experiments analyzed by ImageJ. Relative protein expression was normalized to β-actin or GAPDH.

### 2.17 Data analysis

All experimental data are expressed as the mean ± standard deviation. To determine significance, the data were analyzed using unpaired Student’s *t*-tests or one-way analysis of variance followed by Tukey’s multiple comparison test using the GraphPad Prism software. Conversely, data with a non-normal distribution are expressed as the median (25^th^–75^th^ percentile), and the Mann–Whitney test was utilized for within-group comparisons. P< 0.05 was considered statistically significant.

## 3 Results

### 3.1 Identification of CDD active ingredients

**Figure 1:**
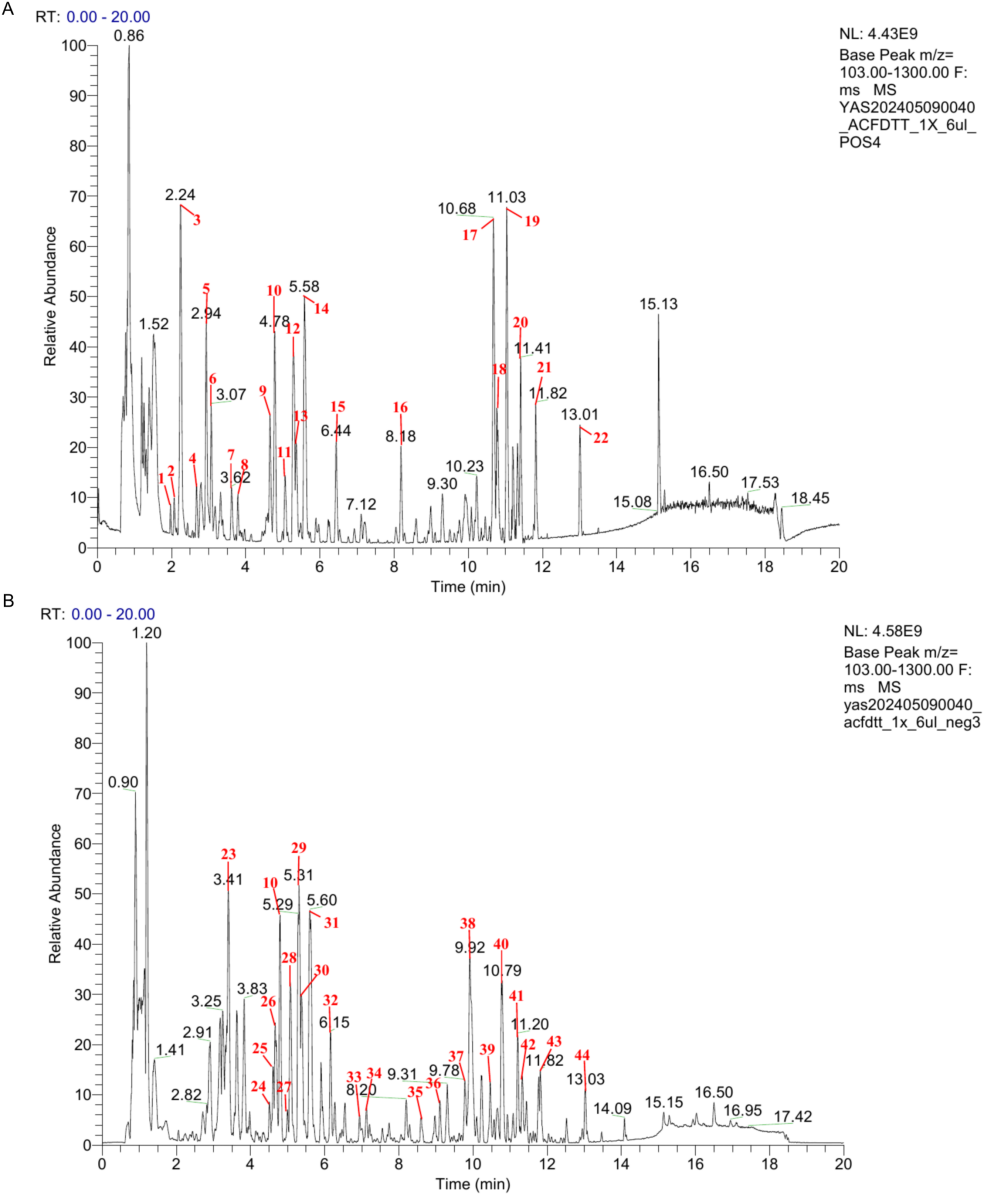
Base peak chromatogram of CDD. **(A)** The negative mode of the base peak chromatogram; **(B)** The positive mode of the base peak chromatogram.Time and peak are indicated by the numbers given for each peak. Specific ingredients are shown in **Table 2**. These compounds represent all identifiable major components based on LC-MS analysis.

UPLC-HRMS was used to analyze the active compounds of CDD. Qualitative and quantitative comparisons among CDD extract, blank control serum, and CDD-containing serum revealed a total of 1834 compounds as containing 153 blood-absorbed compounds in positive ion mode (POS), and 1067 compounds were detected as containing 119 blood-entry compounds in negative ion mode (NEG). In the positive and negative ion base peak chromatogram (BPC) of CDD, the peaks with higher abundance were confirmed by peak shape and examined by secondary spectra, respectively, and then the positive and negative ion graphs were labeled with chromatographic peaks in numerical order, as shown in **Figures 1A, B**. Fifteen of the 44 labeled peaks corresponded to blood-absorbed compounds, indicating that these may represent the main bioactive components of CDD (**Table 2** for details).

**Table 2:** Identified active ingredients.

No	RT(min)	ESI	MS	ppm	Score	Compound	Molecular formula	PubChem CID	CAS	SuperClass	Into blood or not
1	1.97	[M+H]+	283.1406	12.5	0.7657	p-Malonotoluidide	C17H18N2O2	95277	5469-94-3	NA	None
2	2.07	[M+H]+	115.0394	4	0.9968	Methyl tetronate	C5H6O3	643477	69556-70-3	Cyclic polyketides	None
3	2.24	[M+H- C6H10O]+	166.0864	0.6	0.9995	N-(4-Methylpentanoyl)phenylalanine	C15H21NO3	43183355	67036-32-2	Small peptides	None
4	2.7	[M+H]+	262.1916	2	0.986	4-(benzenesulfonyl)-N-propylpiperazine-1-carboxamide	C14H20N2O3S	4090947	609360-49-8	Lysine alkaloids	None
5	2.96	[M+H]+	205.0971	0.7	0.9986	L-Tryptophan	C11H12N2O2	6305	73-22-3	Small peptides	None
6	3.11	[M+H]+	265.1548	0.9	0.9438	Triamcinolone diacetate	C25H31FO8	10384	67-78-7	Ornithine alkaloids	None
7	3.65	[M+H-C6H6O3]+	177.0546	1.3	0.9905	Homoeriodictyol	C16H14O6	73635	446-71-9	Flavonoids	None
8	3.81	[M+H]+	595.1669	2.8	0.9954	Vicenin-2	C27H30O15	442664	23666-13-9	Flavonoids	Into Blood
9	4.66	[M+H-C6H10O5]+	257.0806	1	0.9759	Isoliquiritin	C21H22O9	5318591	5041-81-6	Flavonoids	Into Blood
10	4.8	[M+H-C6H10O5]+	257.0806	1	0.9778	Liquiritin	C21H22O9	503737	551-15-5	Flavonoids	None
11	5.08	[M+H]+	273.0754	1.5	0.9944	Naringenin chalcone	C15H12O5	5280960	73692-50-9	Flavonoids	None
12	5.3	[M+H-C12H20O9]+	273.0755	0.5	0.995	Compound NP-008751	C28H32O16	44715618	NA	Flavonoids	None
13	5.37	[M+H]+	611.1973	0.7	0.9721	MCULE-9952557572	C20H30O4	57509459	NA	Flavonoids	None
14	5.61	[M+H]+	303.0863	1.3	0.989	Hesperetin	C16H14O6	72281	520-33-2	Flavonoids	None
15	6.45	[M+H]+	261.1122	3.1	0.9994	Isomeranzin	C15H16O4	473252	1088-17-1	Coumarins	Into Blood
16	8.2	[M+Na]+	747.211	3.1	0.9854	Melitidin	C33H40O18	168011951	NA	Flavonoids	None
17	10.69	[M+H]+	403.1391	2.2	0.9982	Nobiletin	C21H22O8	72344	478-01-3	Flavonoids	Into Blood
18	10.78	[M+H- H2O]+	453.3368	1.5	0.9904	18β-glycyrrhetinic acid	C30H46O4	10114	471-53-4	Triterpenoids	Into Blood
19	11.04	[M+H]+	433.1497	2.1	0.9397	Hibiscetin heptamethyl ether	C22H24O9	5318050	21634-52-6	Flavonoids	Into Blood
20	11.42	[M+H]+	373.1286	0.6	0.995	Tangeretin	C20H20O7	68077	481-53-8	Flavonoids	None
21	11.83	[M+H- 2H2O]+	357.2791	1	0.9618	Isohyodeoxycholic acid	C24H40O4	5283822	570-84-3	Steroids	None
22	13.02	[M+H]+	219.1745	1.1	0.9959	alpha-Cyperone	C15H22O	6452086	473-08-5	Sesquiterpenoids	None
23	3.41	[M-H]-	165.0546	6.2	0.9783	Hydrocinnamic acid	C9H10O2	107	616-34-2	NA	Into Blood
24	4.51	[M-H]-	595.166	1.8	0.9942	Eriocitrin	C27H32O15	83489	13463-28-0	Flavonoids	None
25	4.61	[M-H]-	649.2494	1.5	0.982	yl(hexopyranosyloxy)m(4aS,8aR,9R,10S,12bR)-10-((S)-Furan-3-ethyl)-6,6,8a,10-tetramethyl-3,8-dioxodecahydro-3H,6H-spiro[naphtho[1’,2’:3,4]furo[3,2-c]pyran-9,2’-oxirane]-3’-carboxylic acid	NA	NA	NA	NA	None
26	4.66	[M-H]-	549.1605	1.5	0.9822	Liguiritigenin-7-O-beta-D-apiosyl-4’-O-beta-D-glucoside	C26H30O13	124578359	199796-12-8	Flavonoids	None
10	4.8	[M-H]-	417.1184	1	0.9859	Liquiritin	C21H22O9	503737	551-15-5	Flavonoids	Into Blood
27	4.99	[M-H]-	165.0545	2.7	0.9887	3-(3-hydroxyphenyl)propanoic acid	C9H10O3	873	495-78-3	NA	None
28	5.08	[M-H]-	579.1709	1.4	0.9947	Narirutin	C27H32O14	442431	14259-46-2	Flavonoids	Into Blood
29	5.3	[M-H]-	579.1709	1.7	0.9769	Naringin	C27H32O14	442428	10236-47-2	Flavonoids	None
30	5.38	[M-H]-	609.1818	1	0.996	Hesperidin	C28H34O15	10621	20196-67-2	Flavonoids	Into Blood
31	5.6	[M-H]-	609.1816	1.1	0.9694	Neohesperidin	C28H34O15	442439	13241-33-3	Flavonoids	Into Blood
32	6.16	[M-H]-	187.0965	5.6	0.9979	Azelaic acid	C9H16O4	2266	123-99-9	Fatty Acids and Conjugates	None
33	6.94	[M-H]-	593.1868	0.7	0.9914	Didymin	C28H34O14	16760075	14259-47-3	Flavonoids	Into Blood
34	7.13	[M-H]-	267.0638	0.9	0.996	Formononetin	C16H12O4	5280378	485-72-3	Isoflavonoids	None
35	8.61	[M+Cl]-	983.4475	11.4	0.7366	Compound NP-004089	C46H76O20	10724564	NA	Diterpenoids	Into Blood
36	9.11	[M-H]-	327.217	2.7	0.9895	9,12,13-trihydroxyoctadeca-10	C18H32O5	23872026	185148-53-2	Octadecanoids	None
37	9.78	[M+HCO2]-	515.1912	1.3	0.9959	Evodin	C26H30O8	179651	1180-71-8	Triterpenoids	Into Blood
38	9.93	[M-H]-	329.2328	2.1	0.9922	5,8,11-trihydroxy-9-octadecenoic acid	C18H34O5	24096399	NA	Octadecanoids	None
39	10.46	[M-H]-	448.3063	1.3	0.9982	Glycodeoxycholate	C26H43NO5	3035026	360-65-6	Steroids	Into Blood
40	10.79	[M-H]-	821.395	1.5	0.9875	Licoricesaponin h2	C42H62O16	12889143	NA	Triterpenoids	None
41	11.21	[M-H]-	821.3953	3.2	0.9782	Glycyrrhizic acid	C42H62O16	14982	1405-86-3	Triterpenoids	None
42	11.34	[M+FA-H]-	453.2851	2.4	0.9697	NCGC00380550-01	C24H40O5	60208906	NA	Diterpenoids	None
43	11.83	[M-H]-	391.2848	0.7	0.9993	Ursodeoxycholic acid	C24H40O4	139292057	NA	Steroids	None
44	13.02	[M-H]-	391.2848	0.9	0.9995	chenodeoxycholic acid	C24H40O4	139292058	474-25-9	Steroids	None

### 3.2 Network pharmacology and molecular docking analysis reveal that the FoxO signaling pathway may be a key pathway for CDD treatment of PCOS-IR

**Figure 2:**
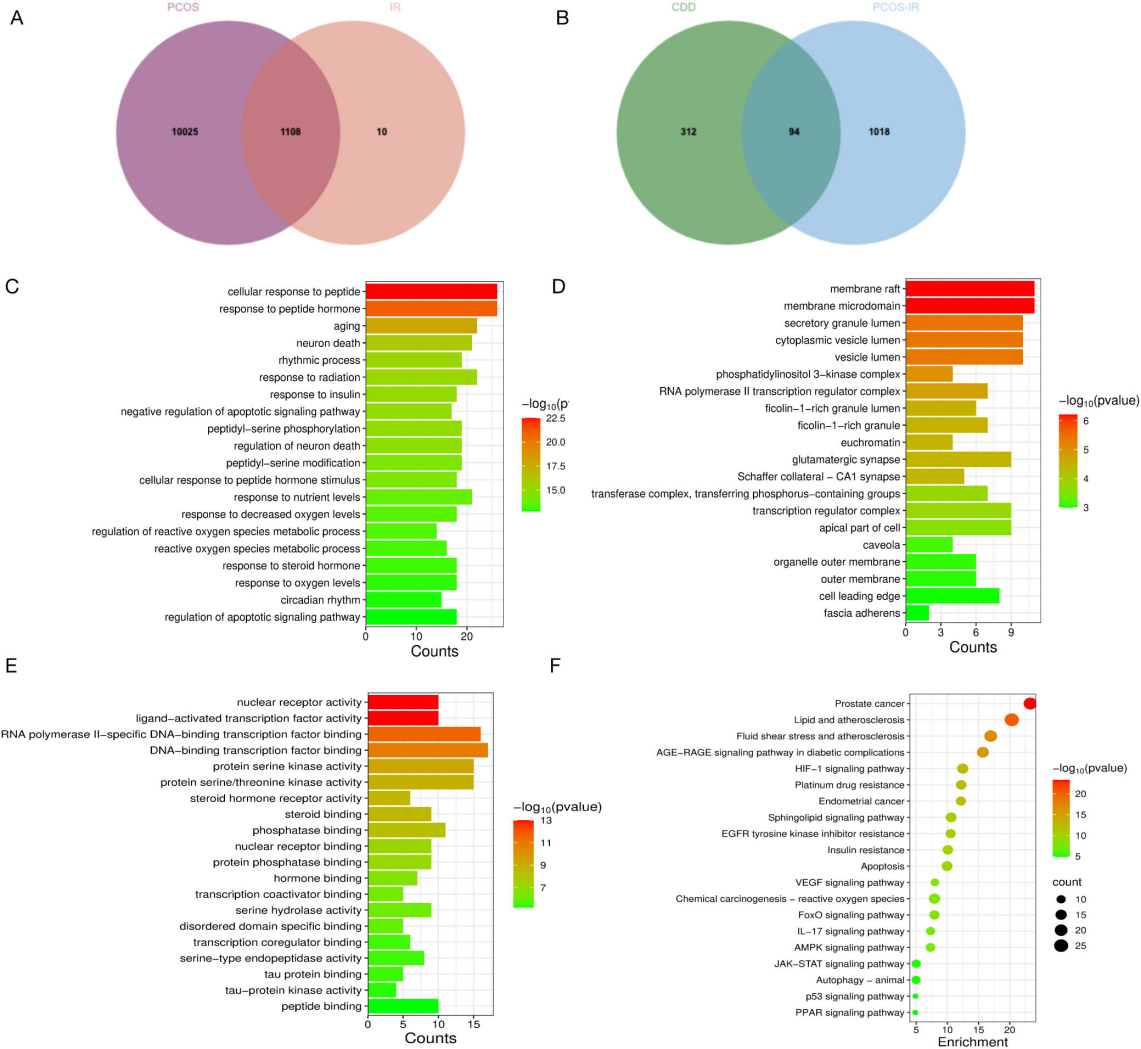
Potential key targets and pathways for CDD treatment of PCOS-IR. **(A)** Venn plot of PCOS genes made with IR genes; **(B)** Venn plot of CDD-predicted targets made with PCOS-IR genes; **(C)** Biological process enrichment results; **(D)** Cellular component enrichment results; **(E)** Molecular function enrichment results; **(F)** KEGG pathway enrichment results.

A total of 1,108 target genes associated with PCOS-IR were identified from the GeneCards, OMIM, and DisGeNET databases (**Figure 2A**). After obtaining the active compounds of CDD, 406 putative targets were predicted using the TCMSP, SuperTarget, ChEMBL, and BindingDB databases. Following the removal of false-positive and duplicate entries, 94 potential targets for CDD in the treatment of PCOS-IR were finally obtained (**Figure 2B**). Furthermore, to elucidate the functional relevance of these targets, Gene Ontology (GO) and Kyoto Encyclopedia of Genes and Genomes (KEGG) enrichment analyses were performed (**Figures 2C–F**). The KEGG enrichment analysis revealed that the CDD-target genes were primarily involved in pathways related to insulin resistance, apoptosis, and autophagy. Additionally, the HIF-1, FoxO, JAK–STAT, and VEGF signaling pathways were significantly enriched (**Figure 2F**).

**Figure 3:**
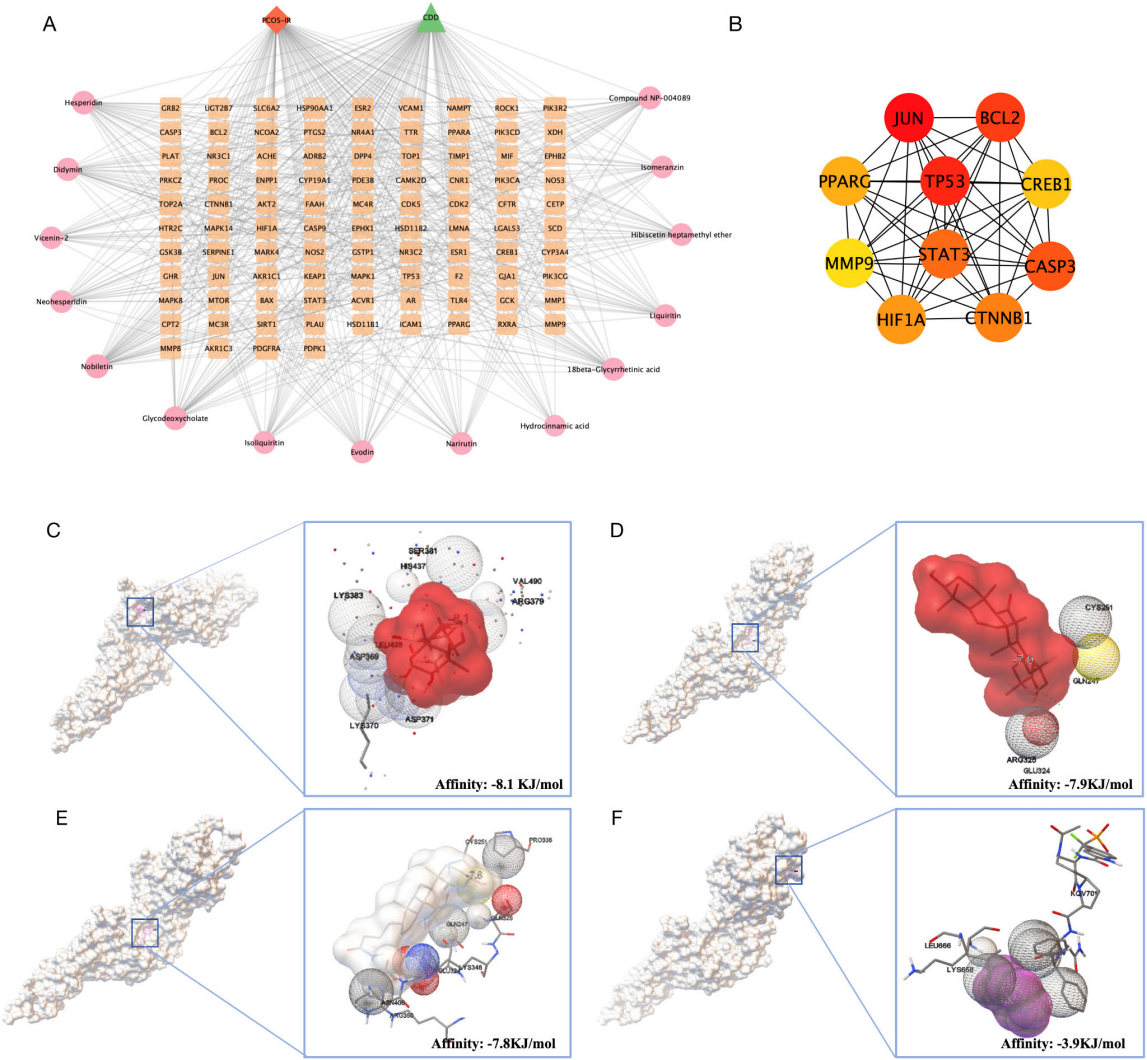
Network pharmacological and molecular docking analysis. **(A)** Disease-active ingredient-target network plot, with squares for target genes, circles for active ingredients, diamonds for disease, and triangle for CDD. Larger nodes indicate closer connections with other nodes; **(B)** Protein-protein interaction network plot of the 10 core targets; **(C)** Schematic diagram of STAT3 docking with 18β-glycyrrhetinic acid, with affinity of -8.1 KJ/mol; **(D)** Schematic diagram of STAT3 docking with Evodin, with affinity of -7.9 KJ/mol; **(E)** Schematic diagramc of STAT3 with glycodeoxycholate, with affinity of -7.8 KJ/mol; **(F)** Schematic diagram of STAT3 with hydrocinnamic acidpachymic acid, with affinity of -3.9 KJ/mol.

The drug-active ingredient-target network was constructed using Cytoscape 3.10.1 (**Figure 3A**). Protein-protein interaction (PPI) networks of the 94 potential targets were generated using the STRING 11.5 database. Subsequently, ten core genes were identified through topological analysis of the PPI network using the CytoHubba plug-in. The key hub genes were *JUN, BCL2, TP53, PPARG, CREB1, MMP9, STAT3, CASP3, HIF1A, CTNNB1* (**Figure3B**). To further explore the molecular interactions, STAT3 was selected as the receptor protein for molecular docking with the representative active compounds of CDD. In molecular docking, a binding energy less than 0 kcal/mol indicates spontaneous binding, and a lower binding energy reflects a more stable ligand–receptor complex (37). The docking results demonstrated that the STAT3 protein exhibited favorable binding affinities with the CDD active compounds 18β-glycyrrhetinic acid (**Figure 3C**), evodin (**Figure 3D**), glycodeoxycholate (**Figure 3E**), and hydrocinnamic acid (**Figure 3F**). These findings indicate that STAT3, involved in both the FoxO and JAK-STAT signaling pathways, may play a key role in the therapeutic effects of CDD on PCOS-IR. These findings suggest that STAT3, a core protein involved in both the FoxO and JAK-STAT signaling pathways, may play a pivotal role in mediating the therapeutic effects of CDD against PCOS-IR. Therefore, the IL6/JAK2/STAT3/FOXO4 signaling pathway centered on STAT3 was selected for subsequent mechanistic investigations.

### 3.4 CDD treatment significantly improved general condition and insulin resistance in PCOS-IR mice

**Figure 4:**
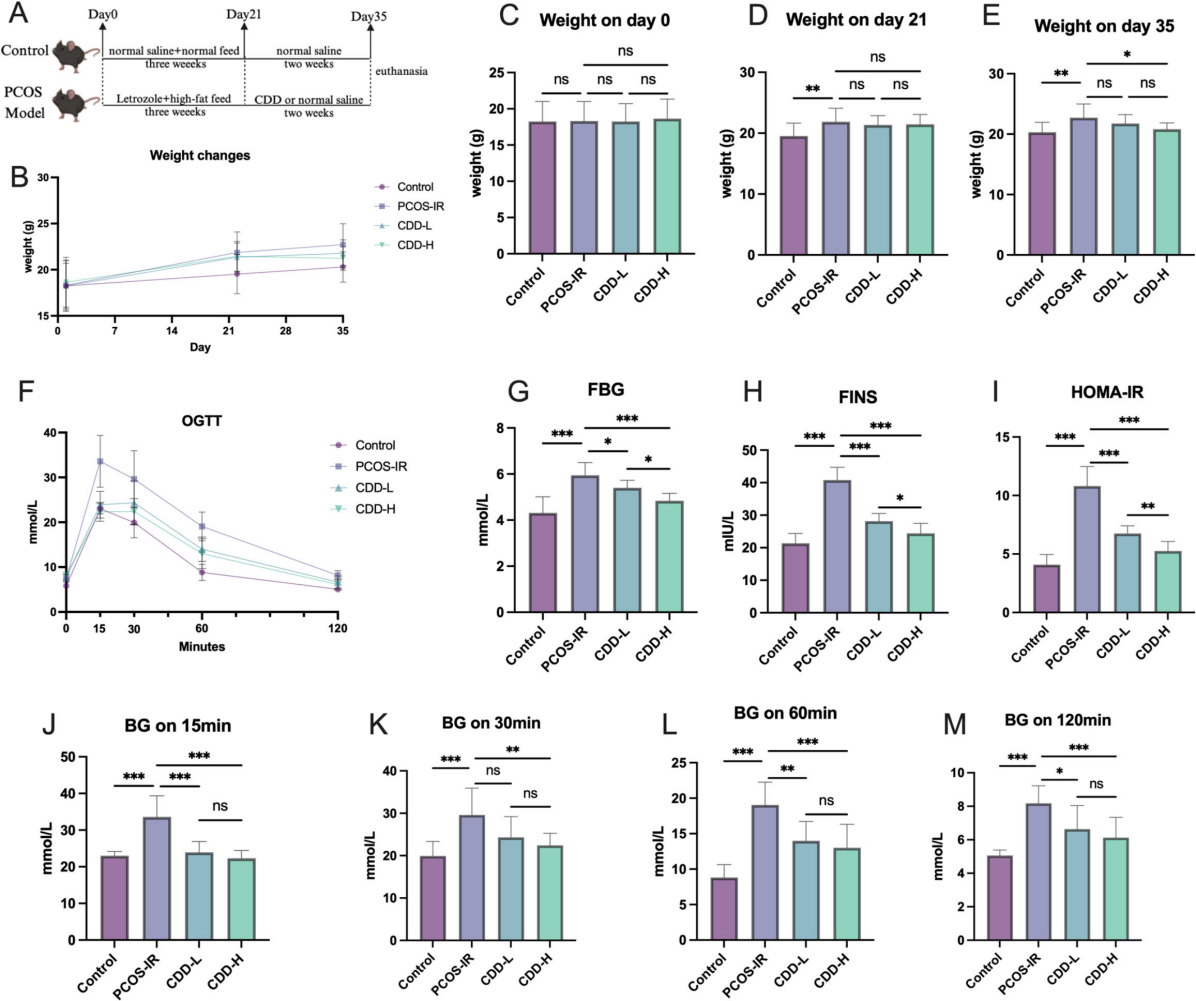
CDD treatment significantly improves general condition and insulin resistance in PCOS-IR model mice **(A)** Schematic diagram of the modeling and intervention procedures used in this study; **(B–E)** Body weight changes and comparisons among groups at different stages (n=15); **(F)** Result of OGTT (n=15); **(G)** Comparison of fasting blood glucose levels (n=15); **(H)** Comparison of fasting insulin levels(n=15); **(I)** Comparison of HOMA-IR index (n=15); **(J-M)** Comparison of blood glucose levels on 15/30/60/120 min during OGTT (n=15). *^ns^P*>0.05, ^*^P < 0.05, ^**^P < 0.01, ^***^P < 0.001.

The experimental procedure for the animal model is illustrated in **Figure 4A**, and the changes in body weight are presented in **Figure 4B**. Prior to the intervention, no significant differences in body weight were observed among the experimental groups (**Figure 4C**). Following model induction, the body weights of mice in the PCOS, CDD-L, and CDD-H groups were significantly higher than those of the control group (**Figure 4D**). At the end of the treatment period, mice in the PCOS-IR group exhibited a significantly higher body weight compared with the control group (*P* < 0.05). In contrast, high-dose CDD treatment markedly reduced body weight compared with the PCOS-IR group (*P* < 0.05; **Figure 4E**). The results of the oral glucose tolerance test (OGTT) are shown in **Figure 4F**. Fasting blood glucose (FBG), fasting insulin levels (FINS), and the HOMA-IR index were all significantly elevated in the PCOS-IR group compared with the control group (*P* < 0.05; **Figures 4G–I**). Treatment with either low-dose or high-dose CDD significantly reduced FBG, FINS, and HOMA-IR compared with the PCOS-IR group (*P* < 0.05; **Figures 4G–I**). Moreover, high-dose CDD significantly decreased blood glucose levels at 15, 30, 60, and 120 min during OGTT compared with the PCOS-IR group (**Figures 4J–M**).

### 3.5 CDD treatment significantly improves endocrine function and ovarian morphology in PCOS-IR mice

**Figure 5:**
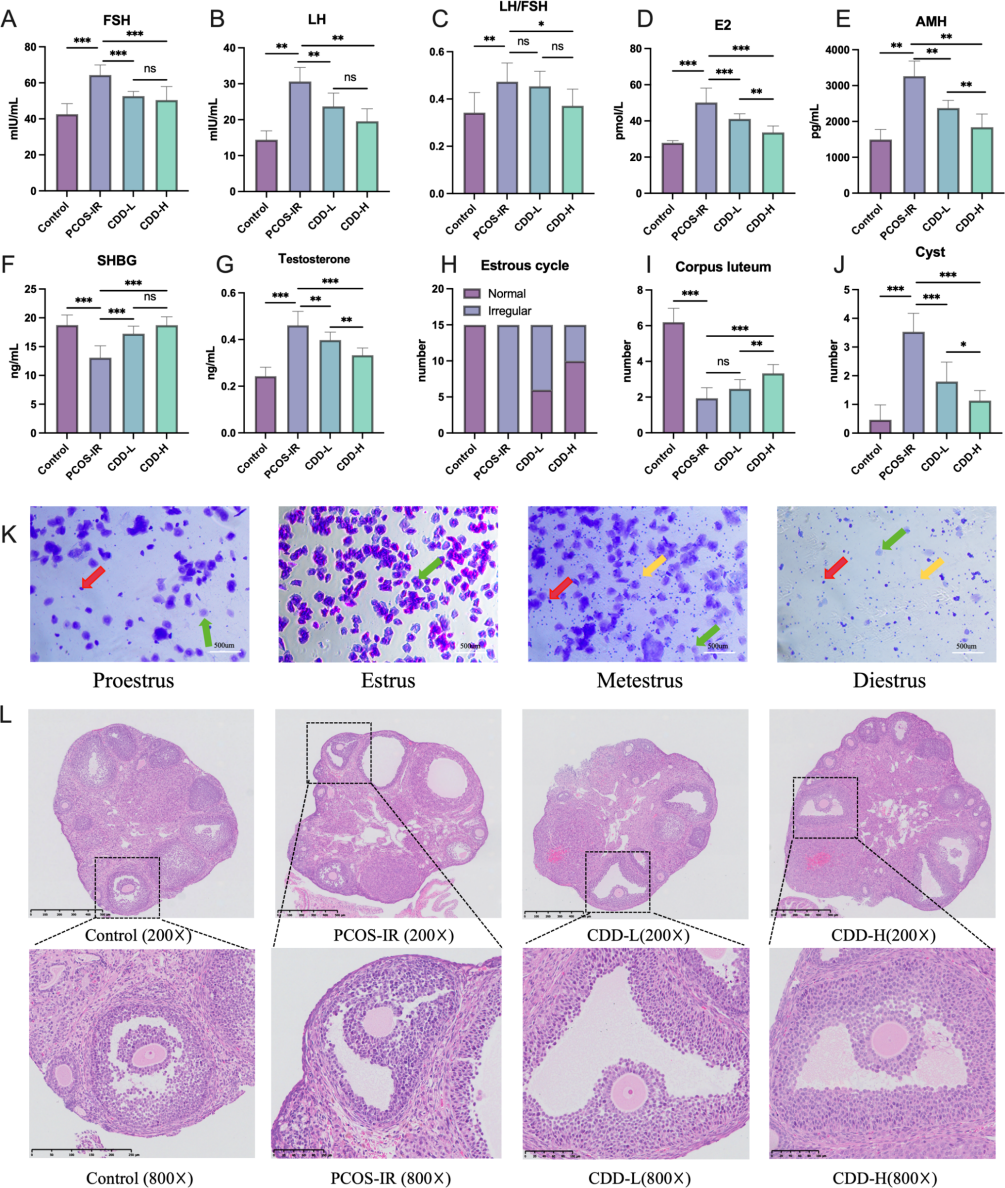
CDD treatment significantly improves endocrine function and ovarian morphology in PCOS-IR model mice **(A–C)** Serum FSH, LH levels and LH/FSH ratio (n=15); **(D, E)** Serum E_2_ and AMH levels(n=15); **(F)** Serum SHBG levels (n=15); **(G)** Serum testosterone levels (n=15); **(H)** Proportion of mice with normal or disrupted estrous cycles after treatment (n=15); **(I, J)** Quantification of cystic follicles and corpora lutea in the ovaries (n=15); **(K)** Representative vaginal smear showing different stages of the estrous cycle: red arrows indicate nucleated epithelial cells, green arrows indicate anucleated keratinized epithelial cells, and yellow arrows indicate leukocytes; The original image is shown in **Supplementary Figures S1–S4**; The pattern of changes in the estrous cycle of each mouse is shown in **Supplementary Table 1**; **(L)** Ovarian histomorphology as shown by H&E staining. The image in the PCOS-IR group represents an ovary undergoing atresia. *^ns^P*>0.05, ^*^P < 0.05, ^**^P < 0.01, ^***^P < 0.001.

To evaluate the endocrine effects of CDD treatment, serum levels of FSH, LH, E_2_, and AMH were measured in each group. Compared with the control group, serum FSH, LH, E_2_, and AMH levels were significantly elevated in the PCOS-IR group (*P* < 0.05). Both low-dose and high-dose CDD treatments markedly reduced the levels of these hormones compared with the PCOS-IR group (*P* < 0.05; **Figures 5A–E**). Furthermore, high-dose CDD significantly decreased the LH/FSH ratio compared with the PCOS-IR group (*P* < 0.05; **Figure 5C**). In addition, the SHBG level was significantly lower in the PCOS-IR group than in the control group (*P* < 0.001; **Figure 5F**). Treatment with either low-dose or high-dose CDD significantly increased SHBG levels compared with the PCOS-IR group (*P* < 0.001). The testosterone level was significantly higher in the PCOS-IR group than in the control group (*P* < 0.001; **Figure 5G**). Treatment with either low-dose or high-dose CDD significantly decreased testosterone levels compared with the PCOS-IR group (*P* < 0.01). Mice in the PCOS-IR group exhibited disrupted estrous cycles, characterized by prolonged or absent estrus phases (**Figures 5H, K**). Following CDD administration, the number of mice with disrupted cycles was reduced, indicating that CDD treatment could help restore normal estrous cyclicity (**Figures 5H, K**). Histological examination of ovarian sections was performed using H&E staining. In the control group, ovarian tissues contained follicles at various developmental stages, displaying well-organized and densely arranged granulosa cell layers as well as abundant corpora lutea. In contrast, ovaries from PCOS-IR mice showed numerous cystically dilated follicles, thinner granulosa cell layers, disorganized cellular structure, and fewer corpora lutea. Compared with the PCOS-IR group, both CDD-L and CDD-H groups markedly increased the number of corpora lutea and reduced cystic follicle formation (*P* < 0.05; **Figures 5I, J, L**).

### 3.6 The therapeutic effect of CDD on PCOS-IR may be associated with the IL6/JAK2/STAT3/FOXO4 pathway

**Figure 6:**
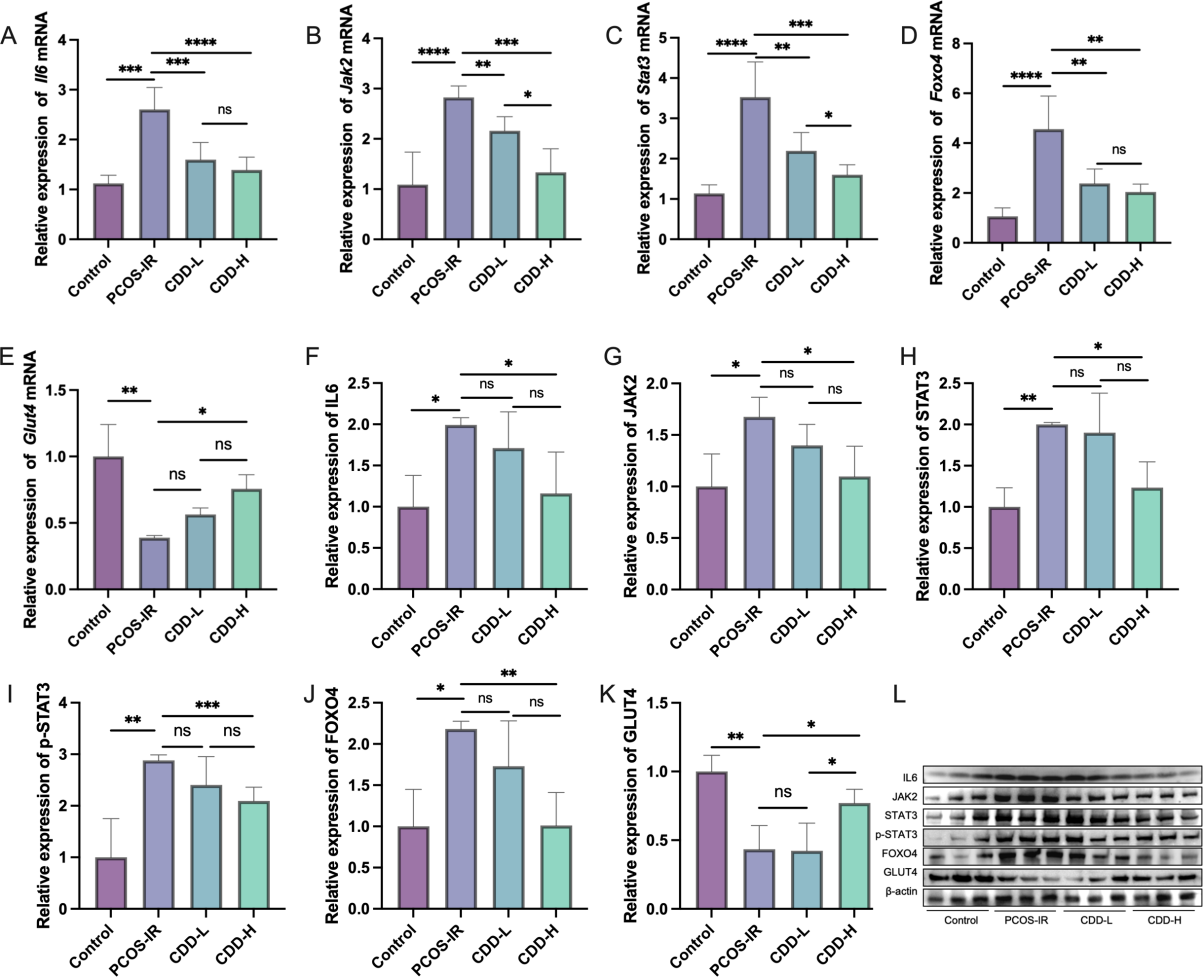
Differential expression of IL6/JAK2/STAT3/FOXO4 in mouse ovarian tissue **(A–E)** The relative mRNA expression levels of *Il6*, *Jak2*, *Stat3*, *Foxo4* and *Glut4* in mouse ovarian tissue were detected by RT-qPCR (n=15); **(F–L)** The relative protein expression levels of IL6, JAK2, STAT3, p-STAT3, FOXO4, and GLUT4 in mouse ovarian tissue were detected by Western blot analysis (n=3). The original western blot bands are shown in **Supplementary Figures S5–S12** in the **Supplementary Materials**. *^ns^P*>0.05, ^*^P < 0.05, ^**^P < 0.01, ^***^P < 0.001,****P < 0.0001.

RT-qPCR and Western blot analyses revealed that the expression levels of IL6, JAK2, STAT3, p-STAT3, and FOXO4 in ovarian tissues were significantly upregulated in the PCOS-IR group compared to the control group (*P* < 0.05) (**Figure 6**). However, the expression level of GLUT4 significantly decreased in the PCOS-IR group (**Figure 6**). In contrast, high-dose CDD treatment significantly downregulated the protein expression levels of IL6, JAK2, STAT3, p-STAT3, FOXO4, while upregulating the expression of GLUT4 compared with the PCOS-IR group (*P* < 0.05; **Figure 6**).

### 3.7 Cellular level reveals that CDD improves granulosa cells’ glucose intake by inhibiting STAT3/FOXO4 signaling pathway

**Figure 7:**
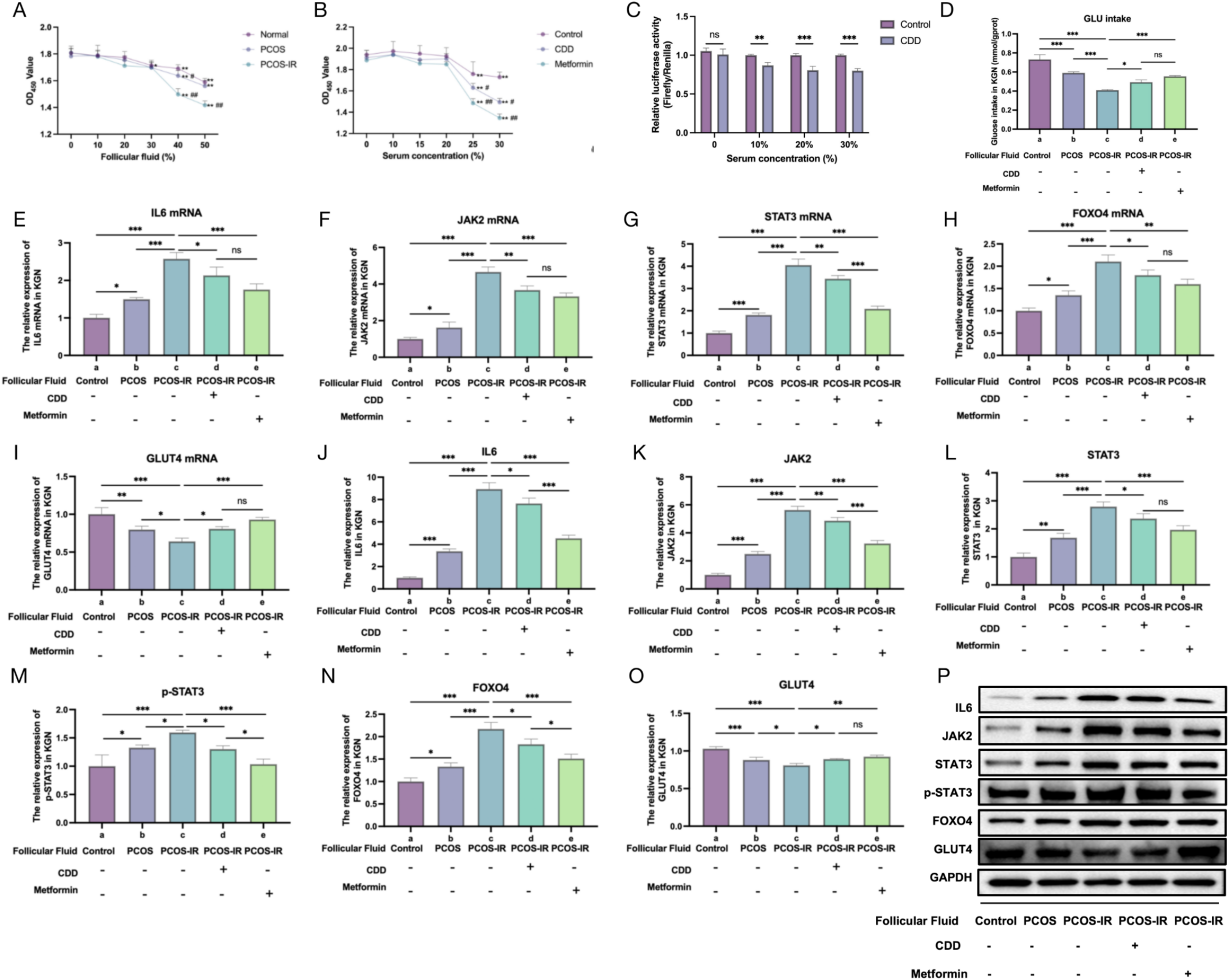
CDD may regulate glucose intake by KGN cells by inhibiting IL6, JAK, STAT3, and FOXO4 mRNA and protein expression levels. **(A, B)** Cell proliferation was measured by CCK-8 assays (n=3). Compared with 0% concentration, ^*^P < 0.05, ^**^P < 0.01. Compared with normal or control group, ^#^*P* < 0.05, ##*P* < 0.01. **(C)** Results of fluorescence reporter gene experiment (n=3). **(D)** Glucose intake of KGN cells in groups a–e. **(E–O)** Expressions of IL6, JAK2, STAT3, FOXO4, and GLUT4 mRNA and their proteins in KGN cells in groups a–e (n=3). **(P)** Western blot bands of IL6, JAK2, STAT3, p-STAT3, FOXO4 and GLUT4 proteins (n=3). The original western blot bands are shown in Supplementary Figures S13–S20 in the Supplementary Materials. *P<0.05, **P<0.01. ^*^P < 0.05, ^**^P < 0.01. The original western blot bands are shown in **Supplementary Figures S13–S20** in the **Supplementary Materials**.

Although an ideal model of ovarian granulosa cell IR is lacking, KGN cells were cultured to simulate the follicular fluid microenvironment of patients. Follicular fluid obtained from oocyte retrieval surgeries of normal infertile patients, patients with PCOS, and patients with PCOS-IR was mixed with culture medium at specified ratios and incubated with KGN cells for 24 h, as previously described (38). Initially, KGN cells were treated with various concentrations of follicular fluid (0%, 10%, 20%, 30%, 40%, and 50%) from the three patient groups for 24 h, and cell viability was assessed. The results showed a concentration-dependent decrease in viability, and 40% follicular fluid was determined to be the optimal concentration for subsequent experiments (**Figure 7A**). Subsequently, rat CDD-containing serum at different concentrations (0%, 10%, 15%, 20%, 25%, and 30%) was applied to KGN cells for 24 h, and cell viability was measured (**Figure 7B**). Additionally, CDD-containing serum induced a dose-dependent reduction in the activity of the *STAT3* promoter–driven reporter gene at concentrations of 0%, 10%, 15%, and 20% (**Figure 7C**). Based on these results, a final serum concentration of 10% was selected for subsequent intervention.

To investigate the relationship between the STAT3/FOXO4 signaling pathway and glucose intake capacity in KGN cells, as well as the effects of CDD intervention, KGN cells were divided into five experimental groups based on the type of follicular fluid and serum treatment: (i) Group a: follicular fluid from normal infertile patients with blank serum (control); (ii) Group b: follicular fluid from patients with PCOS with blank serum; (iii) Group c: follicular fluid from patients with PCOS-IR with blank serum; (IV) Group d: follicular fluid from patients with PCOS-IR with CDD-containing serum; (V) Group e: follicular fluid from patients with PCOS-IR with 20 µM metformin.

Glucose intake was significantly higher in group a than in groups b and c (*P* < 0.05); group c exhibited the lowest (*P* < 0.05); group d was significantly higher than group c (*P* < 0.05); group e was significantly higher than group c (*P* < 0.05; **Figure 7D**). The results indicated that the expression levels of IL6, JAK, STAT3, p-STAT3, and FOXO4 were significantly higher in group c than in groups a and b, whereas GLUT4 expression was markedly reduced (*P* < 0.05; **Figures 7E–P**). Moreover, compared with group c, the expression levels of IL6, JAK, STAT3, p-STAT3, and FOXO4 were significantly decreased in both groups D and E, whereas GLUT4 expression was markedly increased (*P* < 0.05; **Figures 7E–P**). These results suggested that the genes of IL6/JAK2/STAT3/FOXO4 pathway were overexpressed in KGN cells cultured with follicular fluid from patients with PCOS-IR, which might be related to the inhibition of the glucose intake ability of KGN cells. CDD may regulate the glucose intake of KGN cells by inhibiting the expression levels of IL6, JAK, STAT3, and FOXO4.

Next, all KGN cells were cultured in follicular fluid from patients with PCOS-IR to investigate the effects of STAT3 signaling activation and inhibition on glucose uptake. Gene knockdown and activation experiments were performed, and the cells were divided into seven experimental groups as follows: (i) Group a1: blank serum (control); (ii) Group b1: CDD-containing serum; (iii) Group c1: blank serum with 2 µM STAT3 activator (Colivelin TFA); (IV) Group d1: CDD-containing serum with 2 µM STAT3 activator (Colivelin TFA); (V) Group e1: blank serum with si-NC; (VI) Group f1: blank serum with si-*STAT3*; (VII) Group g1: blank serum with si-*FOXO4*.

**Figure 8:**
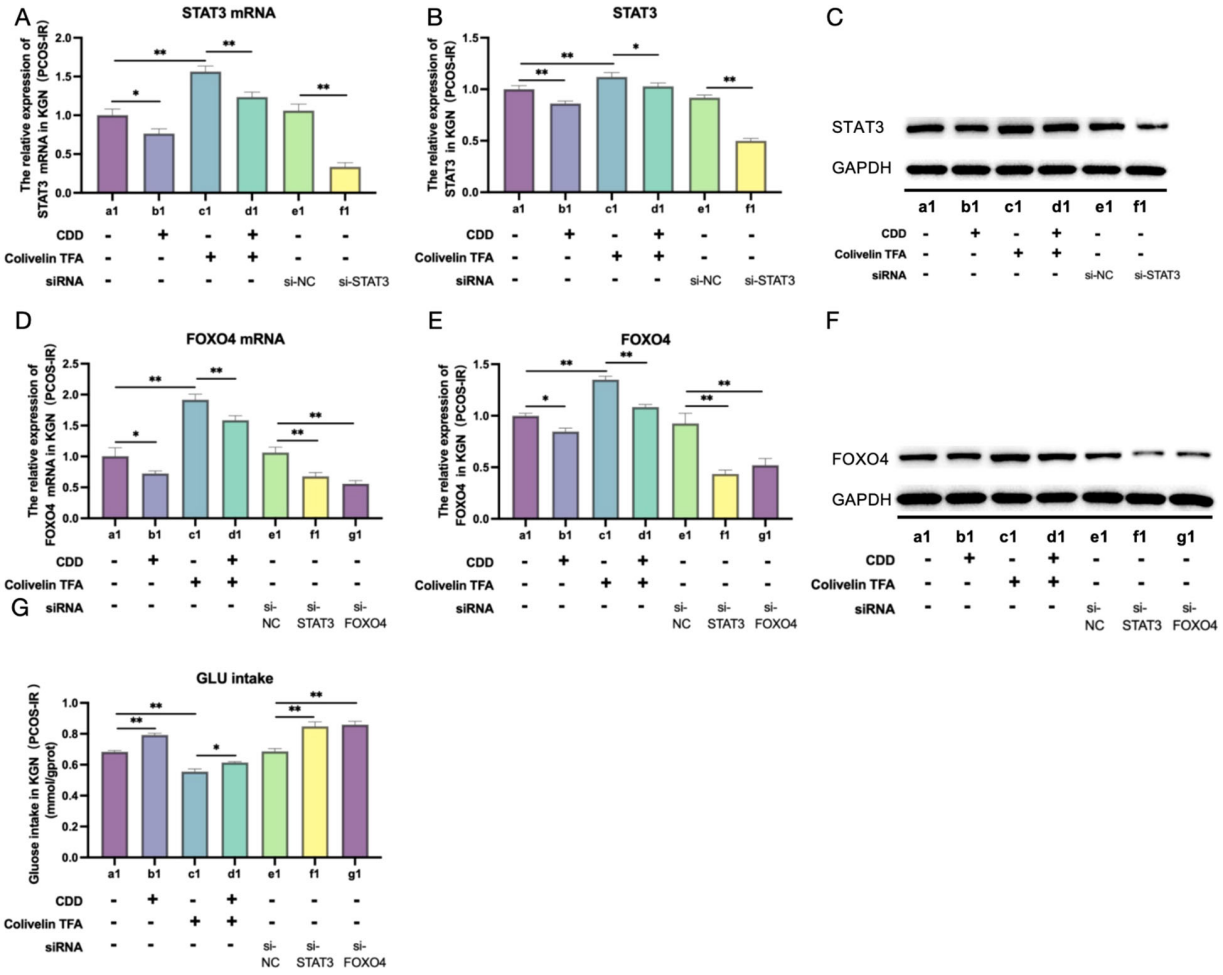
CDD-containing serum promotes glucose intake by KGN cells by inhibiting STAT3/FOXO4 signaling pathway. **(A–C)** Expression of *STAT3* mRNA and its proteins in KGN cells in groups a1–f1 (n=3). The original western blot bands are shown in **Supplementary Figures S21, S22** in the **Supplementary Materials**. **(D–F)** Expression of FOXO4 mRNA and its protein in KGN cells in groups a1–f1(n=3).The original western blot bands are shown in **Supplementary Figures S23, S24** in the **Supplementary Materials**. **(G)** Glucose intake in KGN cells in groups a1–f1(n=3). *^ns^P*>0.05, ^*^P < 0.05, ^**^P < 0.01, ^***^P < 0.001.

The results revealed that compared with group a1, group b1 exhibited a significant decrease in STAT3 and FOXO4 expression levels (*P* < 0.05;**Figures 8A–F**), while glucose intake was significantly higher (*P* < 0.05; **Figure 8G**). Compared with group a1, group c1 exhibited significantly higher STAT3 and FOXO4 expression levels (*P* < 0.05; **Figures 8A–F**), while glucose intake was significantly decreased (*P* < 0.05; **Figure 8G**). Compared with group c1, group d1 exhibited a significant decrease in STAT3 and FOXO4 expression levels (*P* < 0.05; **Figures 8A–F**), while glucose intake was significantly higher (*P* < 0.05; **Figure 8G**). These results suggest that the effect of CDD-containing serum on the glucose intake capacity of KGN cells was mediated by inhibiting STAT3 activation.

Moreover, STAT3 and FOXO4 expression levels were significantly decreased (*P* < 0.05) in group f1 compared to group e1 (**Figures 8A–F**), while glucose intake was significantly elevated (*P* < 0.05; **Figure 8G**). Compared with group e1, group g1 exhibited a significant decrease (*P* < 0.05) in both FOXO4 mRNA and protein expression levels (**Figures 8A–F**), while glucose intake was significantly elevated (*P* < 0.05; **Figure 8G**). These results suggested that blocking the STAT3/FOXO4 signaling pathway could significantly promote glucose intake by KGN cells, and CDD-containing serum could promote glucose intake by KGN cells by inhibiting the STAT3/FOXO4 signaling pathway.

## 4 Discussion

The pathogenesis of PCOS is closely associated with marked insulin resistance, whereas insulin secretion is usually preserved or even compensatorily elevated. IR is strongly linked to ovarian dysfunction, inflammation, hyperandrogenism, and metabolic disturbances. Factors such as diet, lifestyle, and gut microbiota influence IR (39–41), and modifying these factors is thought to ameliorate metabolic dysfunction in PCOS. Therefore, identifying agents that effectively improve fertility while minimizing adverse effects is of great importance. Previous studies have indicated that CDD can enhance *PKP3* promoter methylation to reduce PKP3 expression (18), downregulate *FOXK1* (42), inhibit the Wnt/β-Catenin signaling pathway (17), inhibit the ASK1/JNK pathway (43), inhibit granulosa cell apoptosis, and play a restorative role in ovarian morphology. Besides, CDD has a significant effect on PCOS-IR in clinically obese patients, particularly regarding weight loss (16). In animal experiments, CDD regulated lipid metabolism, hormone secretion, and inflammatory responses and improved rat abdominal fat percentage (15, 44). In contrast to these clinical observations focusing on systemic endocrine regulation, our study explored the granulosa-cell-specific mechanism underlying CDD action in PCOS-IR. We identified the IL6/JAK2/STAT3/FOXO4 axis as a novel signaling pathway mediating the local ovarian effects of CDD, providing a new perspective that links its anti-inflammatory and insulin-sensitizing actions at the cellular level.

First, the active compounds of CDD were analyzed by UPLC-HRMS, and 15 active compounds were identified. Ovarian granulosa cells provide nutrients and metabolites to oocytes through gap junctions and secrete paracrine signals that regulate oocyte development and maturation (45). The KEGG enrichment analysis revealed that the CDD-target genes were primarily involved in insulin signaling, JAK-STAT signaling pathway, FoxO signaling pathway, and HIF-1 signaling pathway were also significantly enriched. Among them, 20 genes such as *IL6* (high expression), *JAK2* (high expression), *STAT3*(high expression), and *FOXO1* (high expression) were screened as core genes. After further database prediction of the target genes of 15 active compounds in CDD, we found that STAT3 protein docked well with the effective active ingredients of CDD. The above results prompted us to further investigate the role of IL6/JAK2/STAT3/FOXO signaling pathway in the regulation of granulosa cell function. The FoxO family, consisting of FOXO1, FOXO3, FOXO4, and FOXO6, is widely expressed in mammalian tissues and plays important roles in metabolism, cell proliferation, apoptosis, and stress resistance. Extensive research has focused on FOXO1, a key downstream molecule of the insulin/insulin-like growth factor 1 (IGF-1) signaling pathway, which regulates systemic metabolism and hormone levels in the liver, pancreas, hypothalamic–pituitary axis, and adipose tissue by modulating circulating glucose levels (46–48). Notably, FOXO4 acts as a responsive transcription factor of the INS/IGF-1 pathway, regulating proteasome activity in human embryonic stem cells (49) and modulating mitochondrial uncoupling protein 3 expression in skeletal myoblasts via phosphorylation (50). As an upstream target of FOXO4, IL6/STAT3 signaling activation promotes insulin resistance in adipose tissue and muscle (51–53), and JAK2/STAT3 activation induces insulin resistance in HepG2 cells (54). In PCOS studies, troxerutin troxerutin has been shown to attenuate dihydrotestosterone-induced insulin resistance in rats by inhibiting IL-22/JAK1/STAT3 signaling activation (55).

In our study, CDD effectively improved body weight, insulin resistance index, and ovarian function in PCOS-IR model mice, and significantly downregulated both mRNA and protein expression levels of the IL6/JAK2/STAT3/FOXO4 signaling pathway. In the *in vitro* experiments, we simulated an insulin-resistant environment by culturing KGN cells with follicular fluid collected from PCOS-IR patients. Follicular fluid from PCOS patients contains various abnormal components, such as elevated androgen and insulin levels, as well as altered concentrations of cytokines and growth factors (56, 57). These aberrant components can directly affect cellular processes such as growth, differentiation, and metabolism, thereby providing a relevant biochemical microenvironment to model insulin resistance. KGN cells are responsive to a wide range of hormones and cytokines, and thus serve as a suitable model to mimic the response of granulosa cells to the abnormal follicular microenvironment in PCOS (36, 58). We found that KGN cells cultured with follicular fluid from PCOS-IR patients exhibited overexpression of IL6/JAK2/STAT3/FOXO4 pathway genes, whereas inhibition of this pathway significantly enhanced glucose intake. CDD intervention significantly downregulated the expression of IL6/JAK2/STAT3/FOXO4 pathway genes in KGN cells and reversed the upregulation of *STAT3* and *FOXO4* gene expression caused by the STAT3 activator Colivelin TFA, consistent with the suppression of *STAT3* and *FOXO4* gene expression induced by si-*STAT3* and si-*FOXO4*, which collectively demonstrated that CDD promotes the intake of glucose by KGN cells. Previous studies have shown that total flavonoids extracted from Nervilia Fordiiy can inhibit activation of the JAK2/STAT3 pathway in the ovaries of PCOS-IR rats, significantly increase serum FSH levels, and dramatically decrease LH, testosterone, and INS levels (59). Inhibition of the p-JAK2/p-STAT3 signaling pathway has been reported to promote follicular development in PCOS rats. In our study, CDD significantly inhibited the same pathway and improved ovarian morphology and hormone balance, suggesting a potentially similar regulatory mechanism.

Molecular docking in this study suggested that several CDD-derived compounds might directly interact with STAT3. Previous studies have reported that 18β- glycyrrheetinic acid, can significantly inhibit the STAT3 signaling pathway and reduce p-STAT3 levels (60), while certain glycodeoxycholates have been reported to be associated with STAT3 activation *in vitro* (61). However, given the complex and multi-component nature of traditional Chinese medicine formulas, the pharmacological activity of CDD cannot be ascribed to a single constituent. To better capture the integrated biological effects of CDD and its metabolites, we employed CDD-containing serum for validation experiments. Dual-luciferase reporter assays revealed that CDD-containing serum dose-dependently suppressed STAT3 promoter-driven transcriptional activity, accompanied by a marked reduction in p-STAT3 protein levels *in vitro*. Consistently, in ovarian tissues from PCOS-IR model mice, CDD treatment significantly downregulated p-STAT3 expression. These findings provide functional evidence supporting our molecular docking results and confirm that CDD effectively inhibits STAT3 signaling both *in vitro* and *in vivo*.

Notably, although CDD appears to directly interact with STAT3, the observed *in vivo* reductions in IL6 and JAK2 expression, both upstream activators of STAT3, are consistent with this interaction. They likely reflect the broader, integrative pharmacological actions of CDD. It is also possible that the reduced IL6 expression observed *in vivo* partly results from decreased adiposity and systemic inflammation following CDD treatment. As a multi-target herbal formulation, CDD may simultaneously modulate several interconnected pathways, including the suppression of NF-κB signaling, induction of SOCS proteins, and downregulation of pro-inflammatory cytokine expression (15). These combined effects collectively reduce IL6 synthesis and consequently attenuate JAK2/STAT3 activation (62, 63). In the *in vitro* experiments, STAT3 was selected for silencing because it represents the central intracellular hub of IL6 signaling and directly mediates downstream transcriptional responses to cytokine activation. The observed regulation of FOXO4 further suggests potential crosstalk between STAT3 and FOXO signaling pathways. Although FOXO4 is not a canonical downstream effector of IL6, recent studies have reported interactions between STAT3 and FOXO transcription factors in the regulation of oxidative stress, metabolism, and cell survival (64, 65). This may indicate indirect or compensatory signaling interplay rather than a direct IL6-FOXO4 linkage, highlighting the complex network through which CDD exerts its therapeutic effects in PCOS.

This study has some limitations. The small sample size may partially reflect the characteristics of the entire patient population. Future studies should consider expanding the sample size, and improving cellular models to validate the therapeutic effect of CDD on PCOS-IR and explore the related mechanisms and potential targets. In addition, the present study included only two dosage levels of CDD (1× and 3× the clinical equivalent dose). Although these doses were selected based on clinically relevant conversion ratios and preliminary efficacy data, the absence of an intermediate dose limits our ability to fully characterize dose-response relationships. Future studies will include additional dose gradients to better determine the pharmacological range and safety profile of CDD *in vivo*. Furthermore, a positive control group, such as metformin treatment, was not included in the *in vivo* experiments due to ethical and financial constraints. Nevertheless, we acknowledge that the letrozole-induced phenotype may exhibit partial reversibility over longer periods, and future studies will extend the induction or observation duration to further confirm the long-term stability of this model. Future animal studies will incorporate a metformin-treated group to more comprehensively evaluate the relative therapeutic efficacy of CDD. Due to technical and financial limitations, this study did not include combined treatments of CDD following STAT3 or FOXO4 silencing, nor did it involve IL-6 silencing experiments. Future work will address these limitations to clarify the direct signaling mechanisms through which CDD exerts its effects.

**Figure 9:**
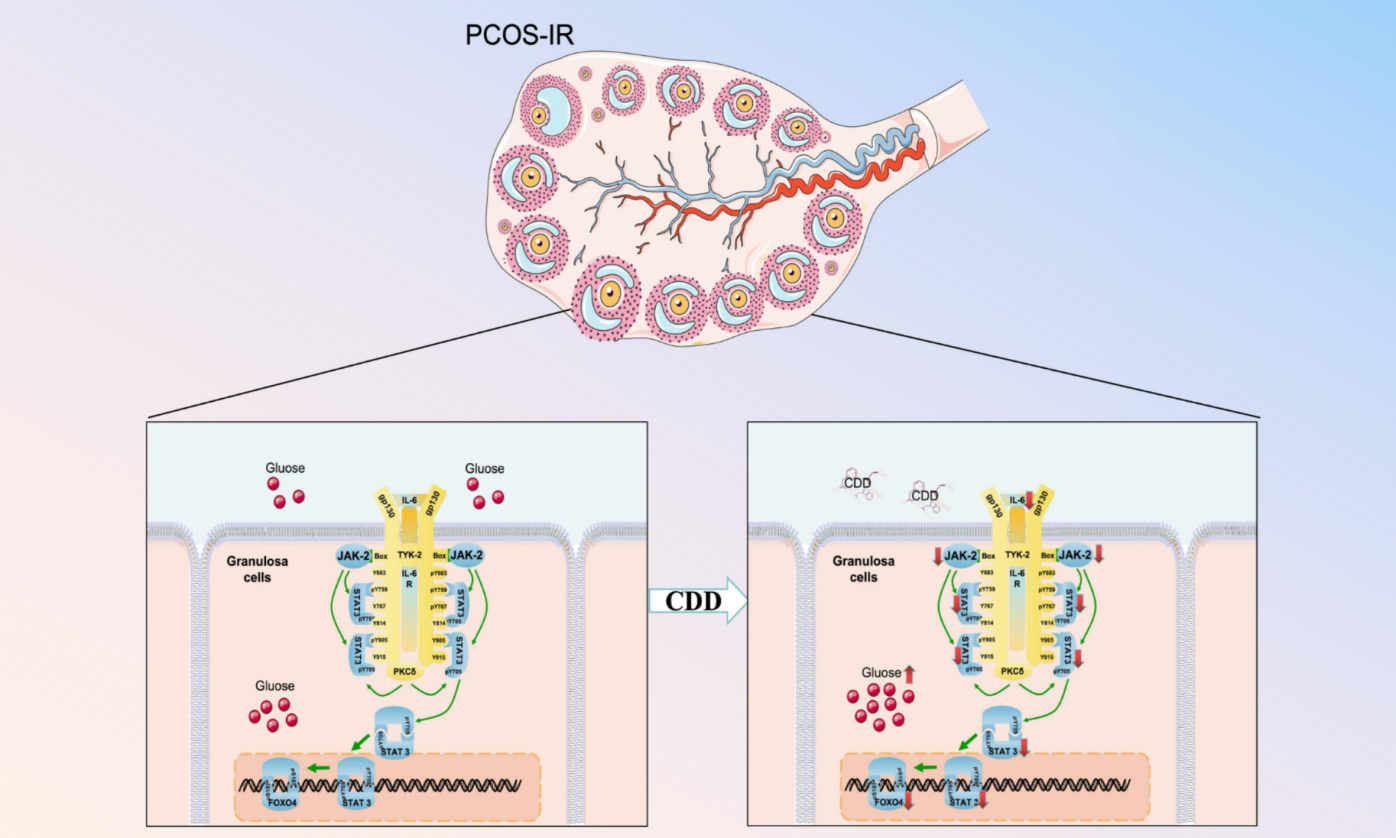
Schematic diagram of the mechanism by which CDD improves PCOS-IR through the IL6/JAK2/STAT3/FOXO4 pathway.

In summary, CDD may improve ovarian function and insulin resistance in PCOS mouse by regulating the IL6/JAK2/STAT3/FOXO4 signaling pathway (**Figure 9**). These findings offer new insights into investigating the mechanisms of intervention in PCOS-IR with CDD and may contribute to developing future intervention strategies for this disease.

## 5 Conclusion

As an alternative and complementary therapy, Cangfu Daotan Decoction may improve ovarian function and ameliorate insulin resistance in PCOS mice by modulating the IL6/JAK2/STAT3/FOXO4 signaling pathway.

## Data Availability

The original contributions presented in the study are included in the article/**Supplementary Material**. Further inquiries can be directed to the corresponding authors.
