# Supplementary figures and images for: Cangfu Daotan decoction treats PCOS-IR through the IL6/JAK2/STAT3/FOXO4 signaling pathway
Source: Front Endocrinol (Lausanne). 2026 Jan 7;16:1661000. doi: 10.3389/fendo.2025.1661000 (PMC12819200; doi:10.3389/fendo.2025.1661000)

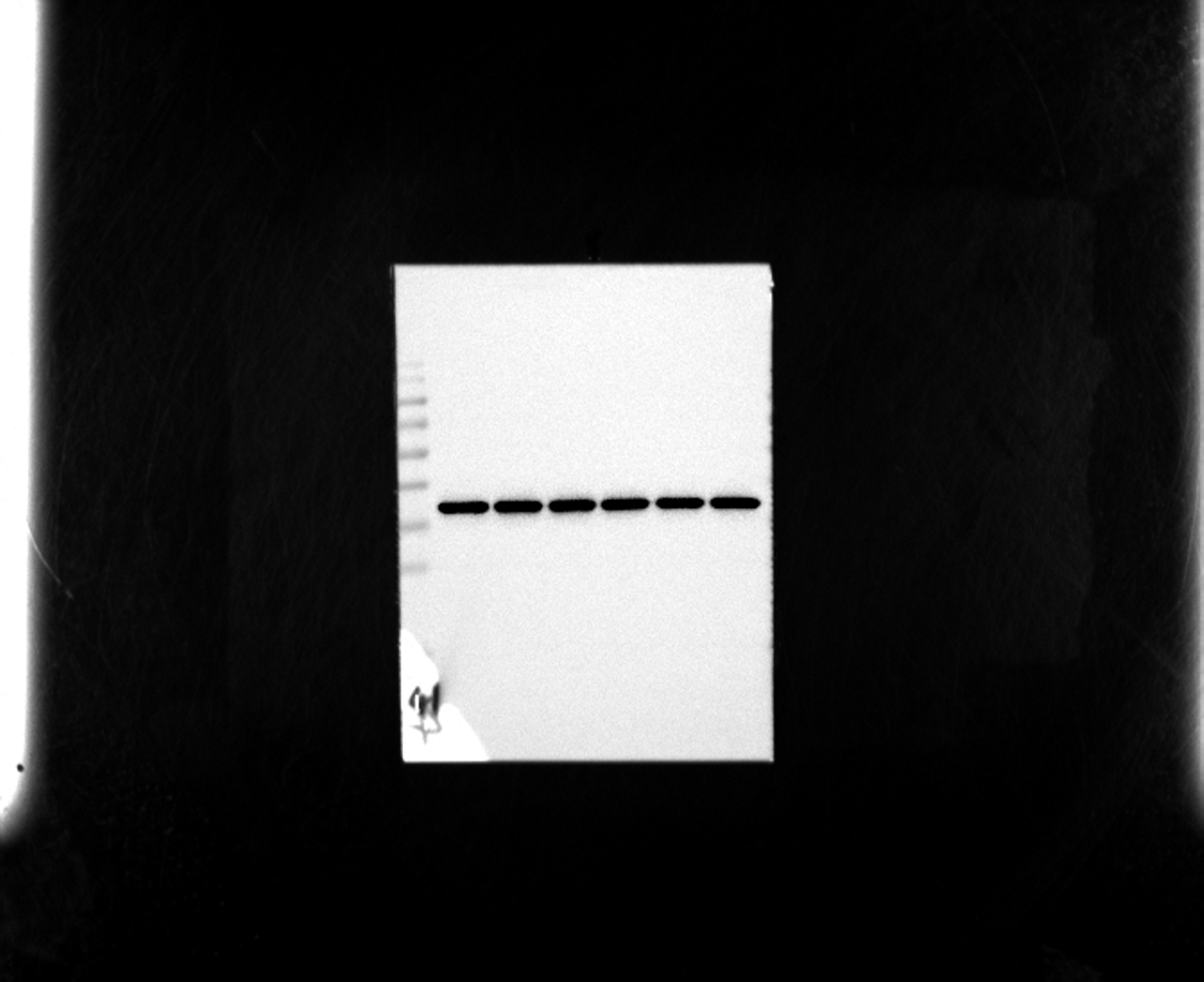

Supplement: Supplementary file 1 [file DataSheet1.zip › Supplementary Figure S5-24/Figure S22 GAPDH .Tif]

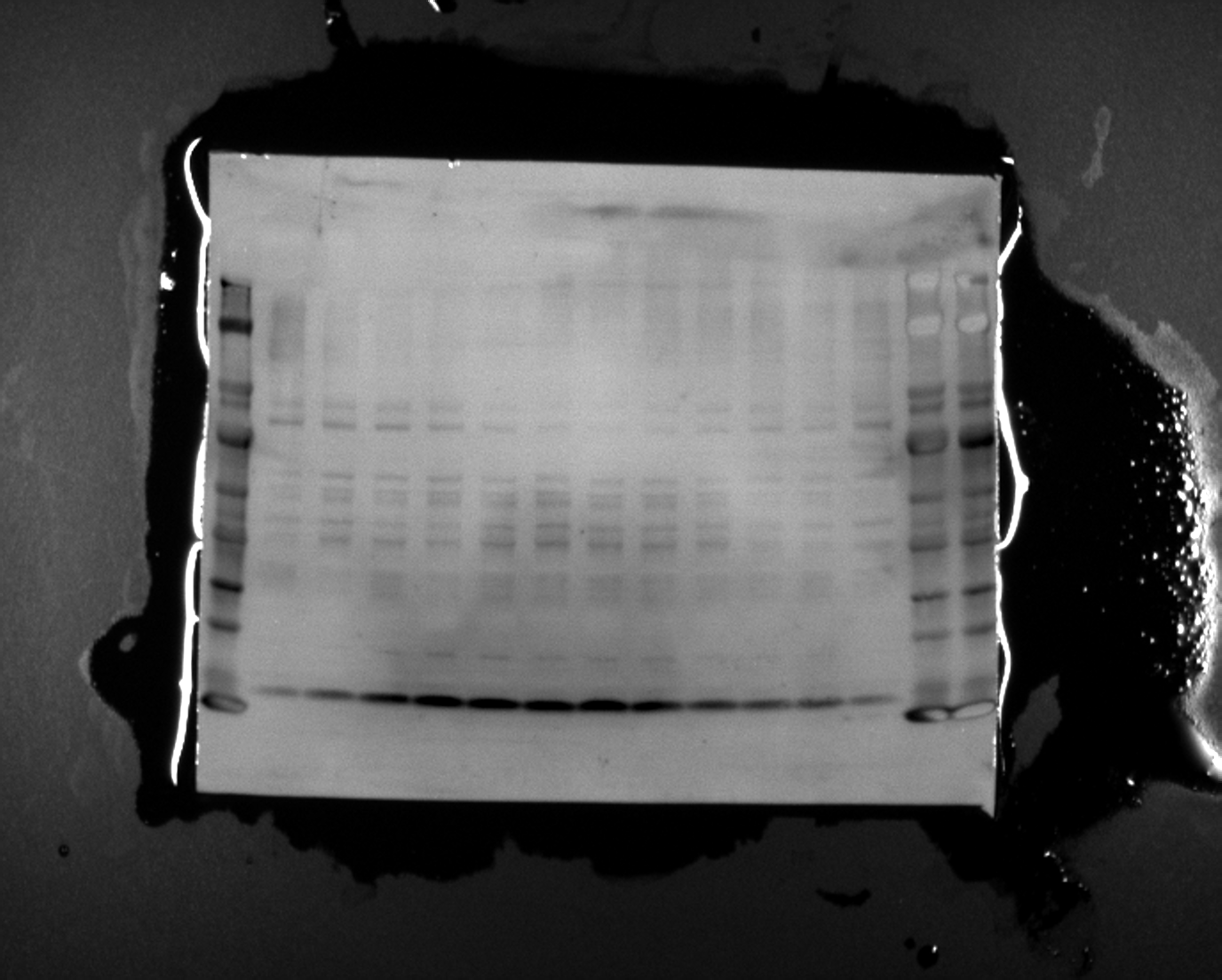

Supplement: Supplementary file 1 [file DataSheet1.zip › Supplementary Figure S5-24/Figure S5 IL6.Tif]

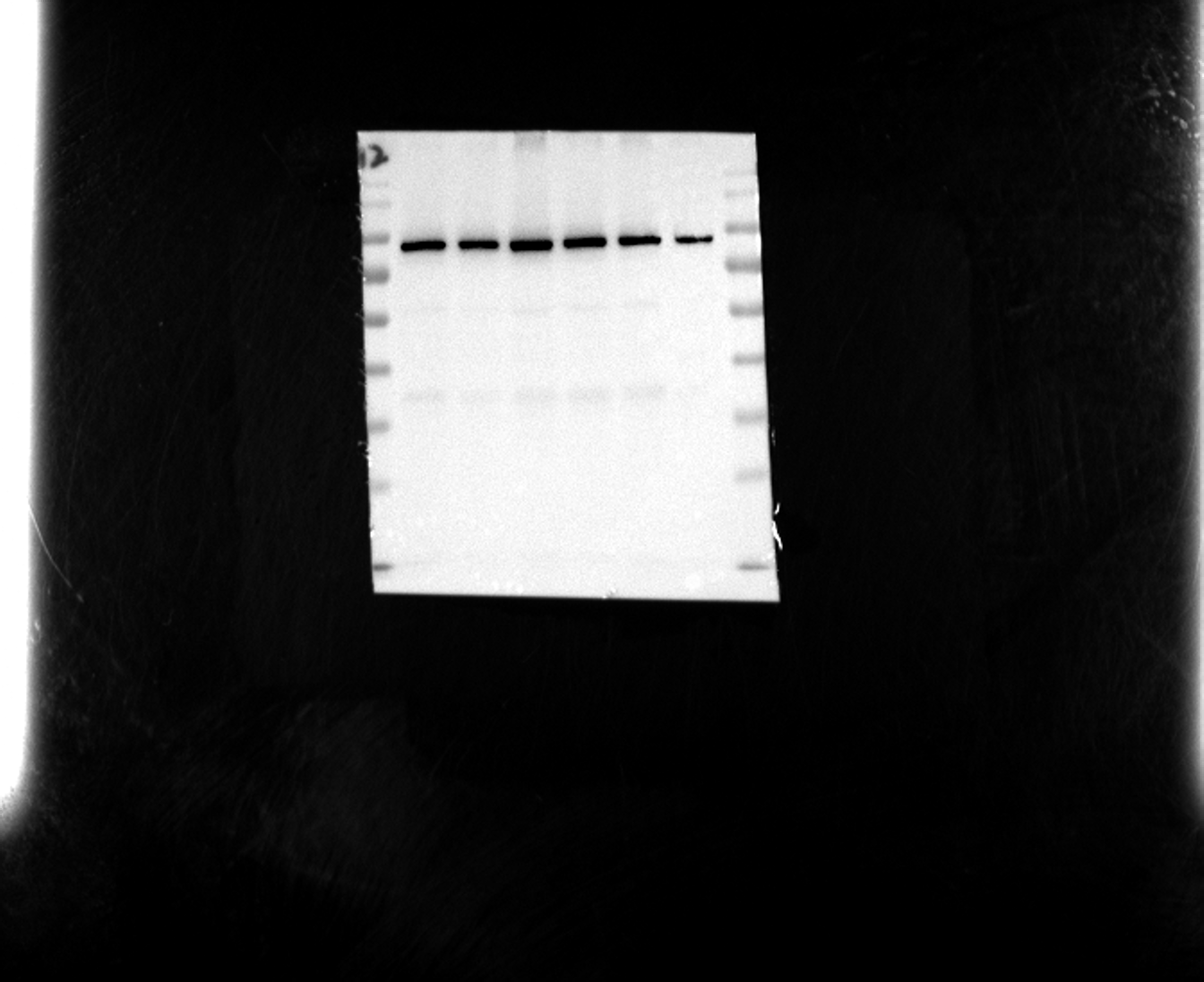

Supplement: Supplementary file 1 [file DataSheet1.zip › Supplementary Figure S5-24/Figure S21 STAT3.Tif]

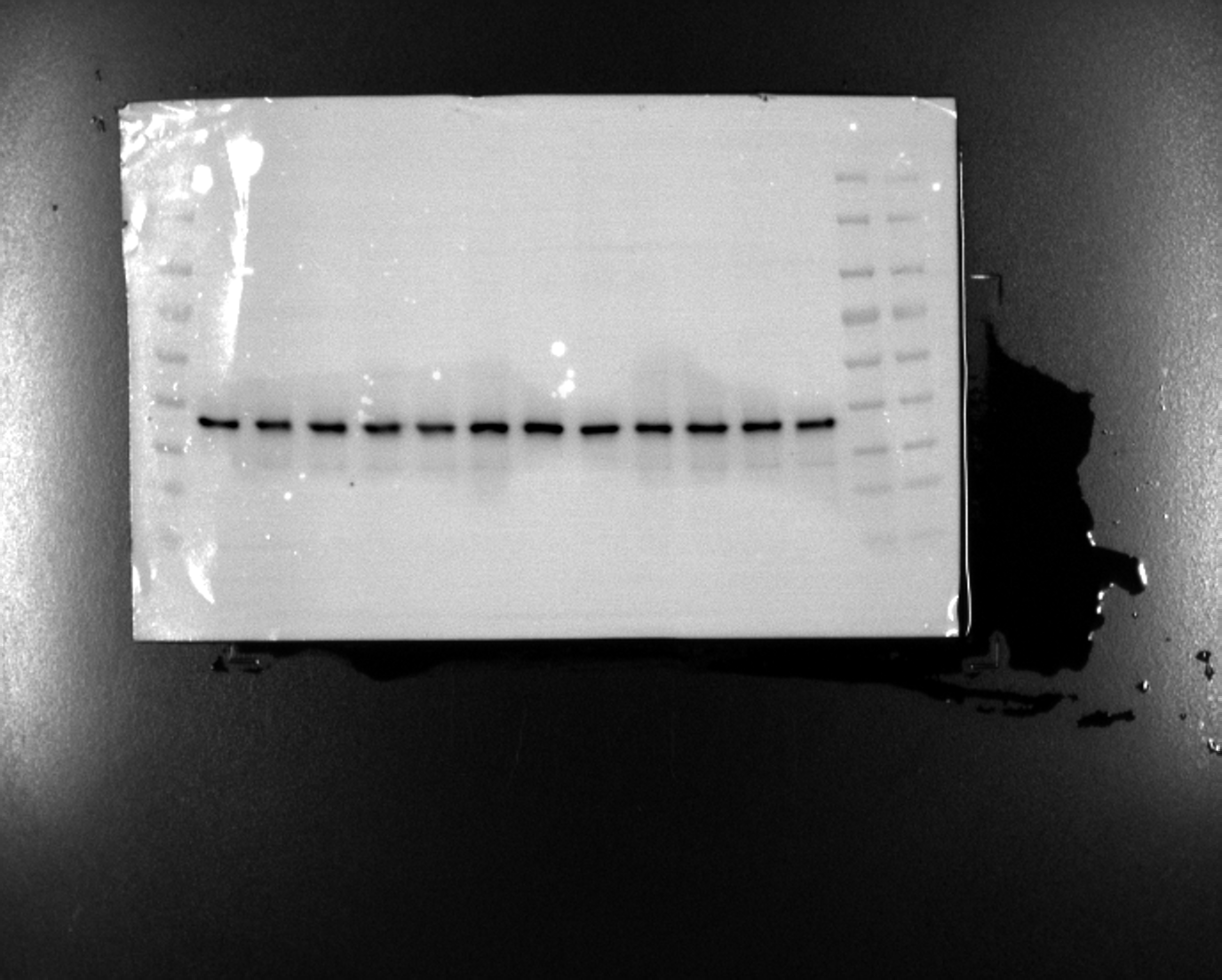

Supplement: Supplementary file 1 [file DataSheet1.zip › Supplementary Figure S5-24/Figure S12 GAPDH.Tif]

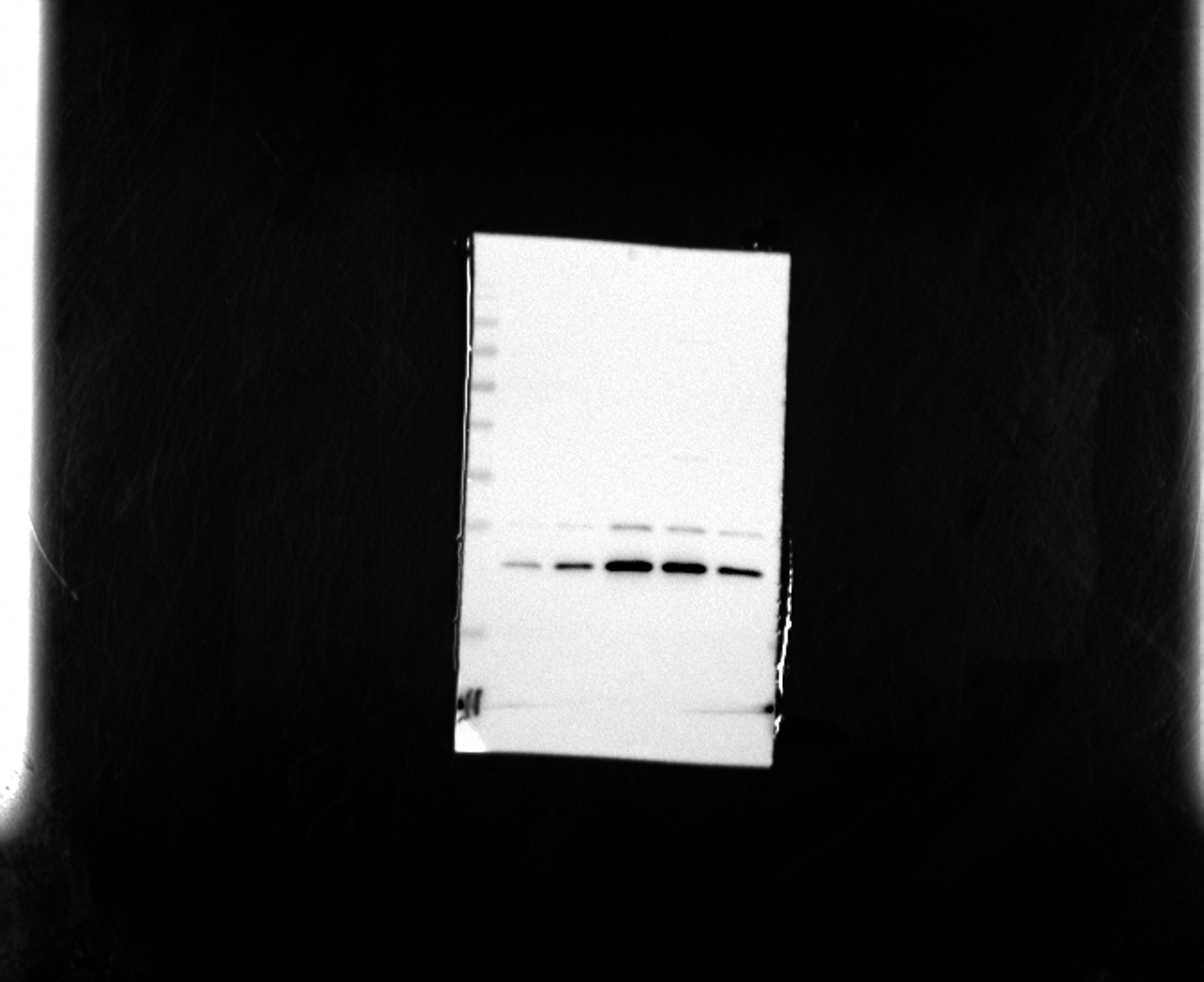

Supplement: Supplementary file 1 [file DataSheet1.zip › Supplementary Figure S5-24/Figure S13 IL6.Tif]

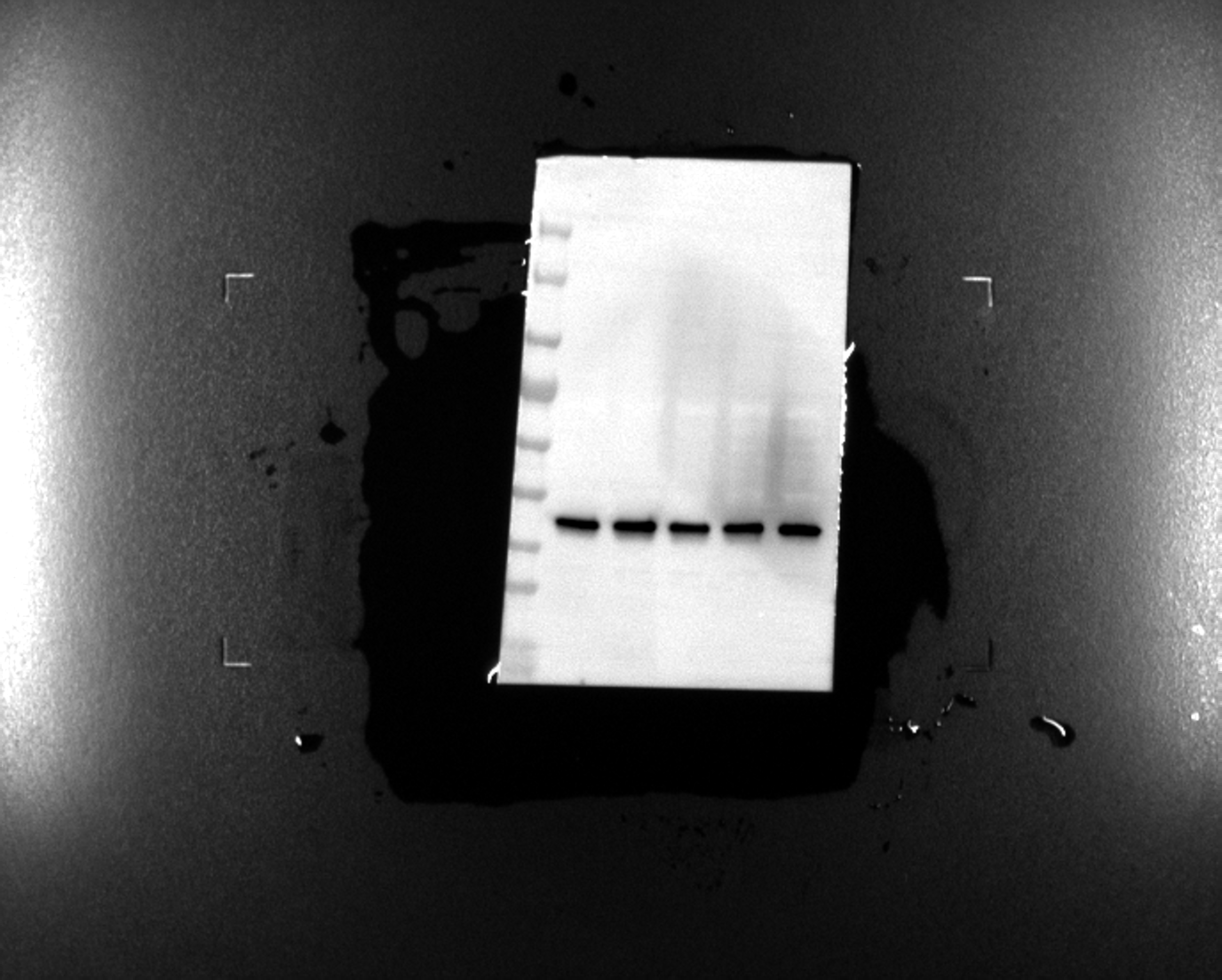

Supplement: Supplementary file 1 [file DataSheet1.zip › Supplementary Figure S5-24/Figure S20 GAPDH.Tif]

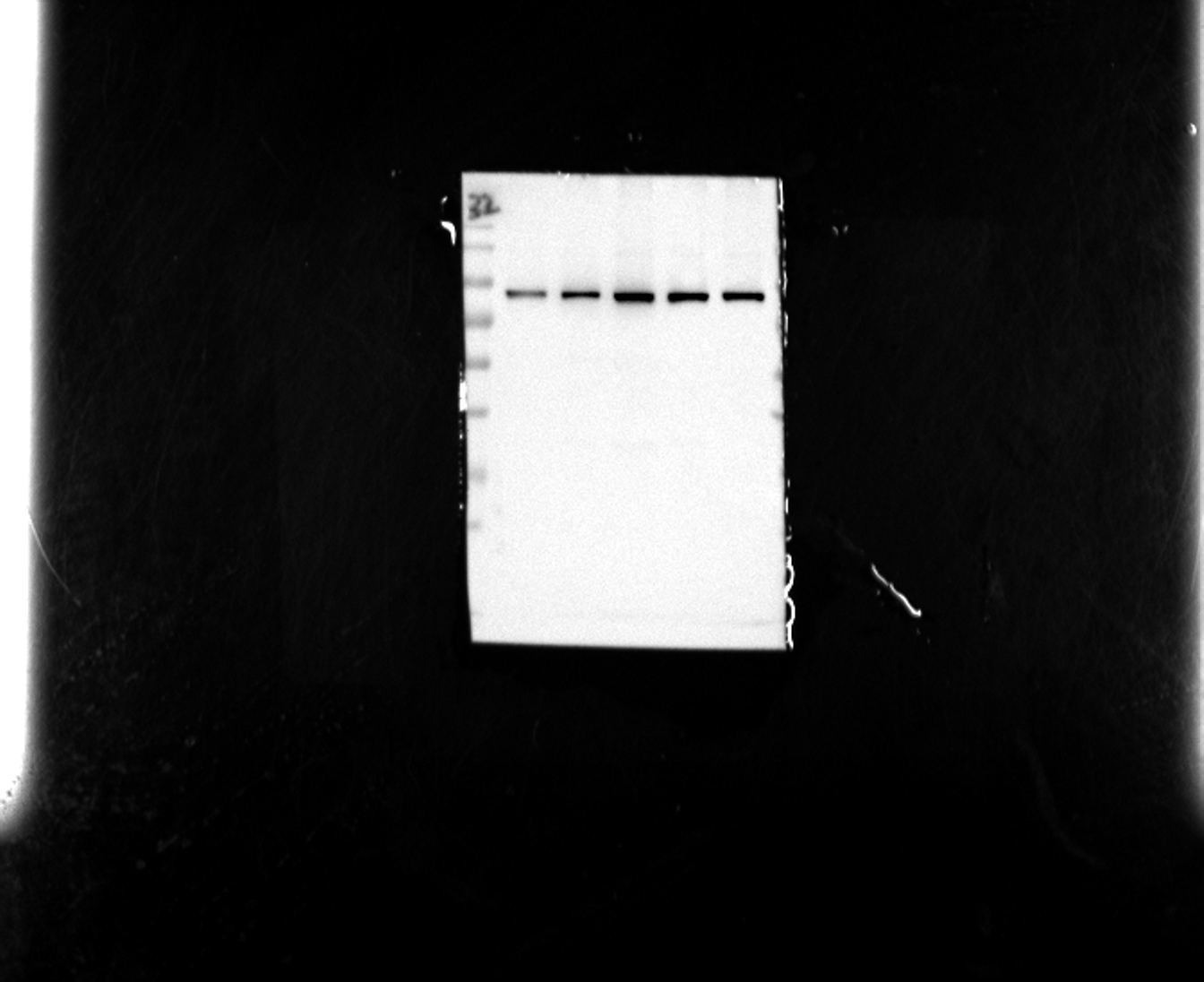

Supplement: Supplementary file 1 [file DataSheet1.zip › Supplementary Figure S5-24/Figure S15 STAT3.Tif]

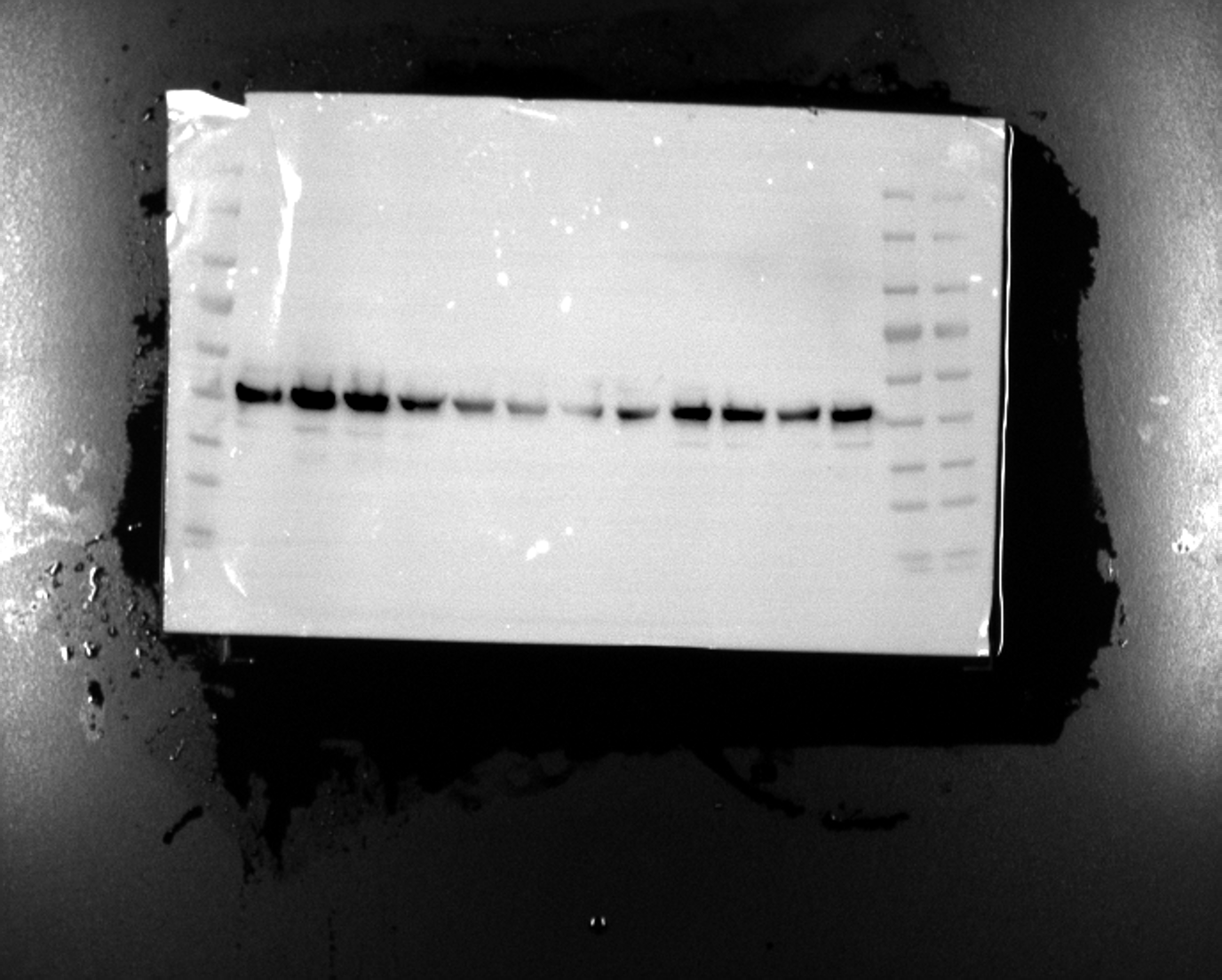

Supplement: Supplementary file 1 [file DataSheet1.zip › Supplementary Figure S5-24/Figure S11 GLUT4.Tif]

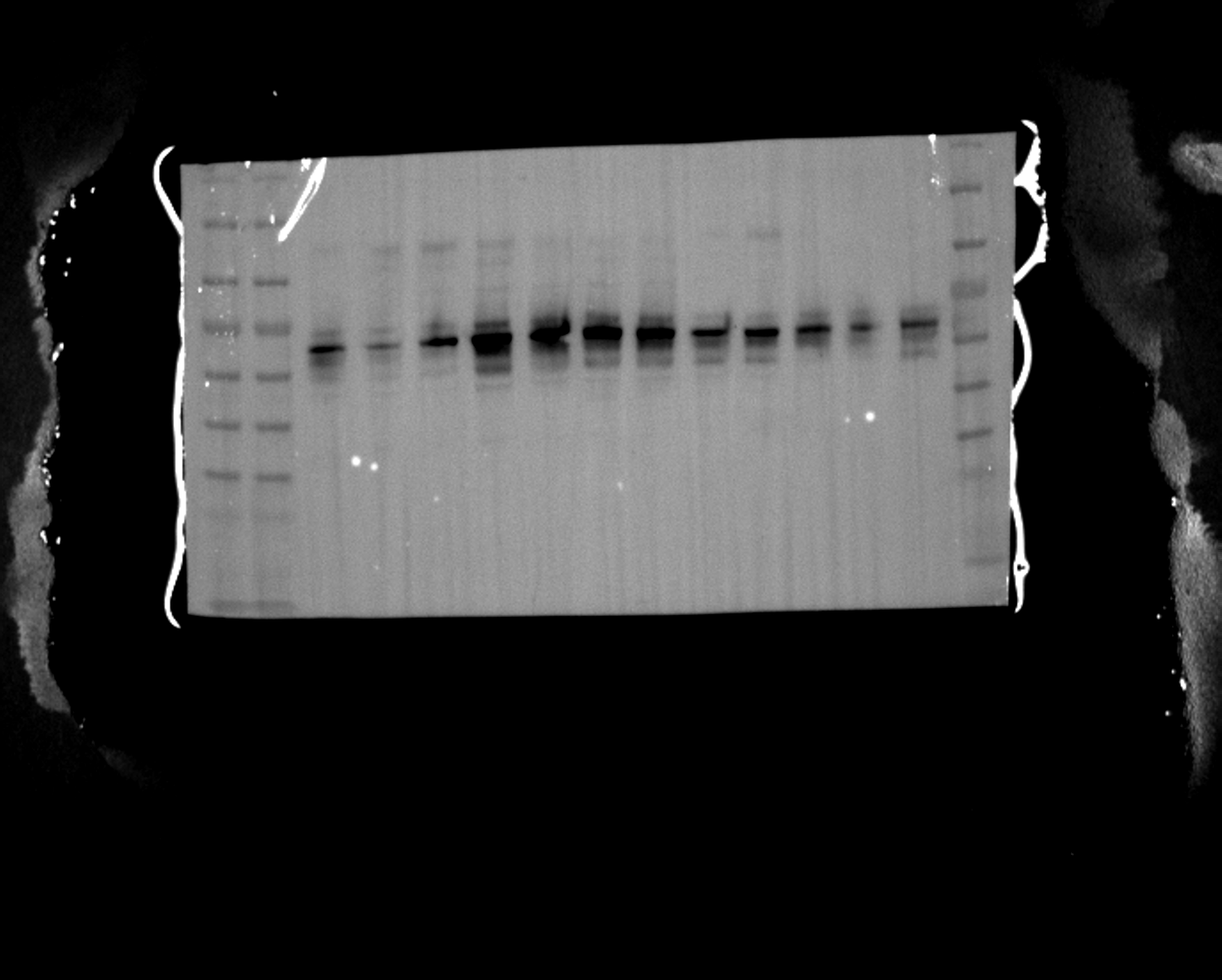

Supplement: Supplementary file 1 [file DataSheet1.zip › Supplementary Figure S5-24/Figure S8 FOXO4 .Tif]

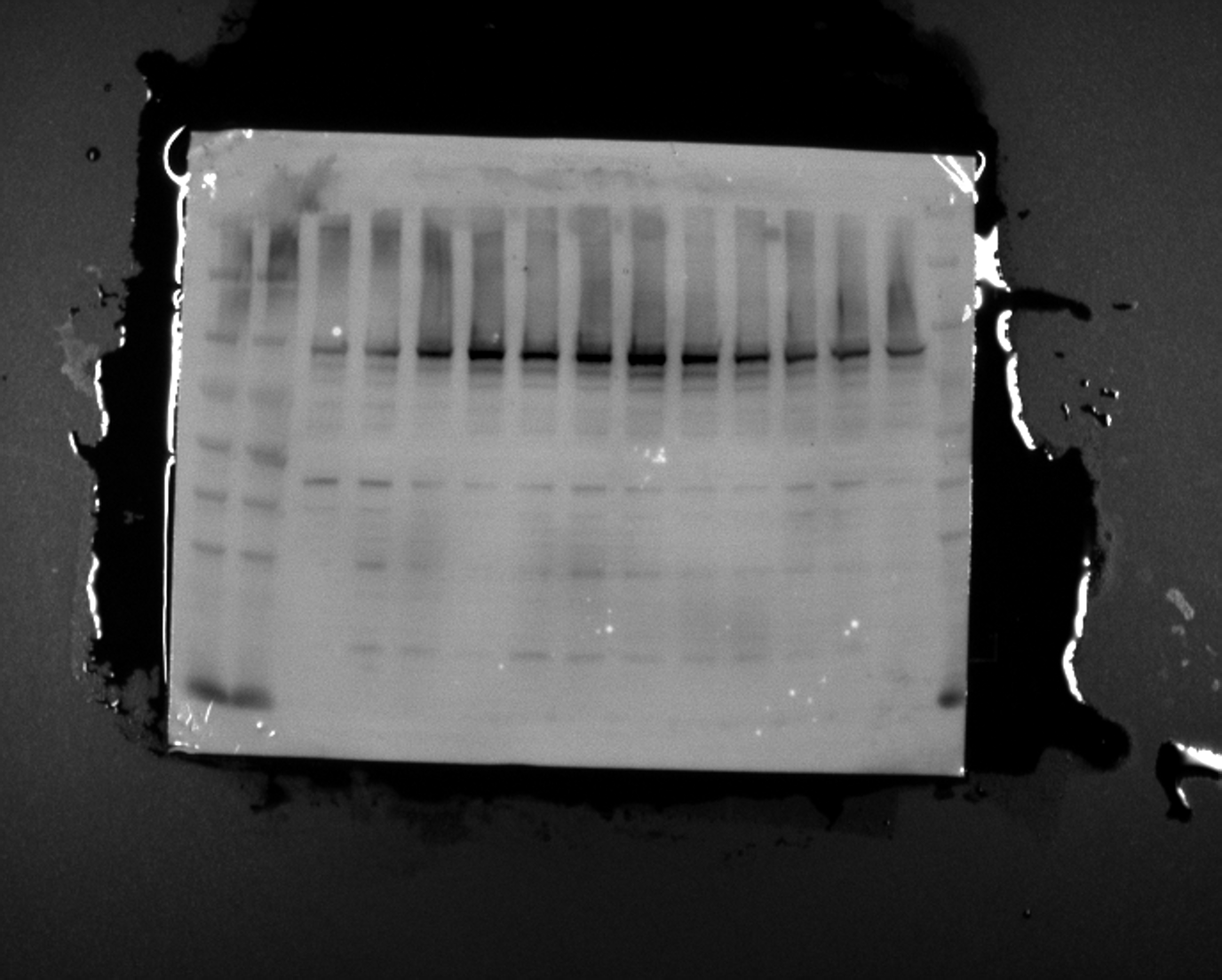

Supplement: Supplementary file 1 [file DataSheet1.zip › Supplementary Figure S5-24/Figure S7 STAT3 .Tif]

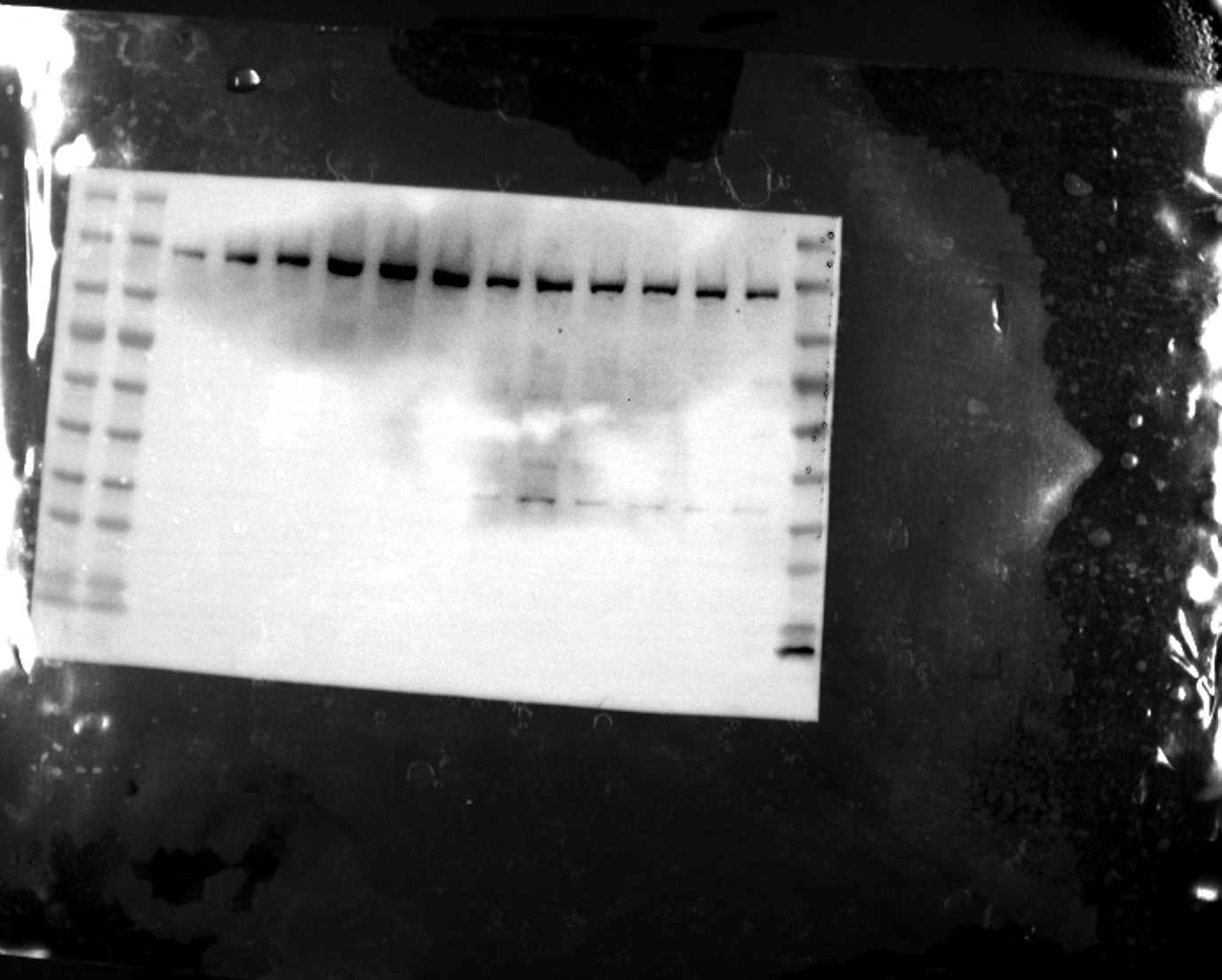

Supplement: Supplementary file 1 [file DataSheet1.zip › Supplementary Figure S5-24/Figure S6 JAK2 .Tif]

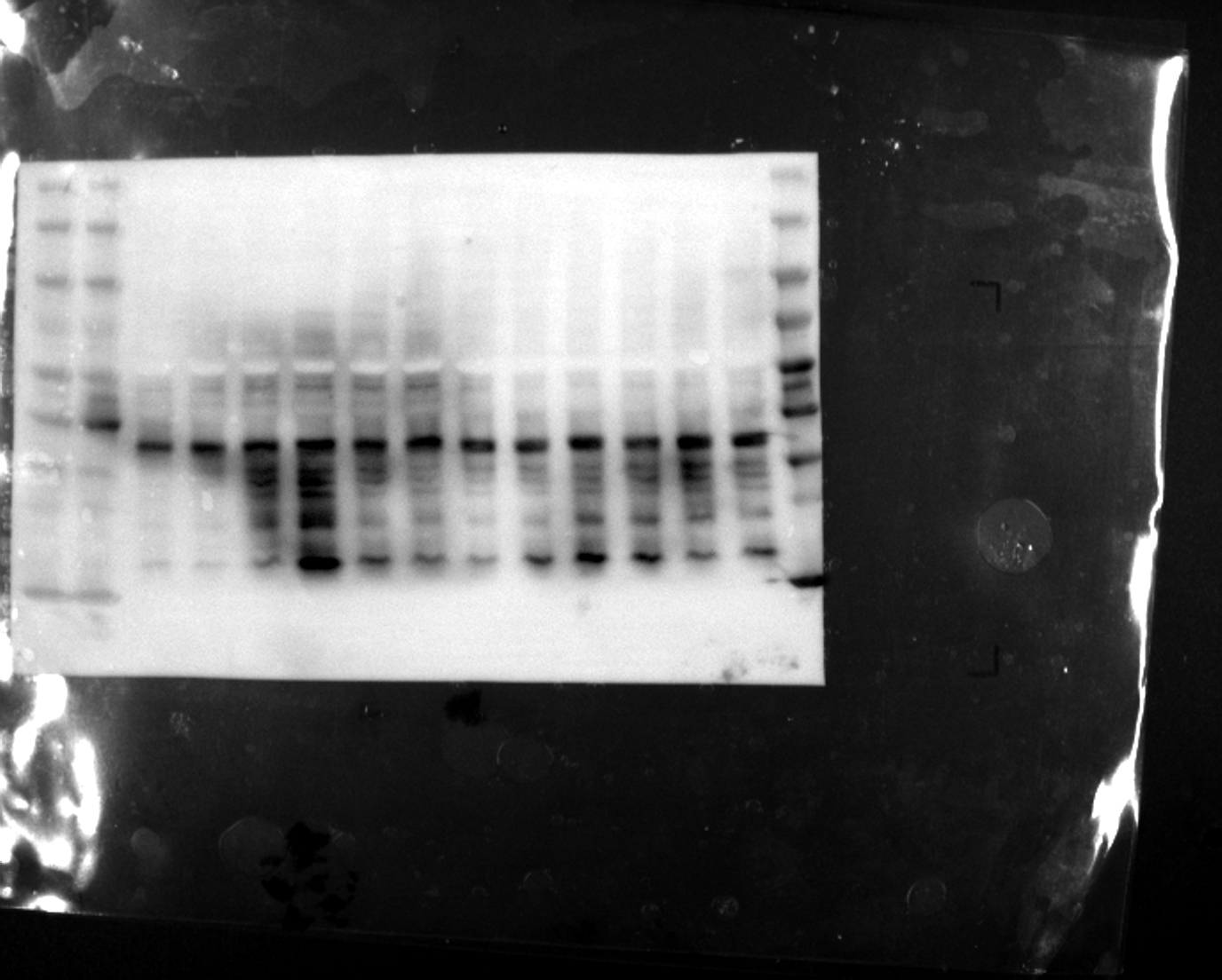

Supplement: Supplementary file 1 [file DataSheet1.zip › Supplementary Figure S5-24/Figure S9 actin.Tif]

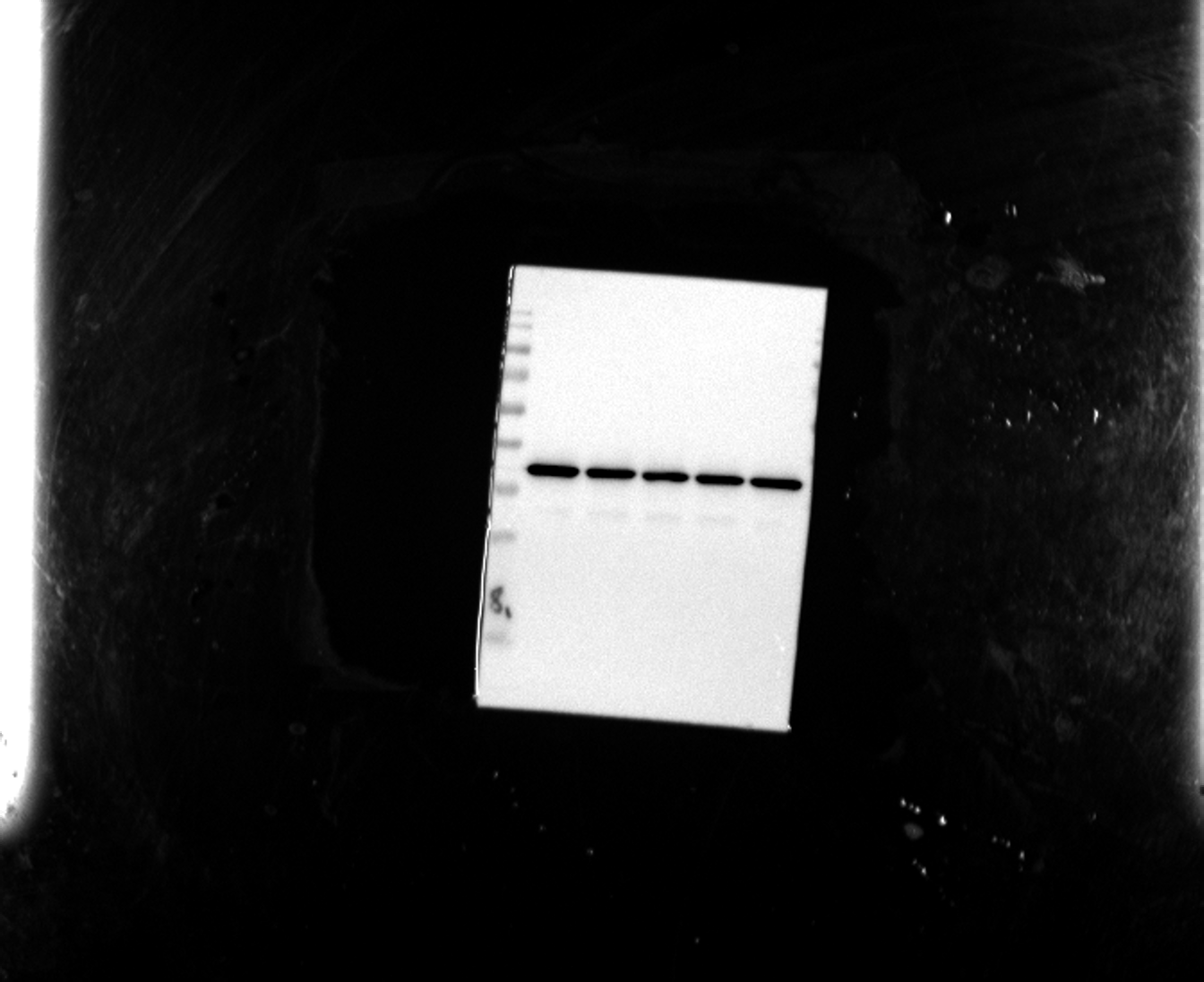

Supplement: Supplementary file 1 [file DataSheet1.zip › Supplementary Figure S5-24/Figure S17 GAPDH.Tif]

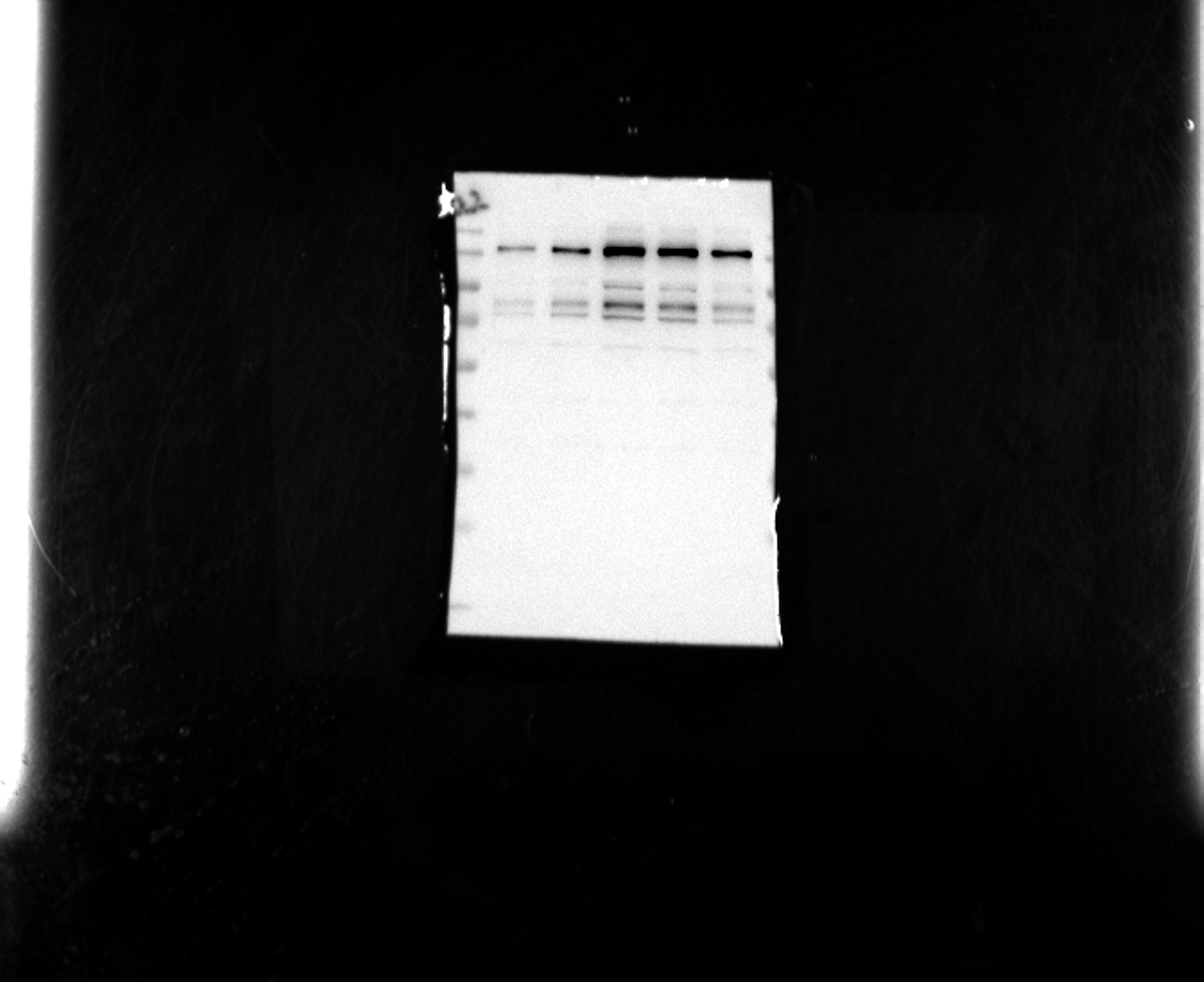

Supplement: Supplementary file 1 [file DataSheet1.zip › Supplementary Figure S5-24/Figure S14 JAK2.Tif]

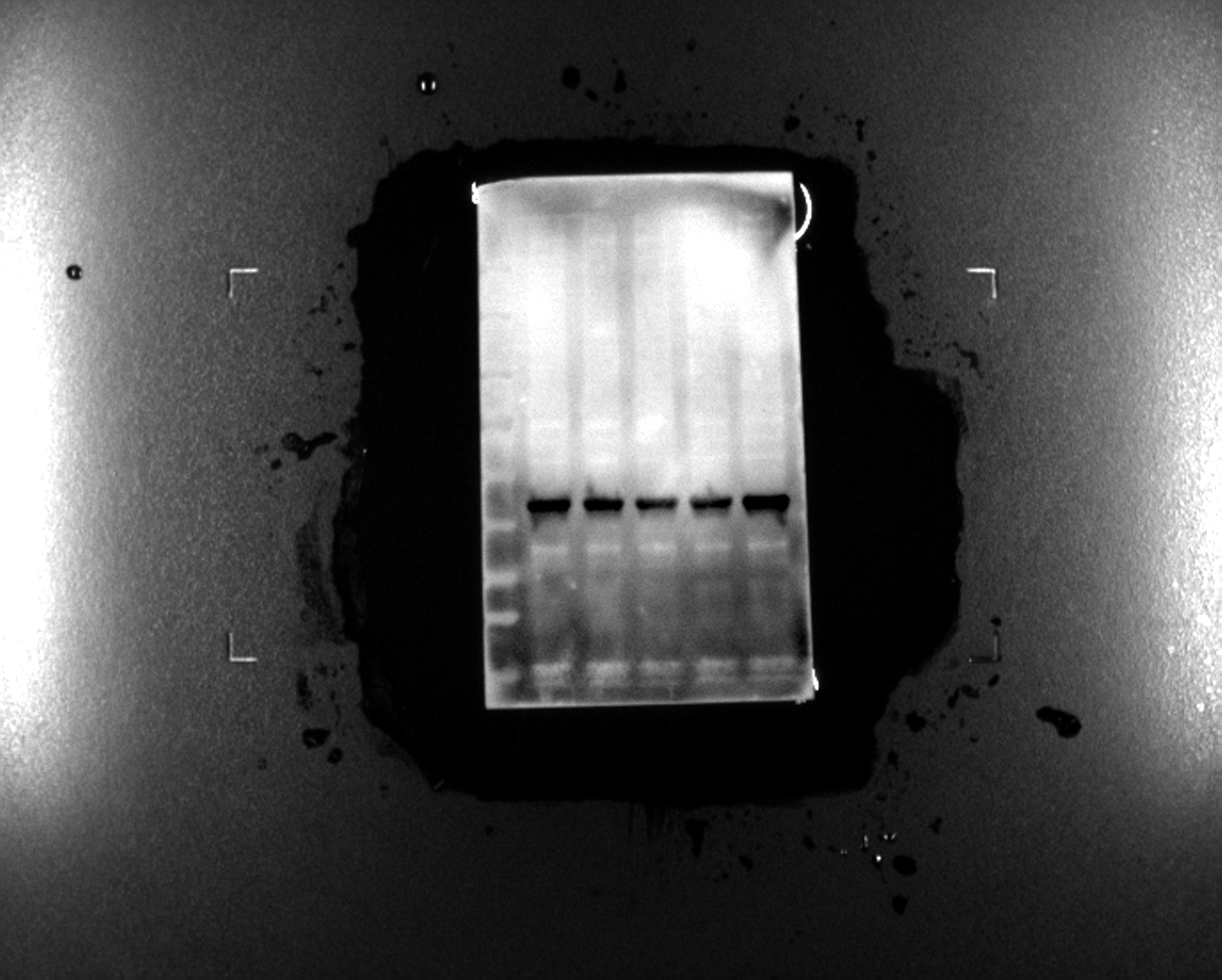

Supplement: Supplementary file 1 [file DataSheet1.zip › Supplementary Figure S5-24/Figure S18 GLUT4.Tif]

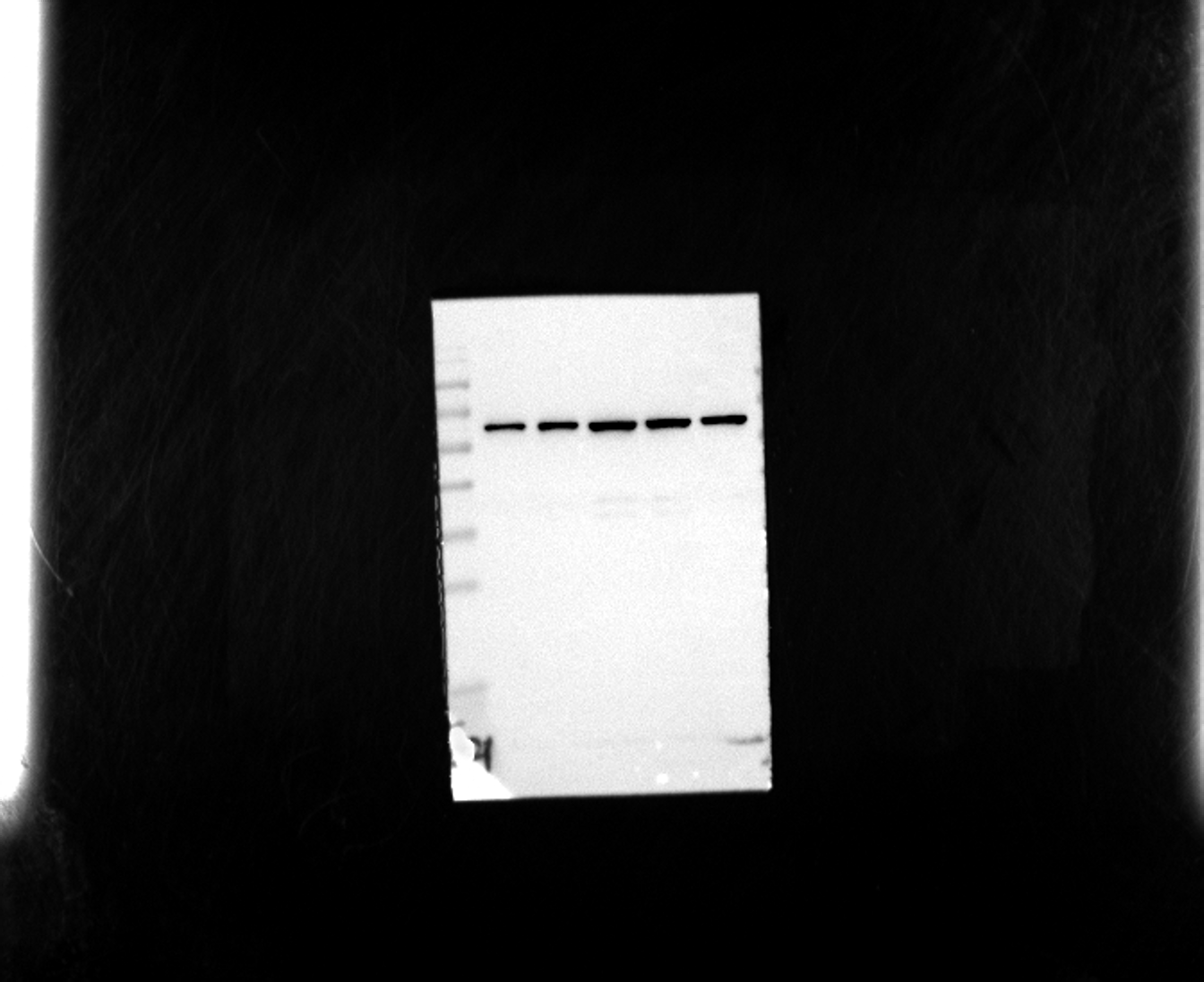

Supplement: Supplementary file 1 [file DataSheet1.zip › Supplementary Figure S5-24/Figure S16 FOXO4.Tif]

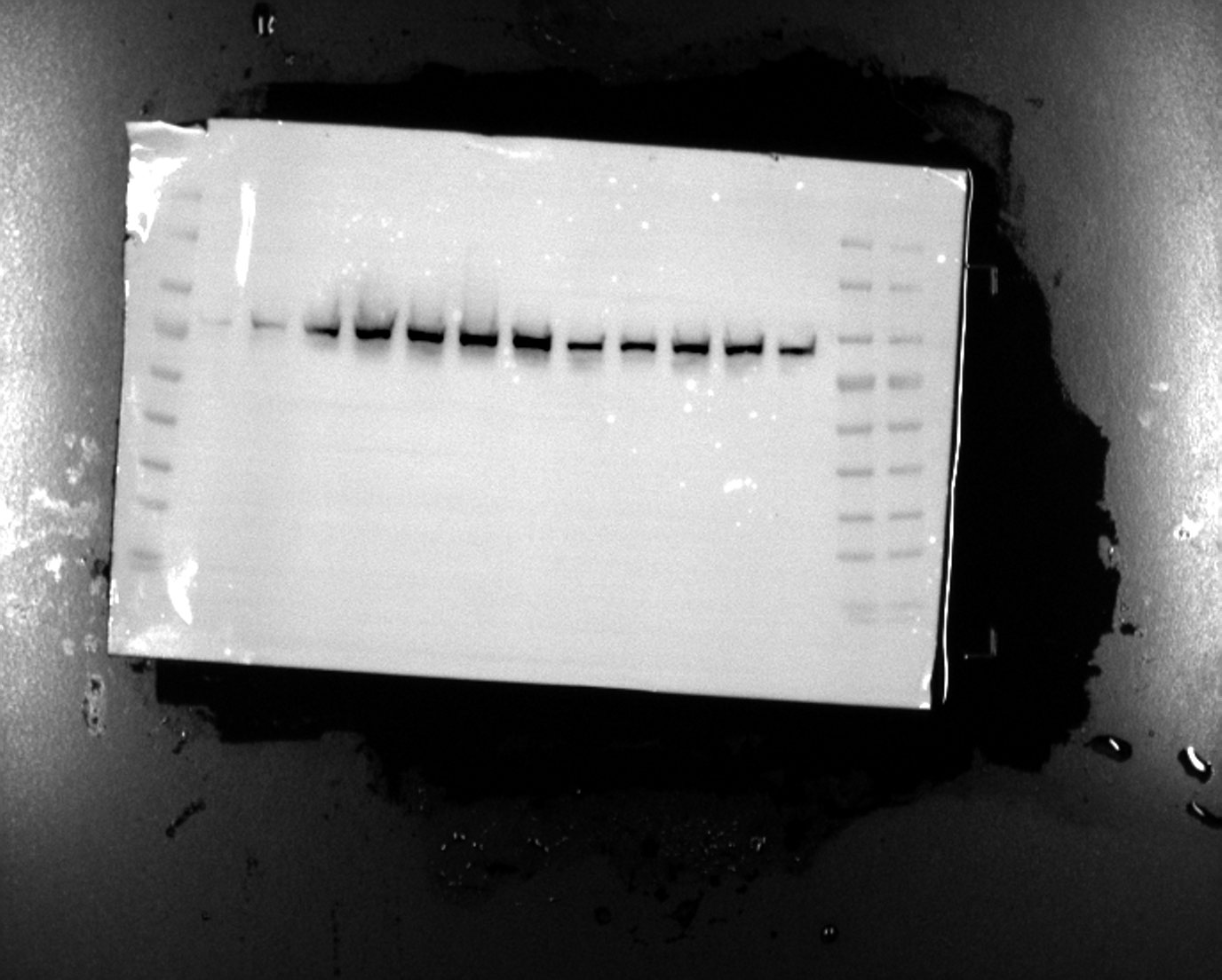

Supplement: Supplementary file 1 [file DataSheet1.zip › Supplementary Figure S5-24/Figure S10 p-STAT3.Tif]

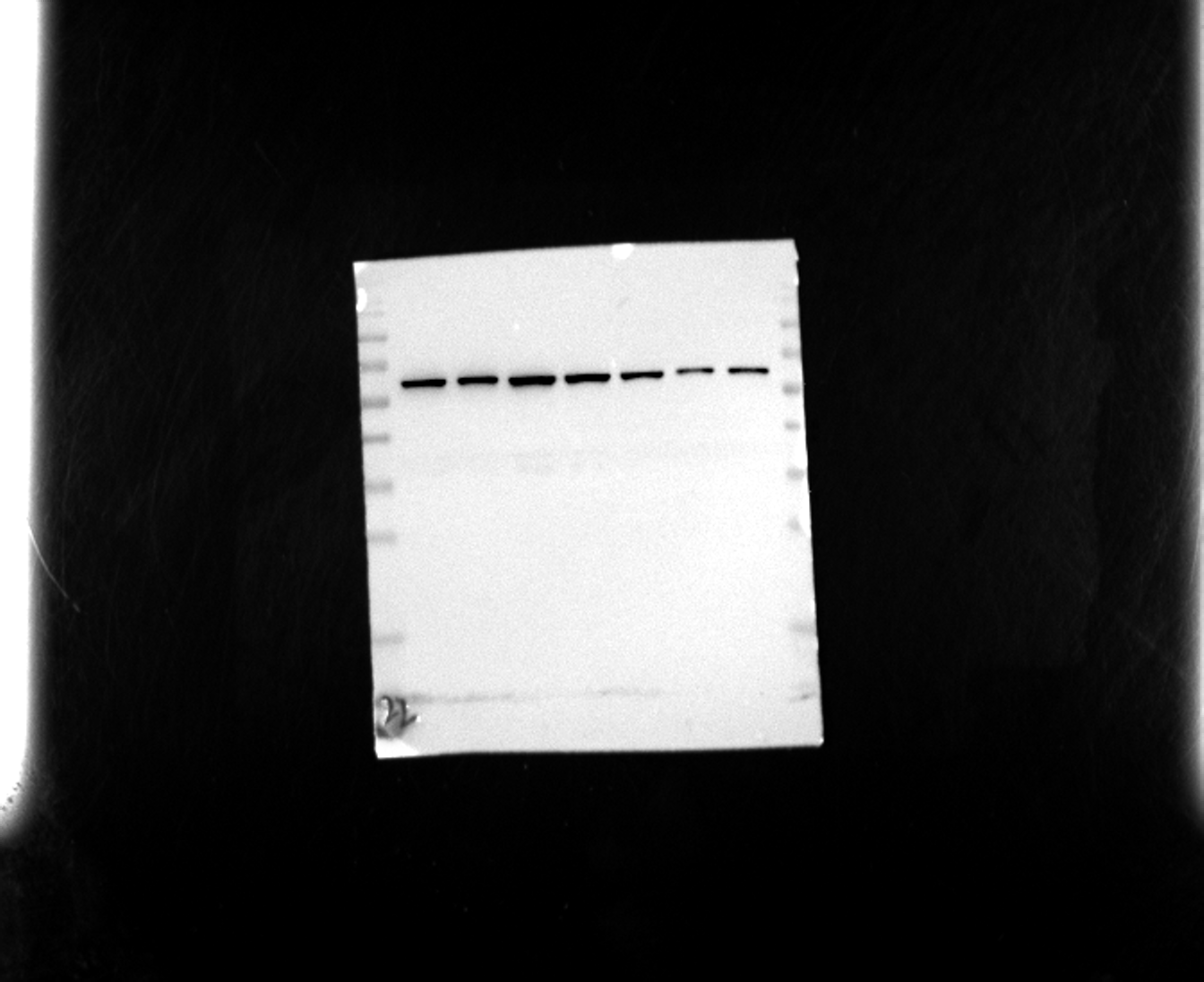

Supplement: Supplementary file 1 [file DataSheet1.zip › Supplementary Figure S5-24/Figure S23 FOXO4.Tif]

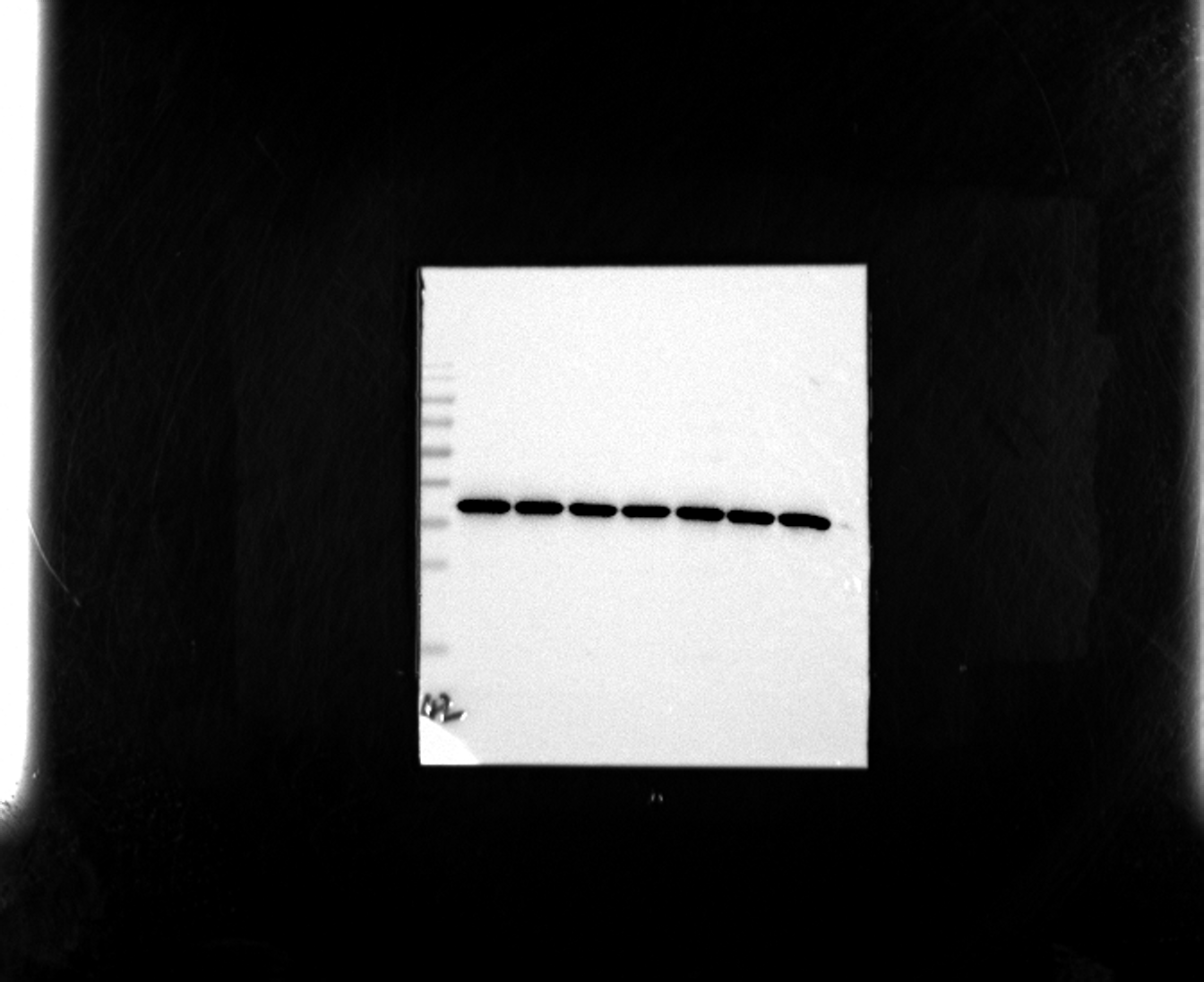

Supplement: Supplementary file 1 [file DataSheet1.zip › Supplementary Figure S5-24/Figure S24 GAPDH.Tif]

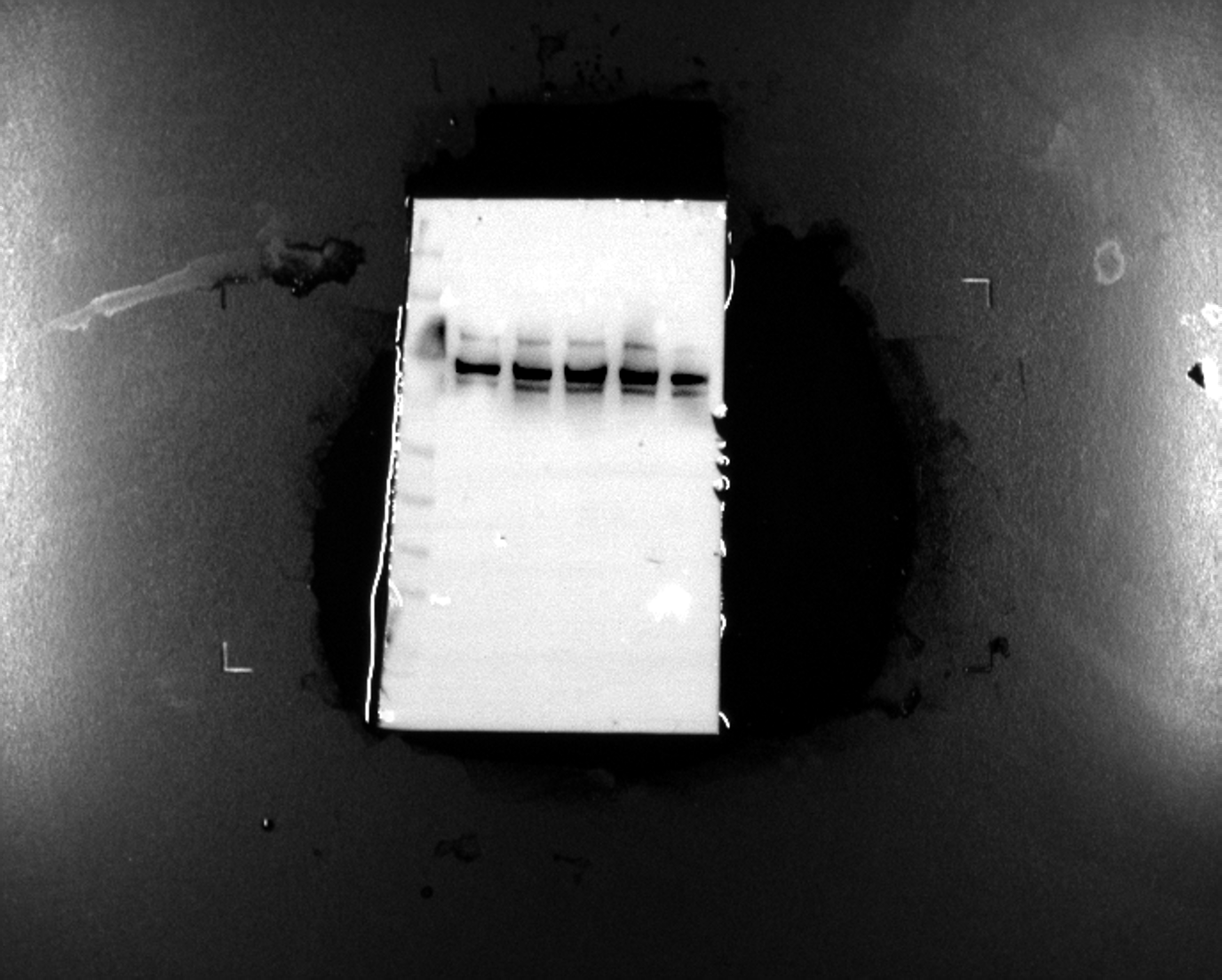

Supplement: Supplementary file 1 [file DataSheet1.zip › Supplementary Figure S5-24/Figure S19 p-STAT3.Tif]

|  | Control | PCOS | CDD-L | CDD-H |
| --- | --- | --- | --- | --- |
| 1 | 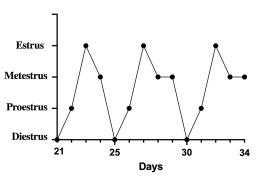 | 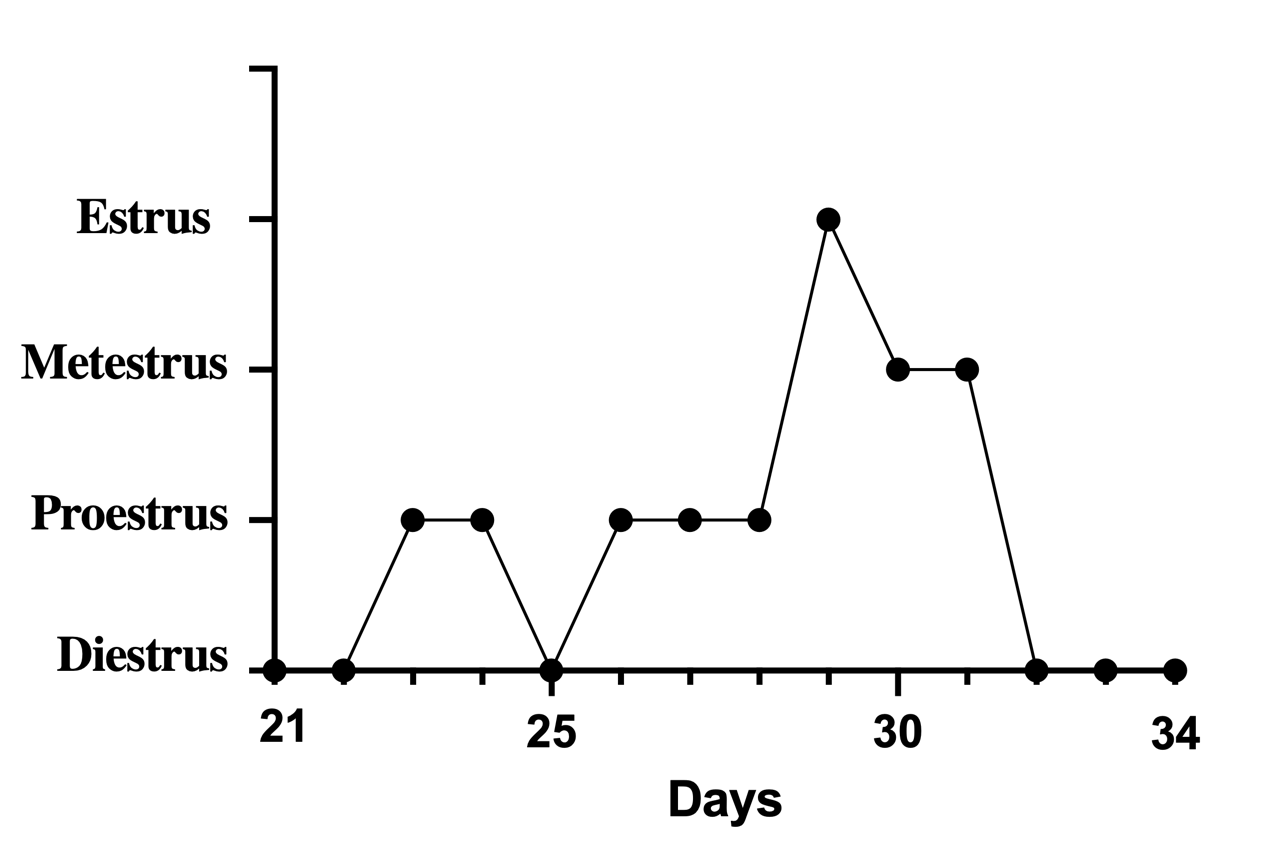 | 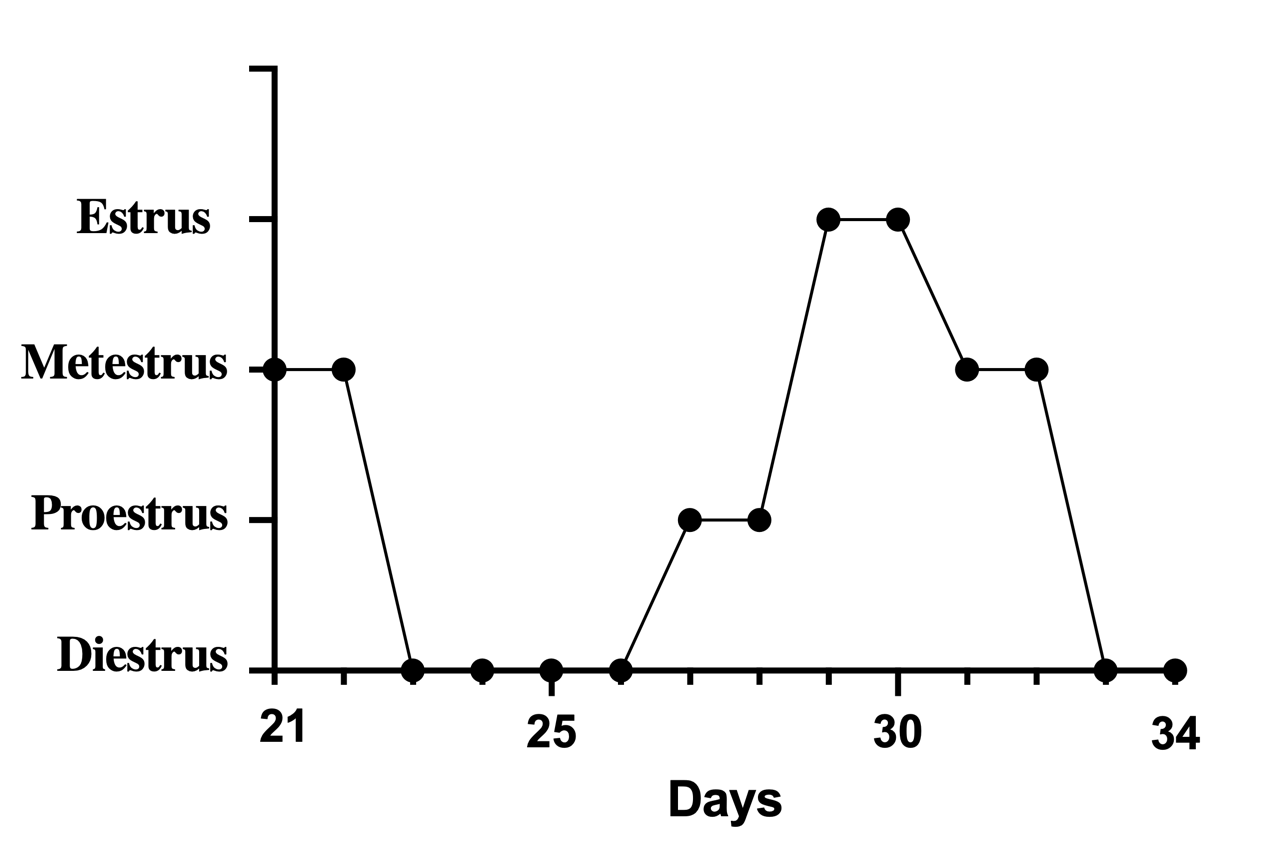 | 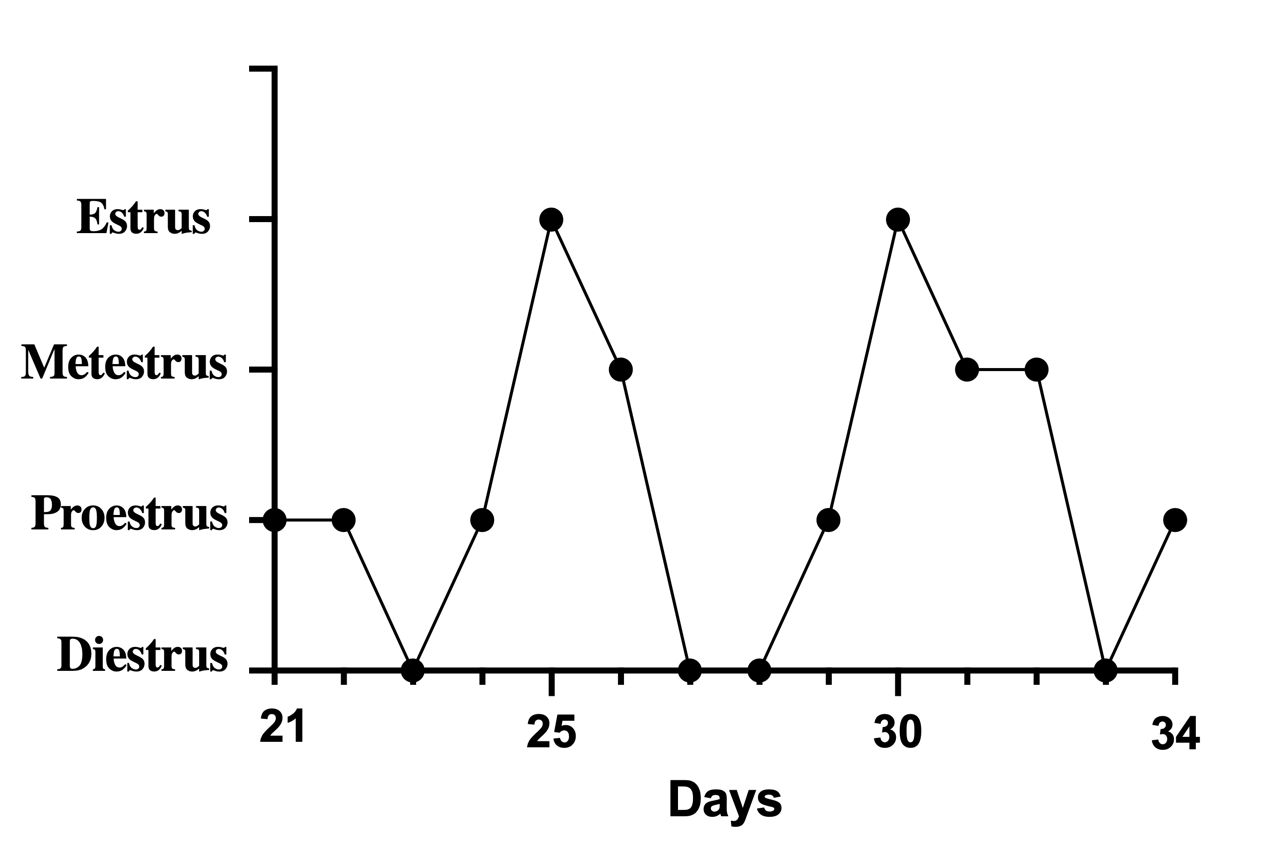 |
| 2 | 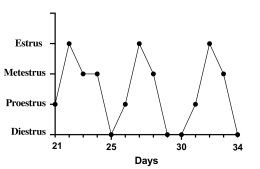 | 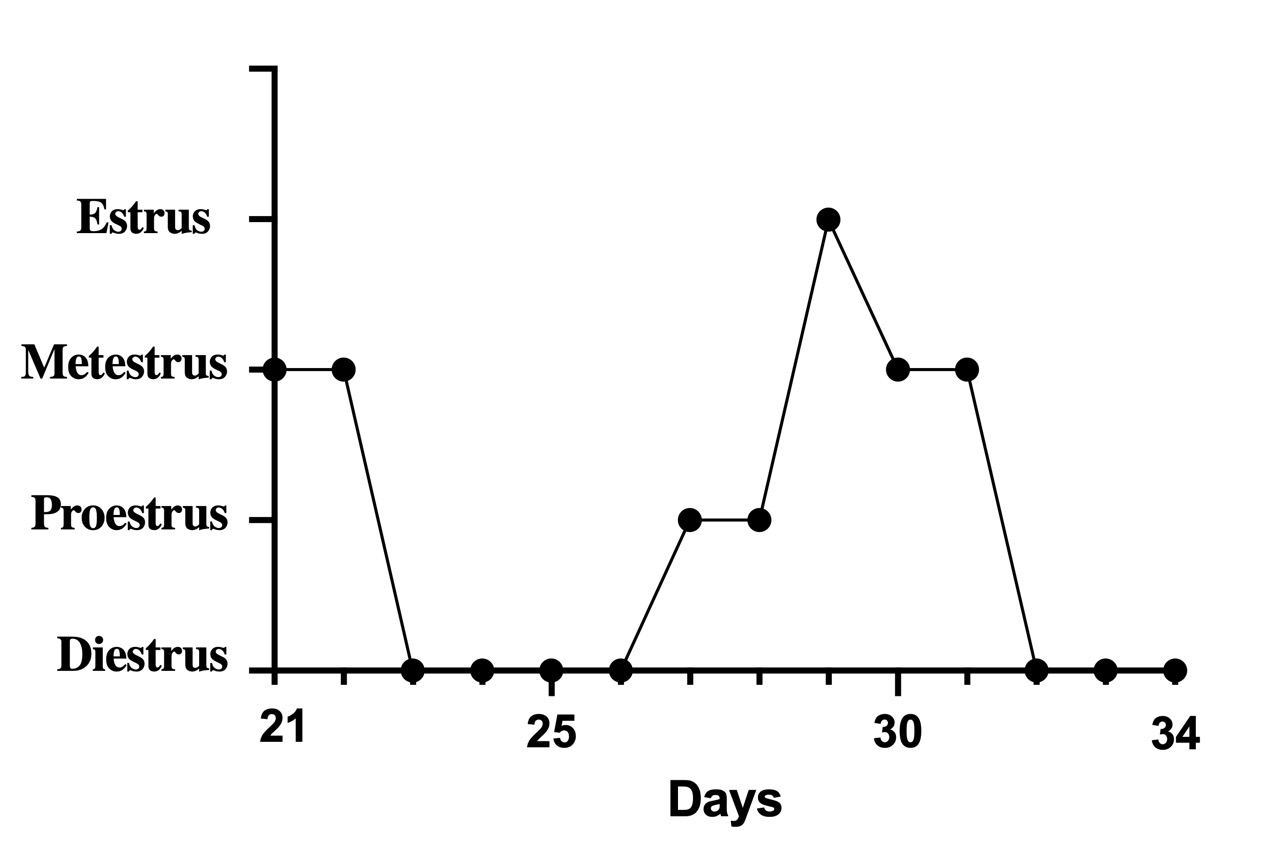 | 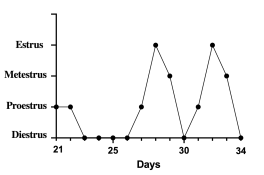 | 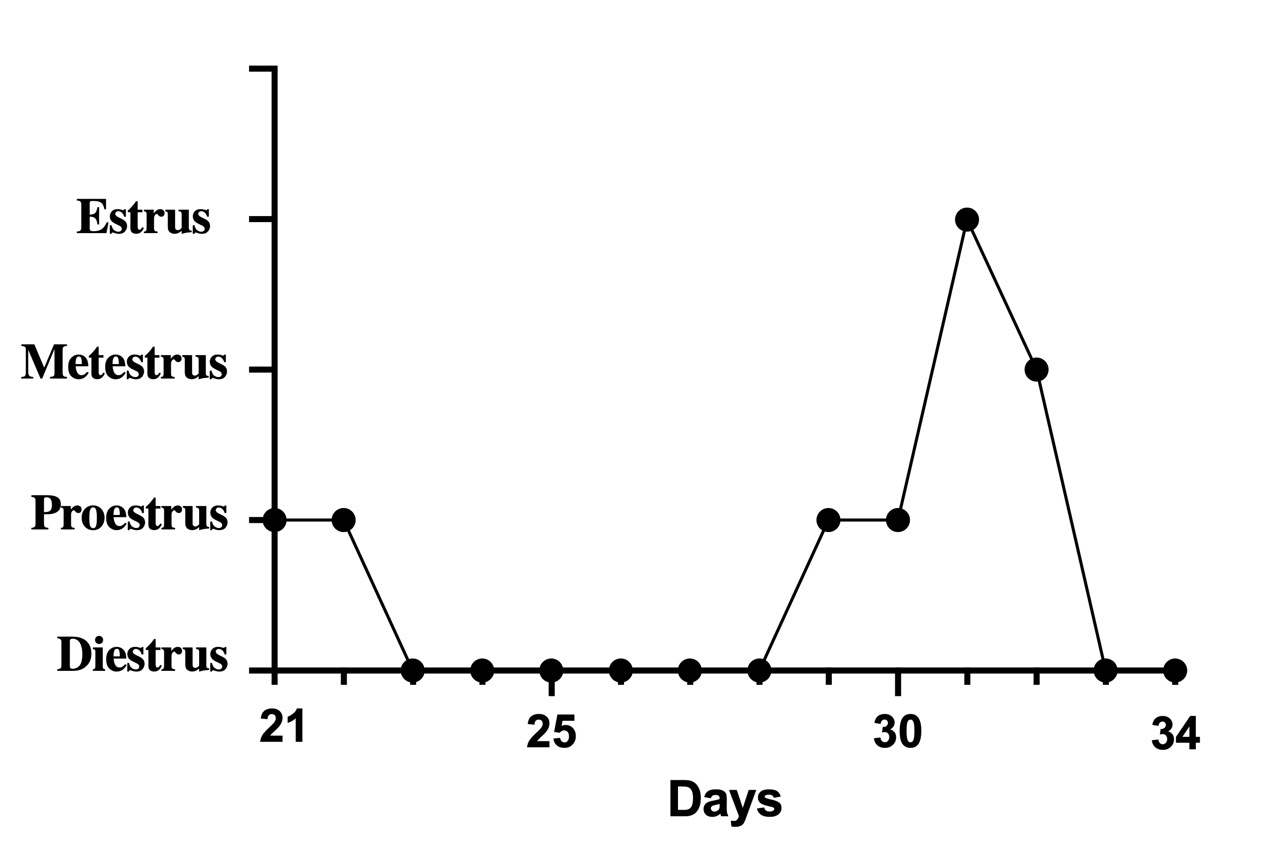 |
| 3 | 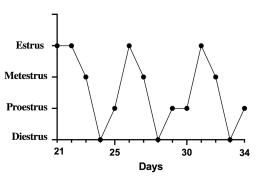 | 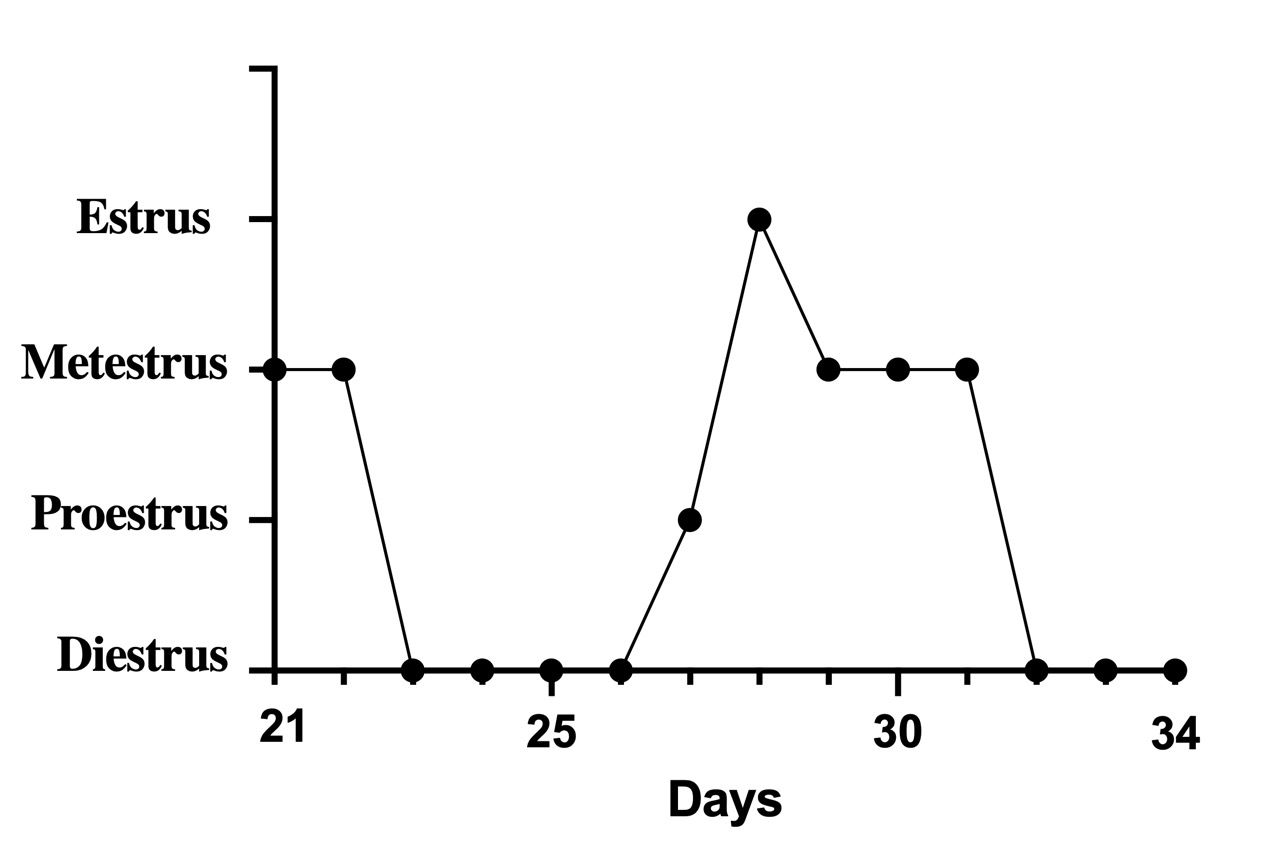 | 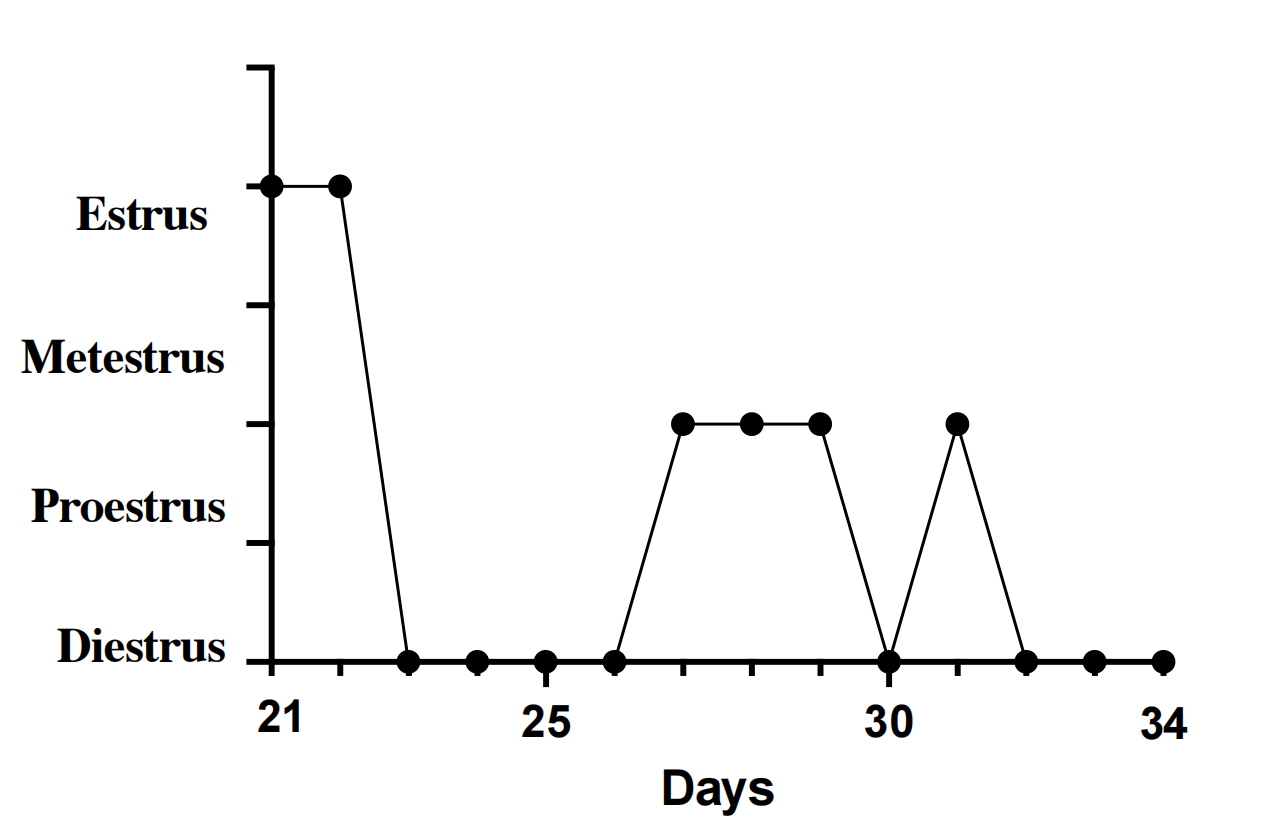 | 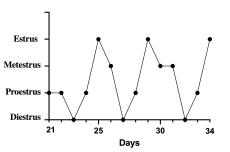 |
| 4 | 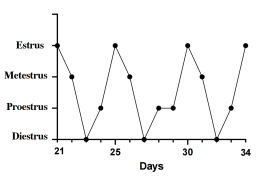 | 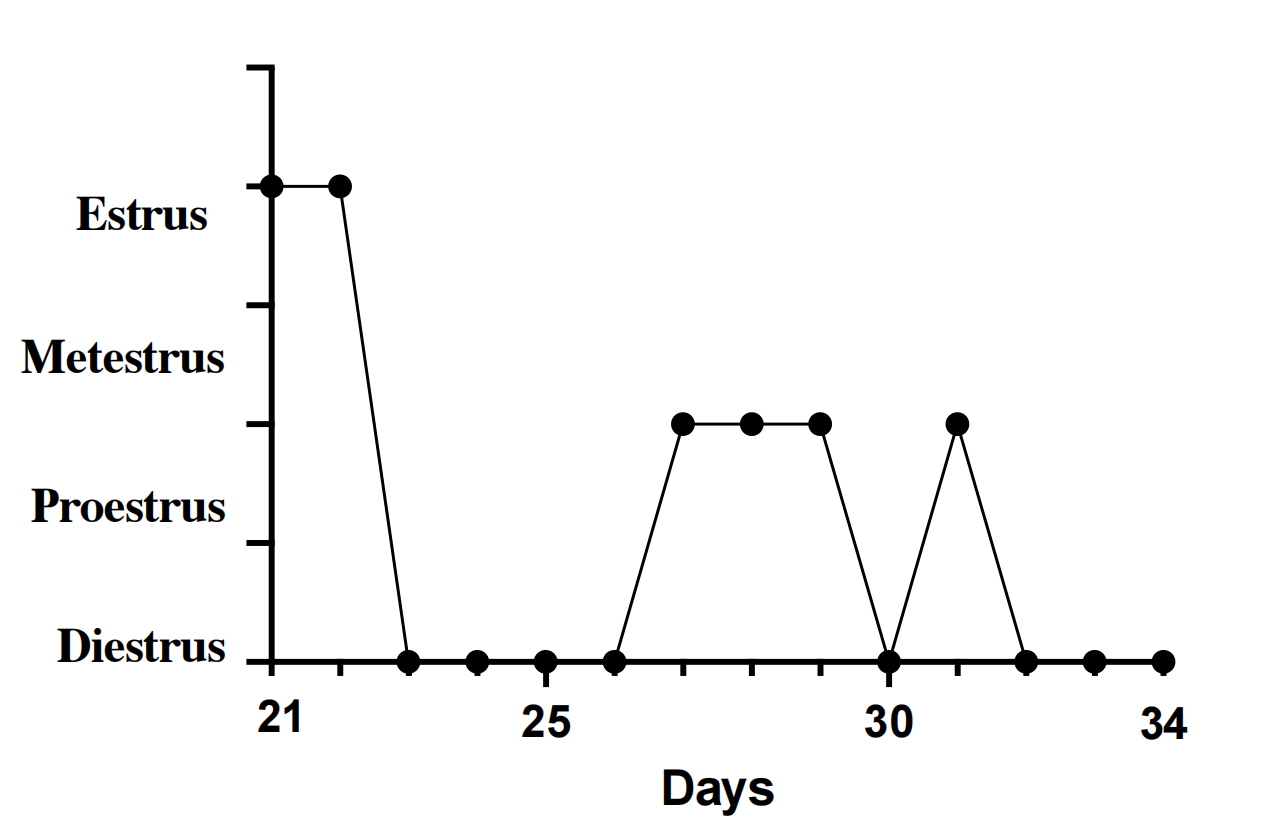 | 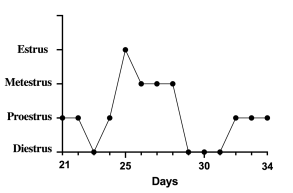 | 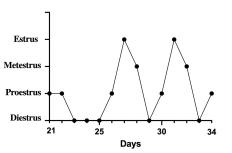 |
| 5 | 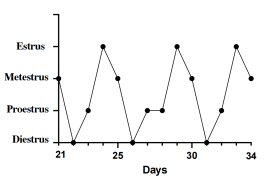 | 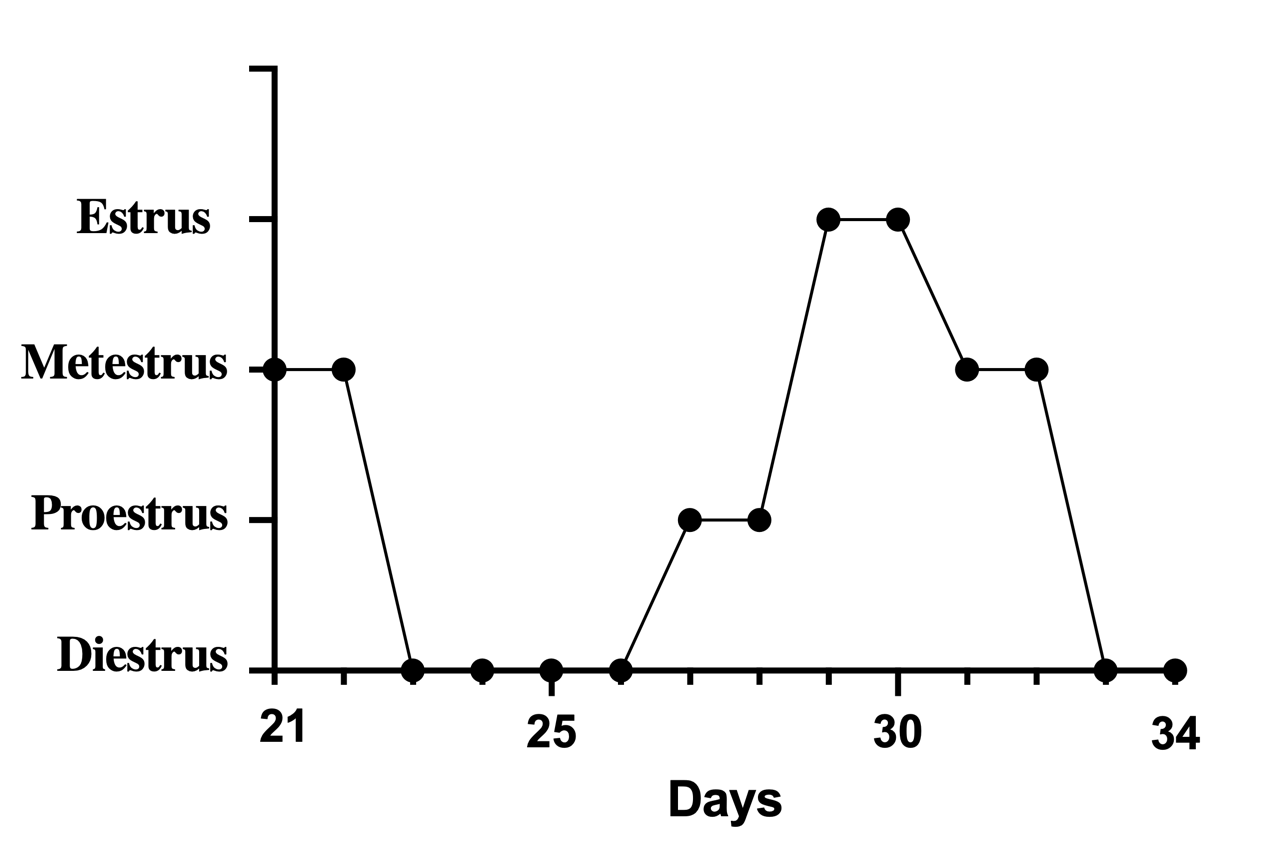 | 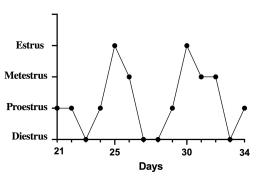 | 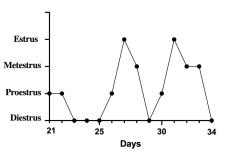 |
| 6 | 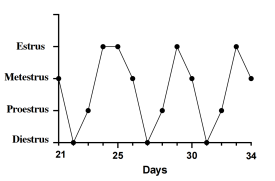 | 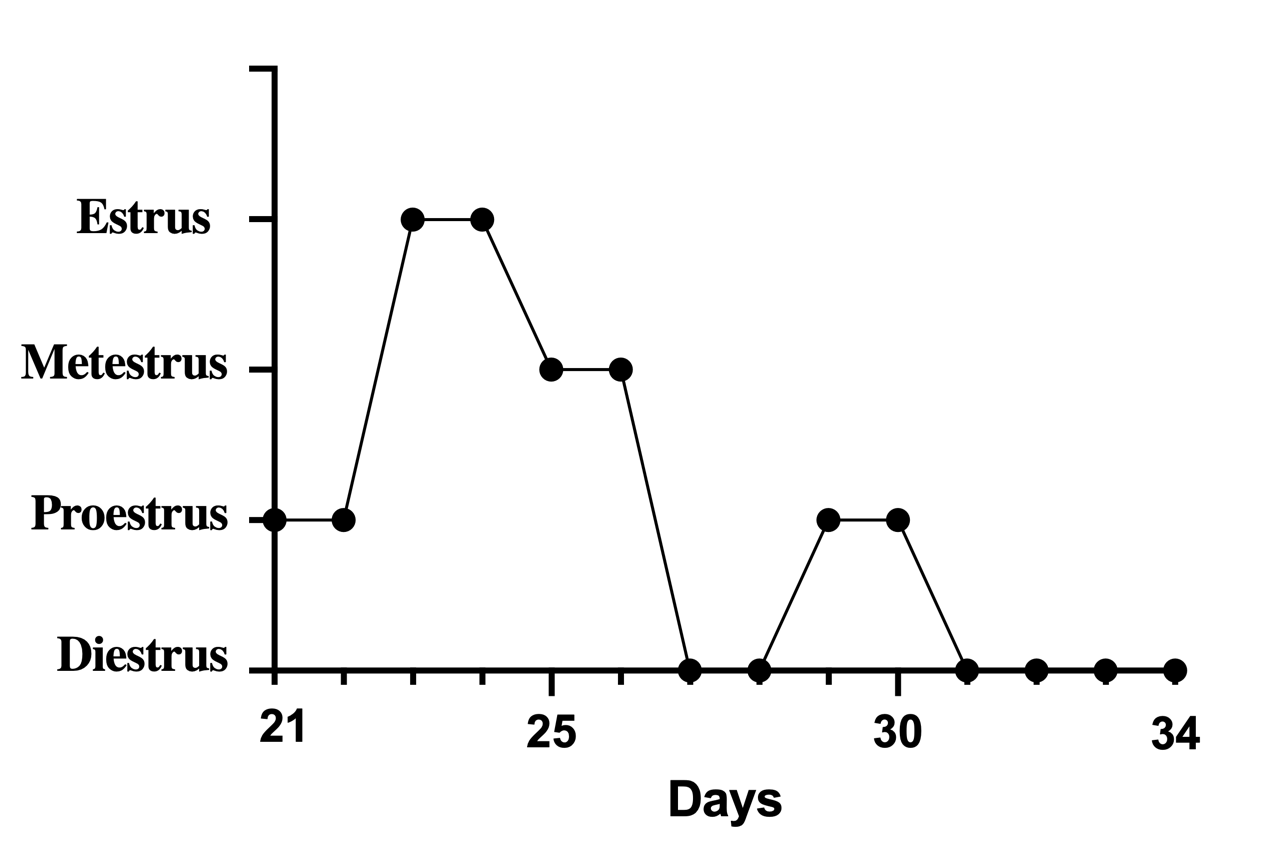 | 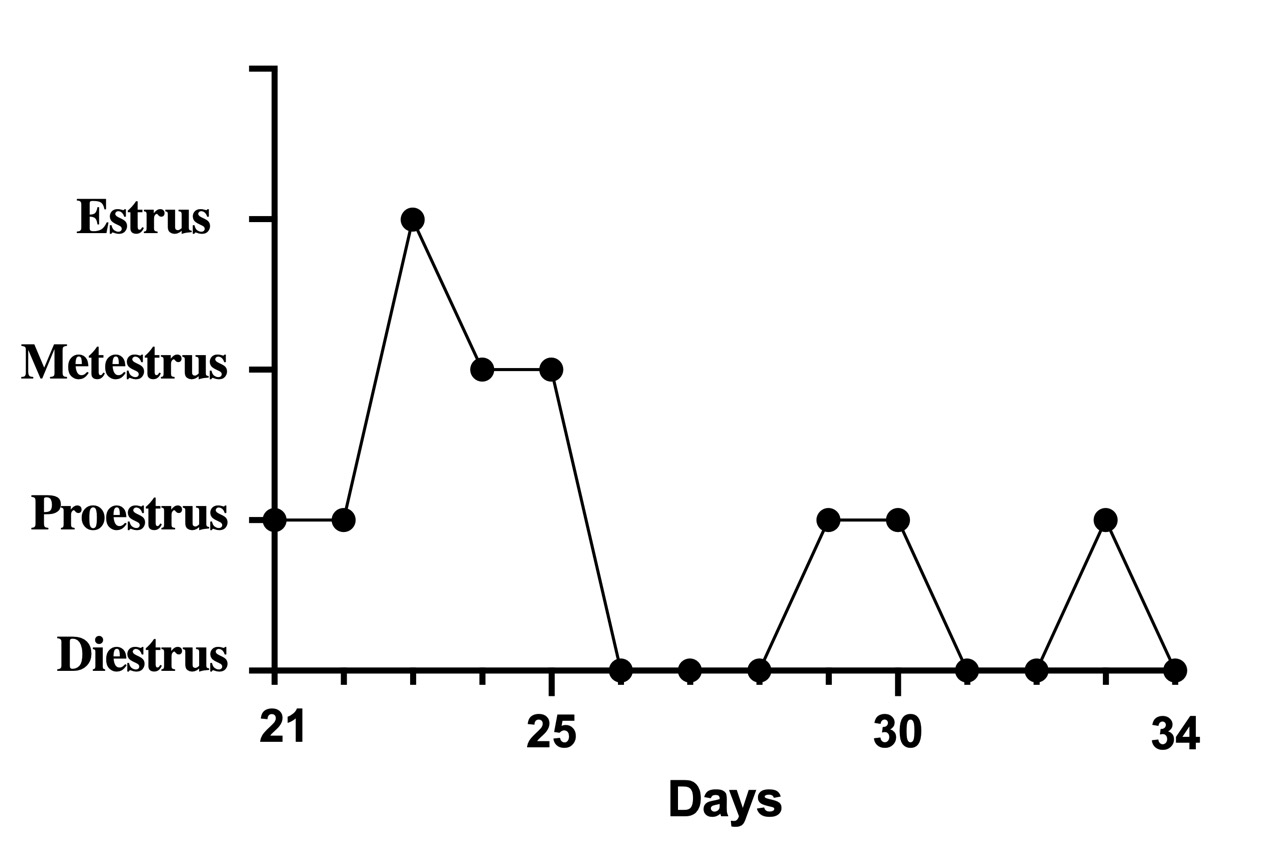 | 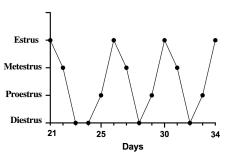 |
| 7 | 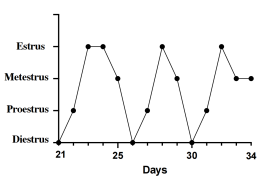 | 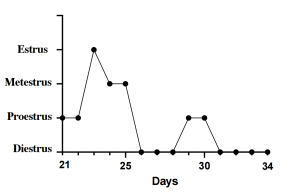 | 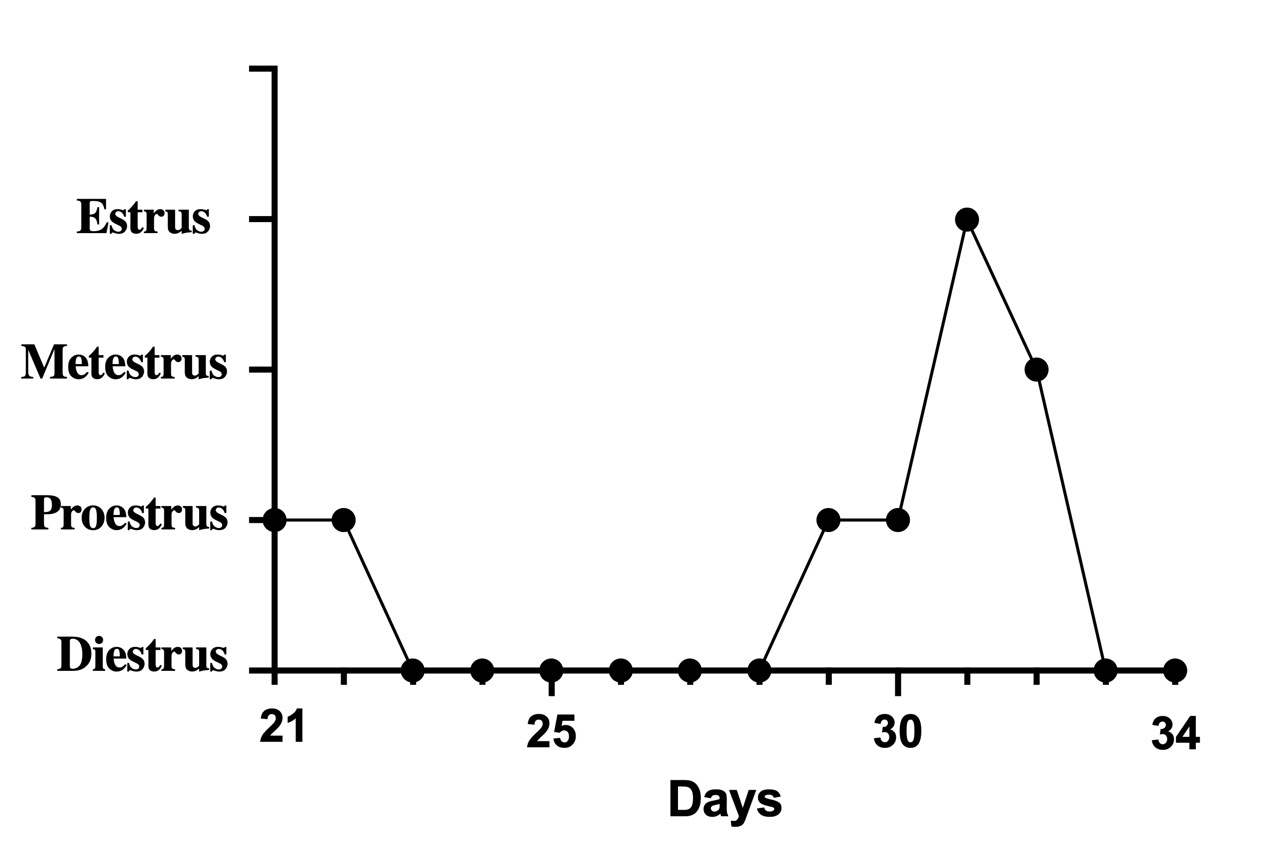 | 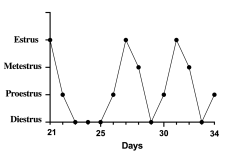 |
| 8 | 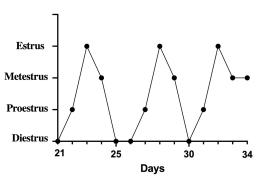 | 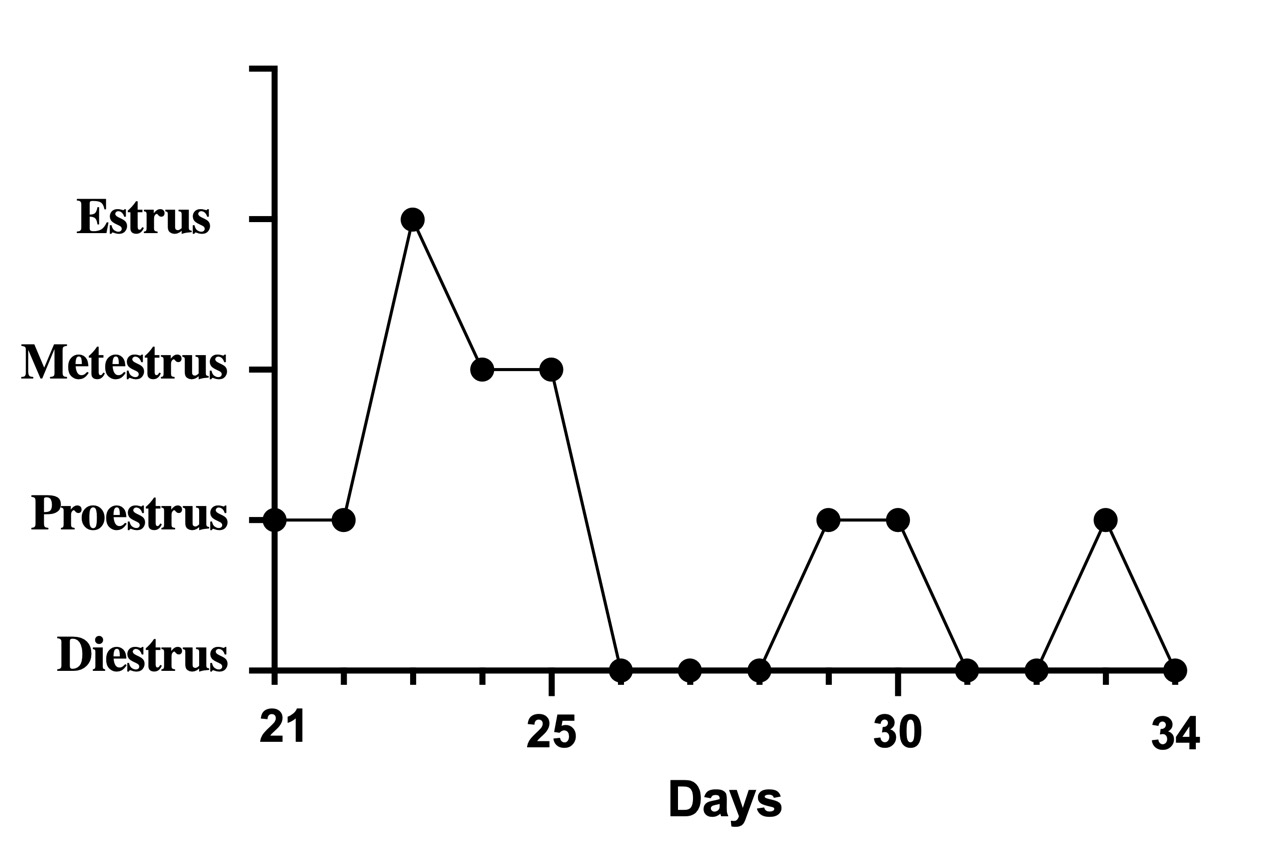 | 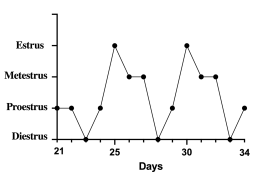 | 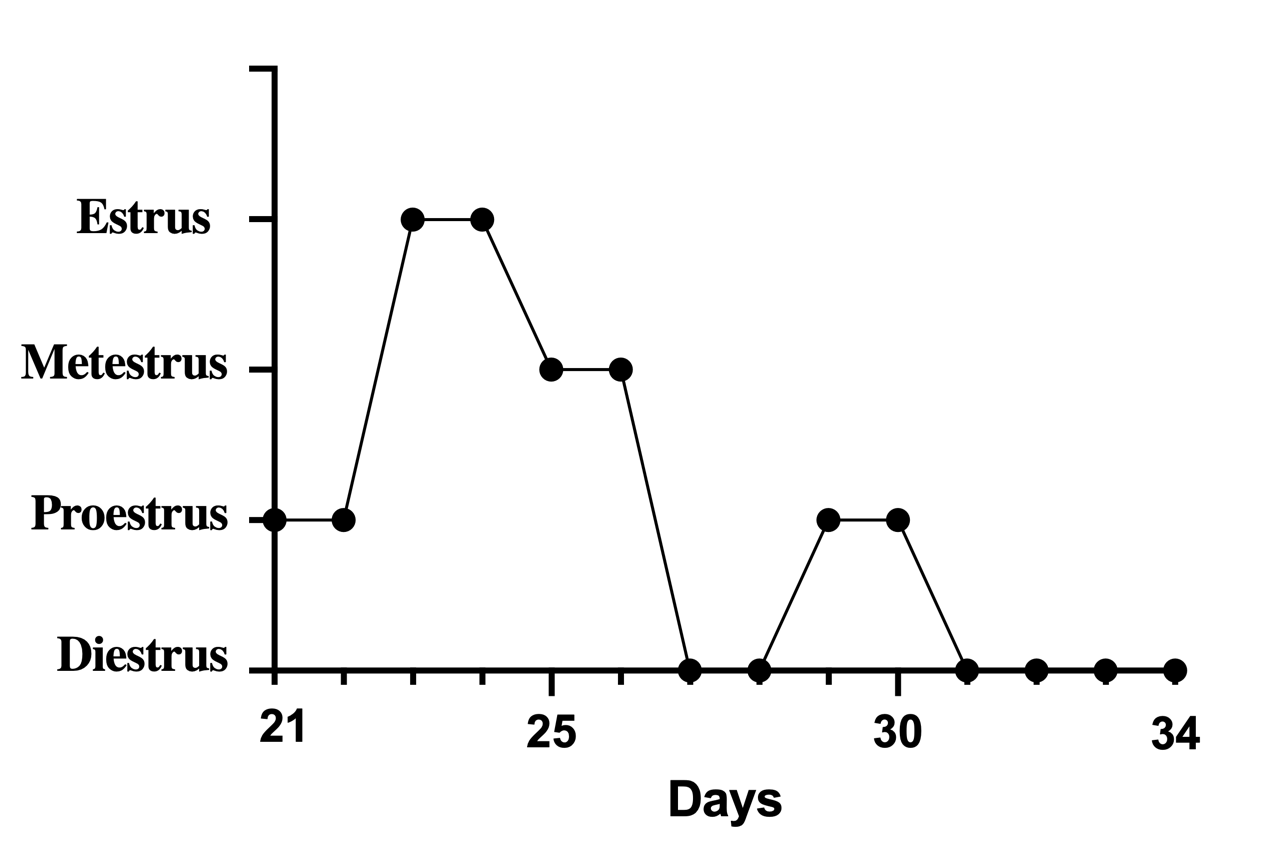 |
| 9 | 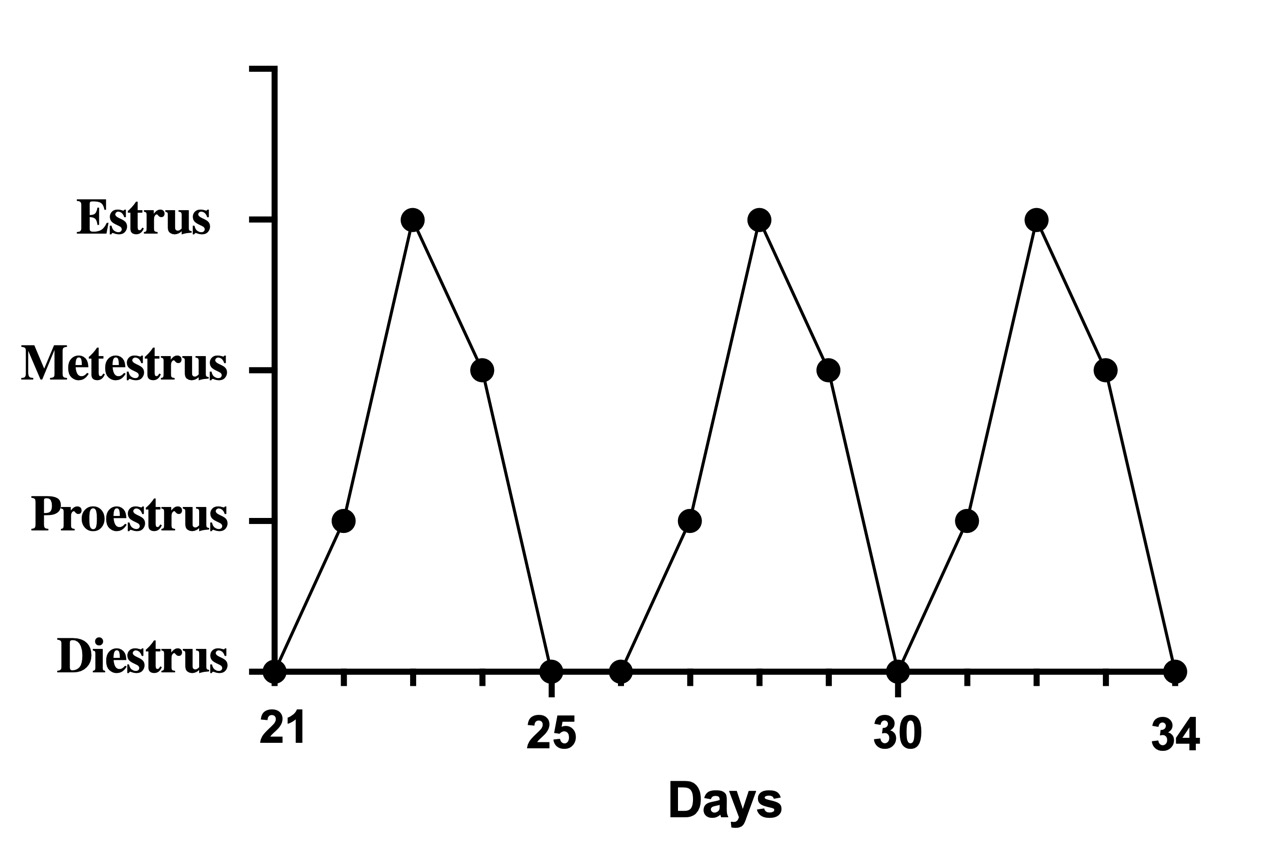 | 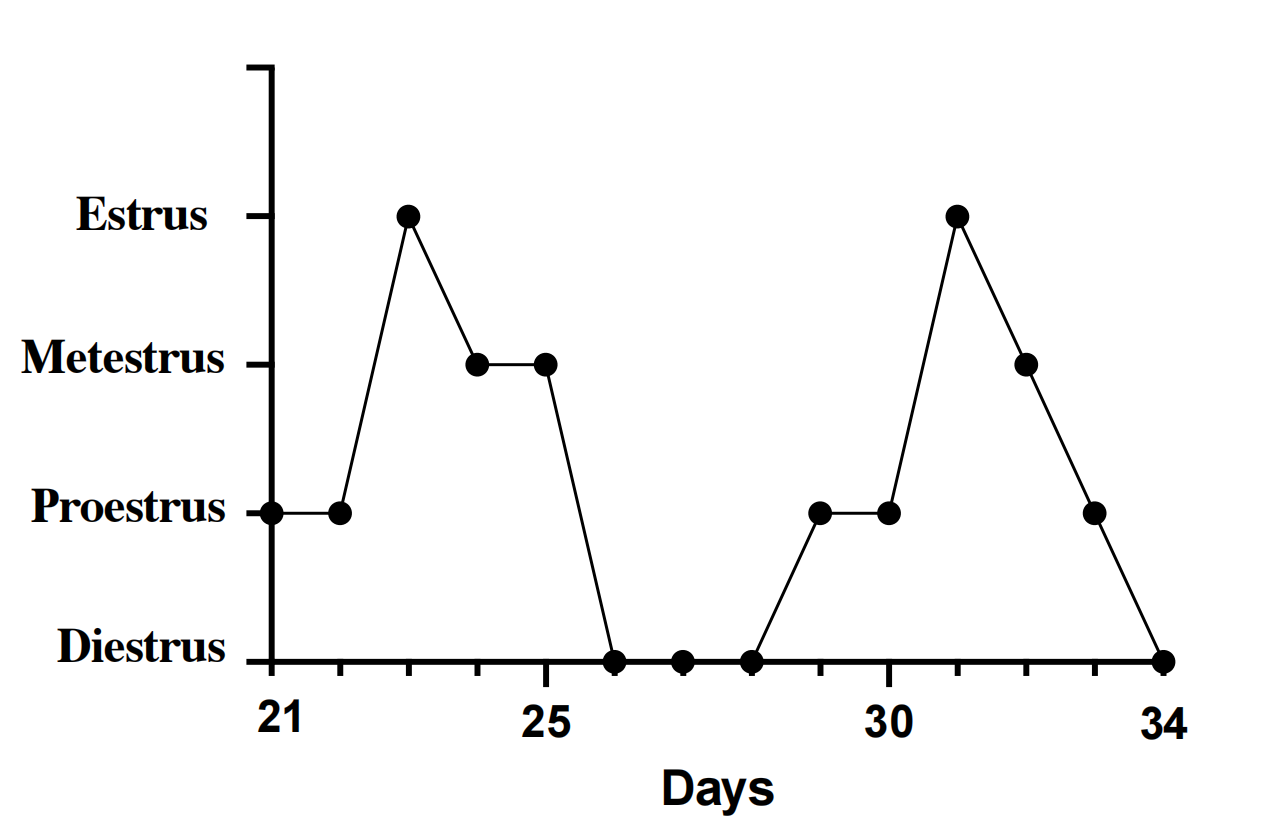 | 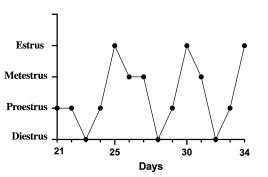 | 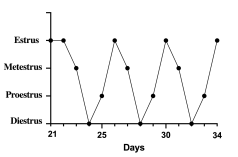 |
| 10 | 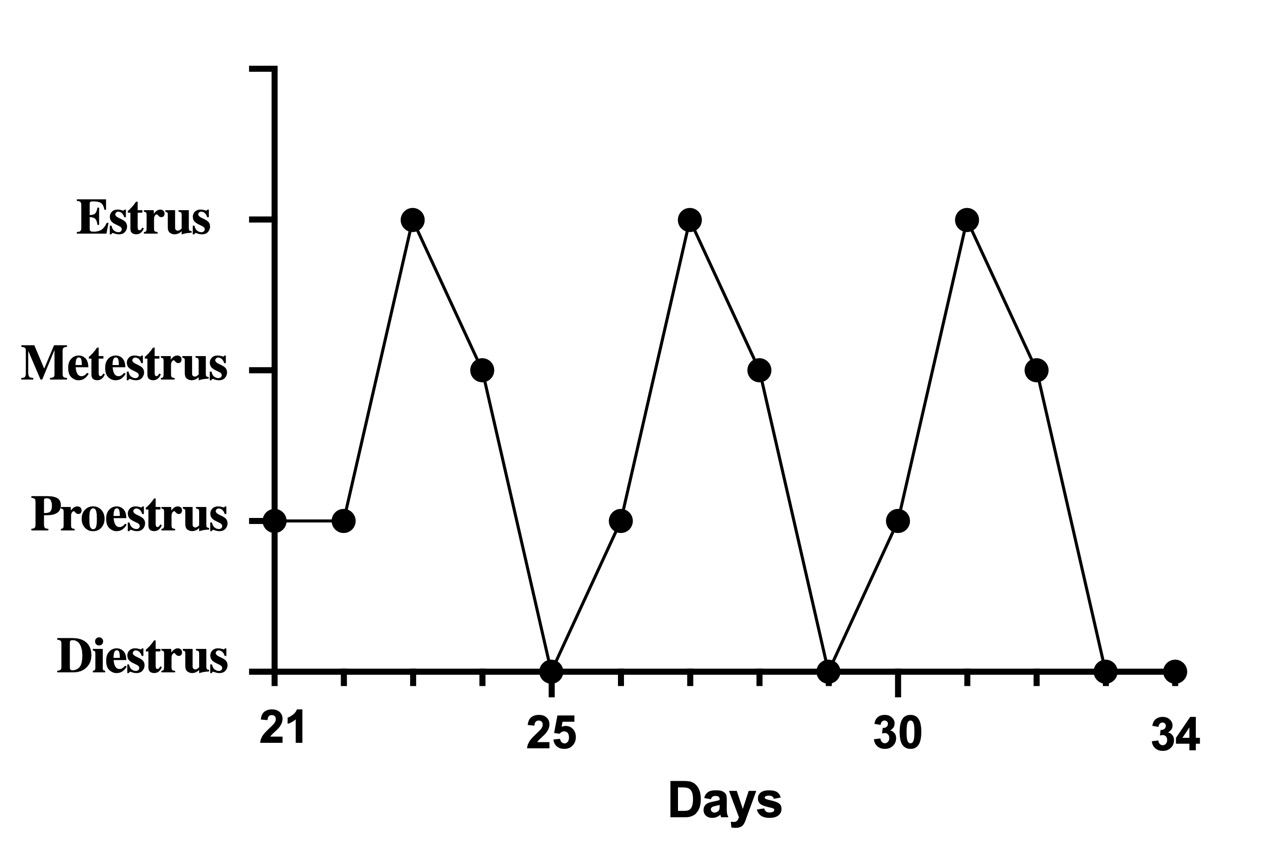 | 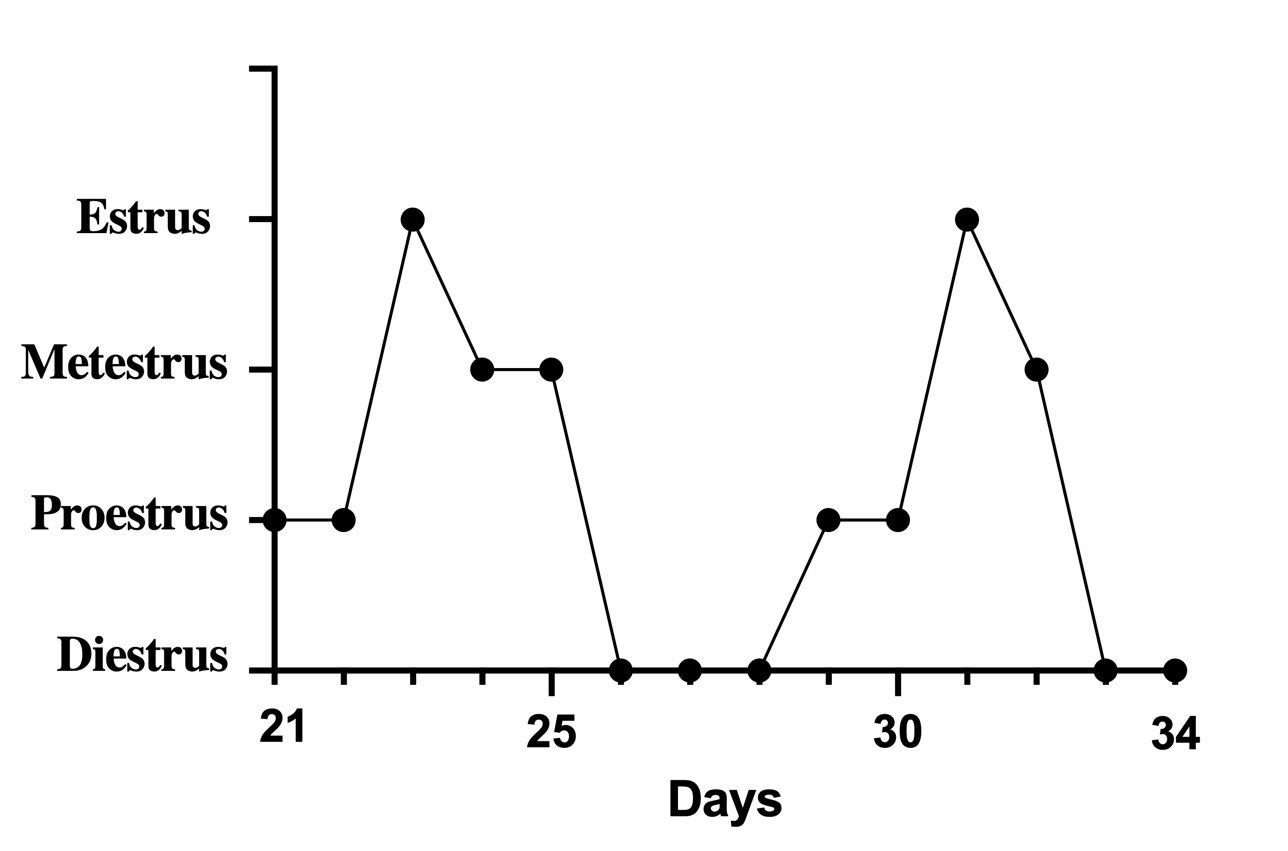 | 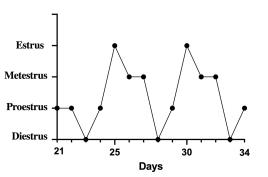 | 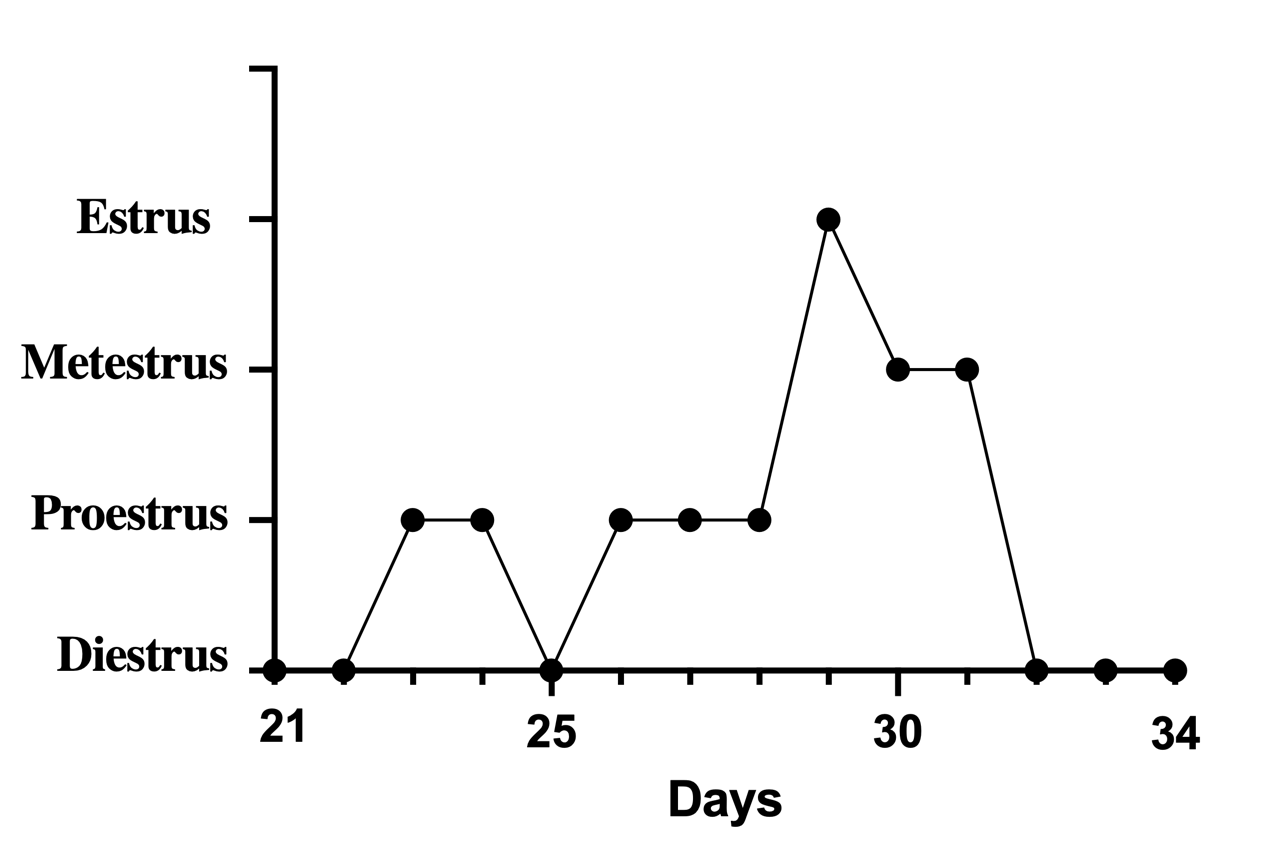 |
| 11 | 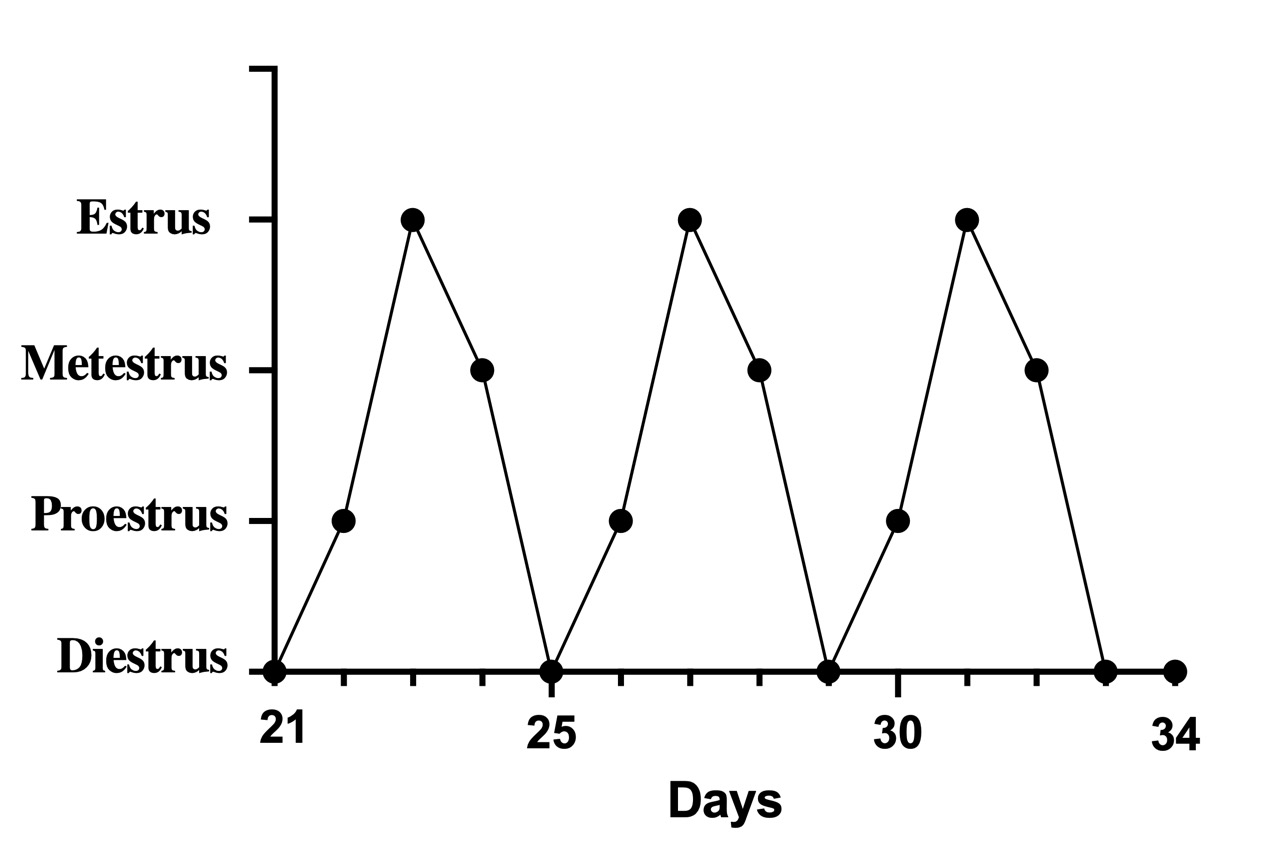 | 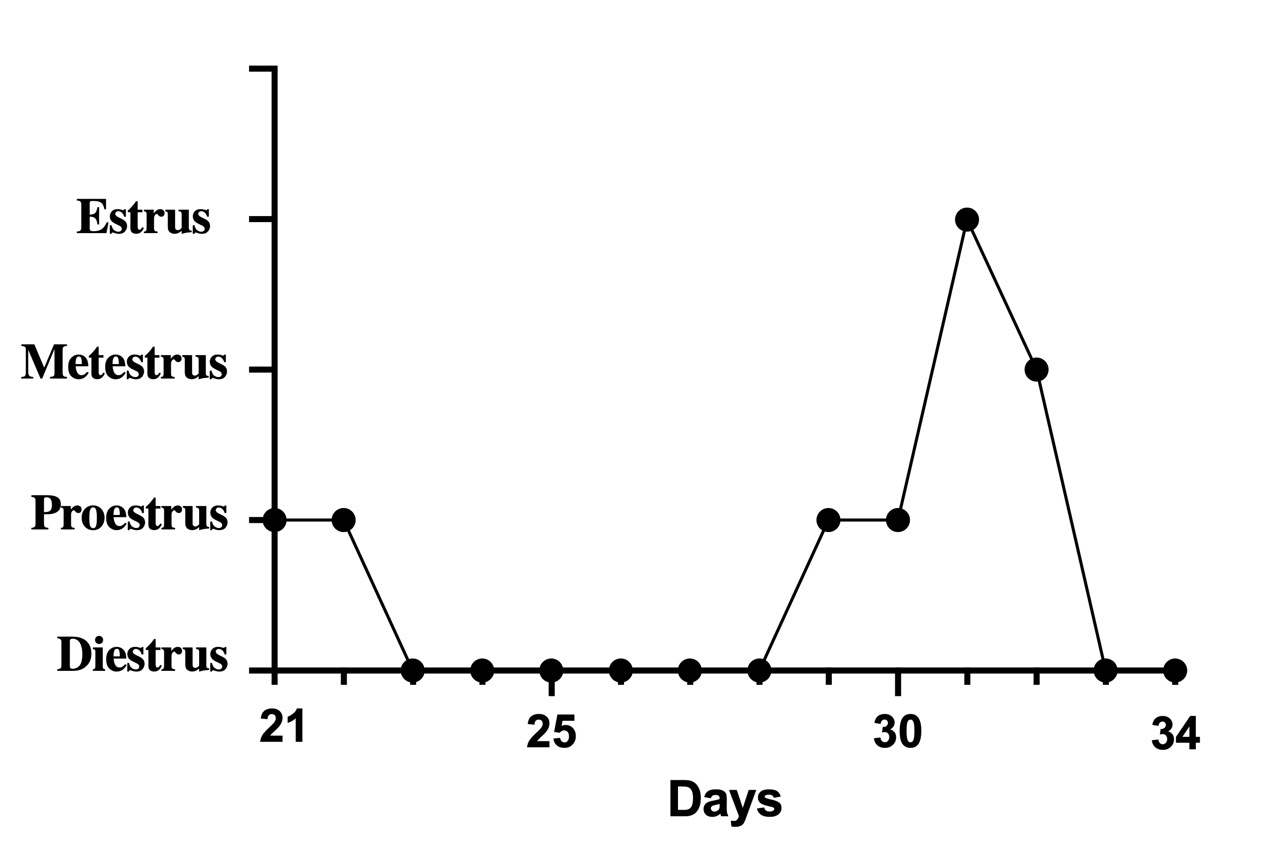 | 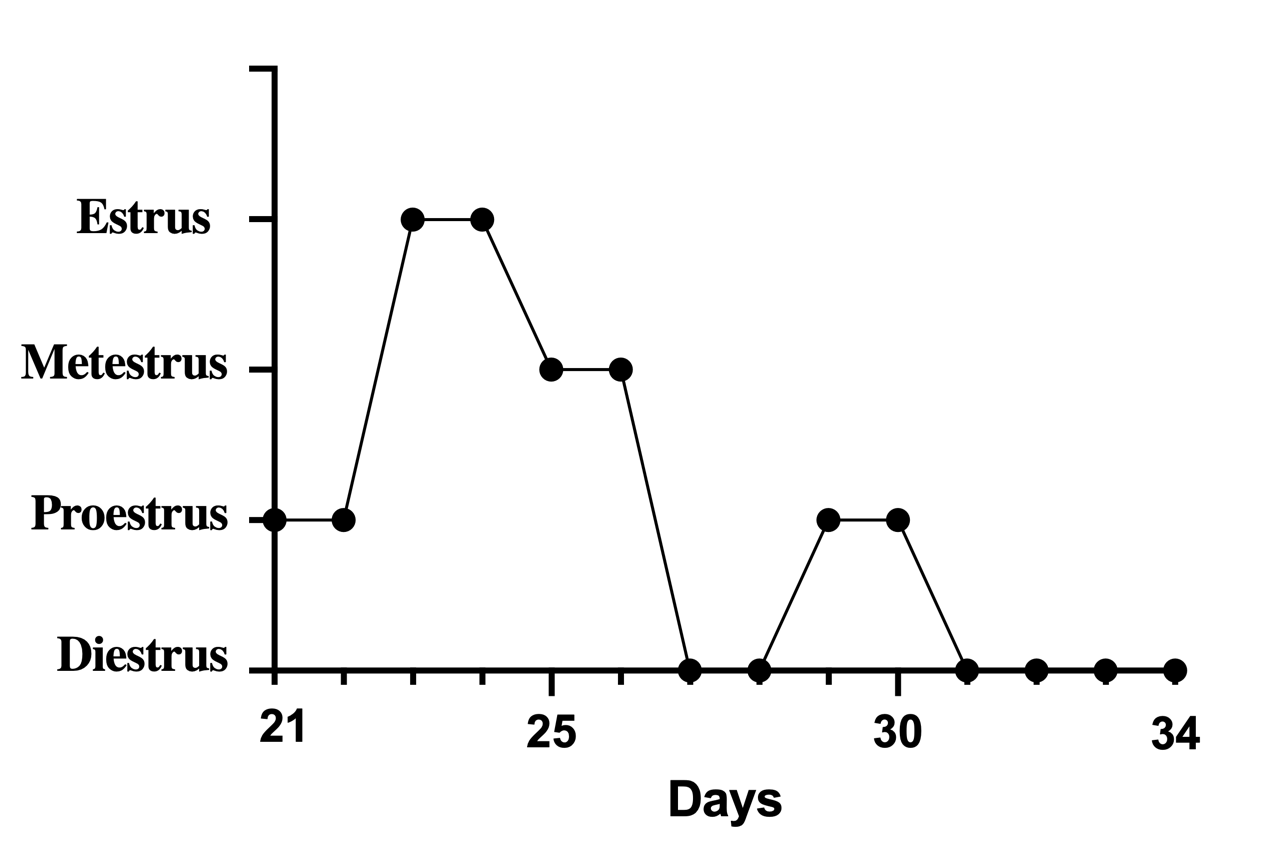 | 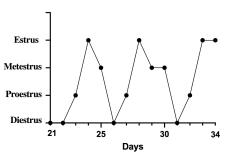 |
| 12 | 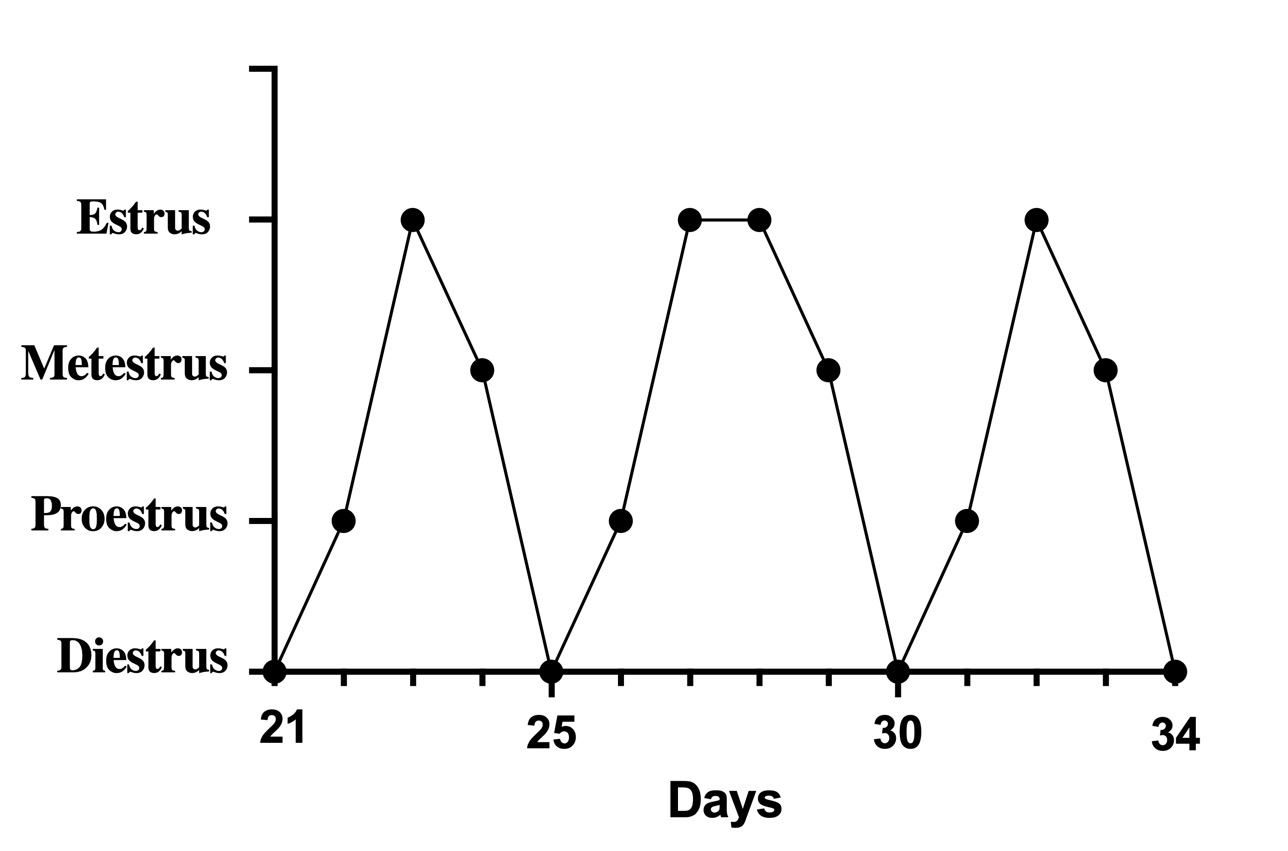 | 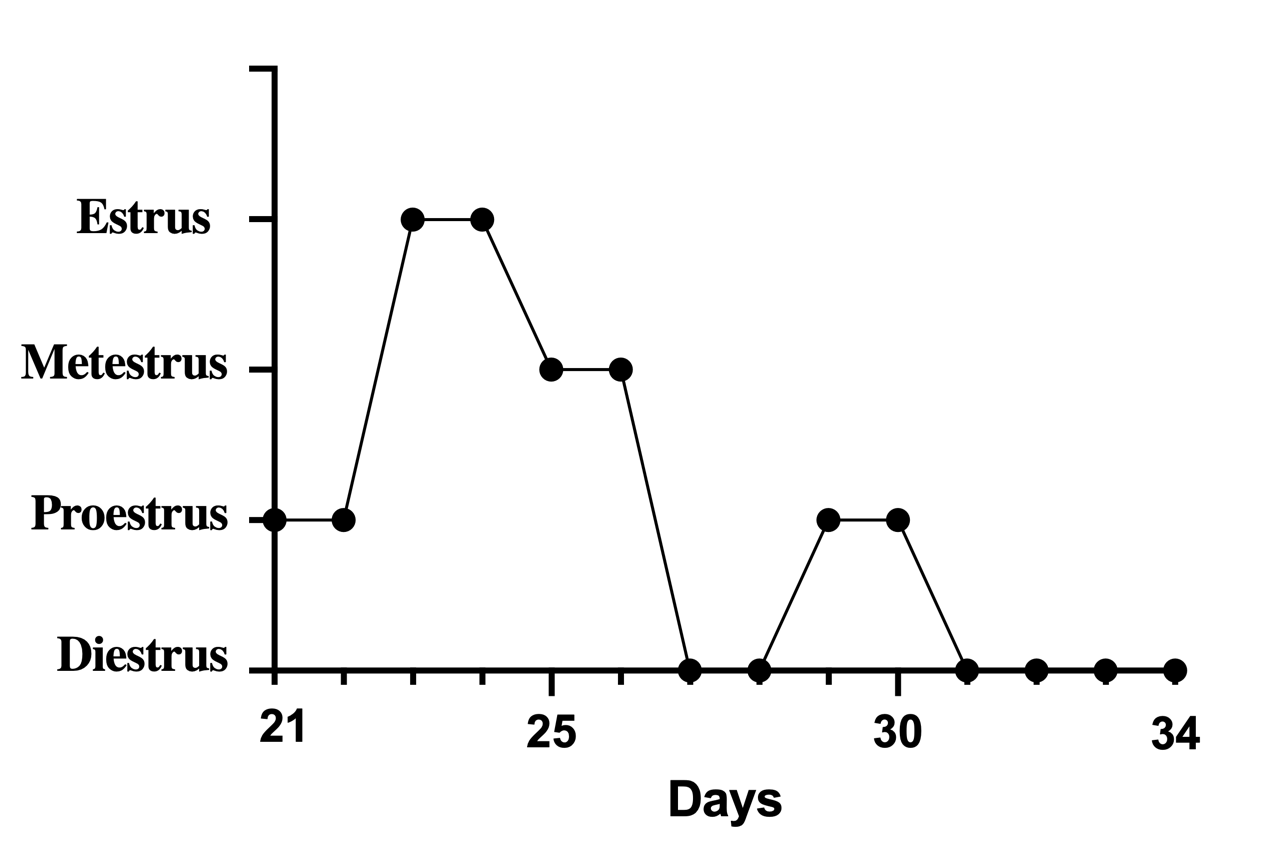 | 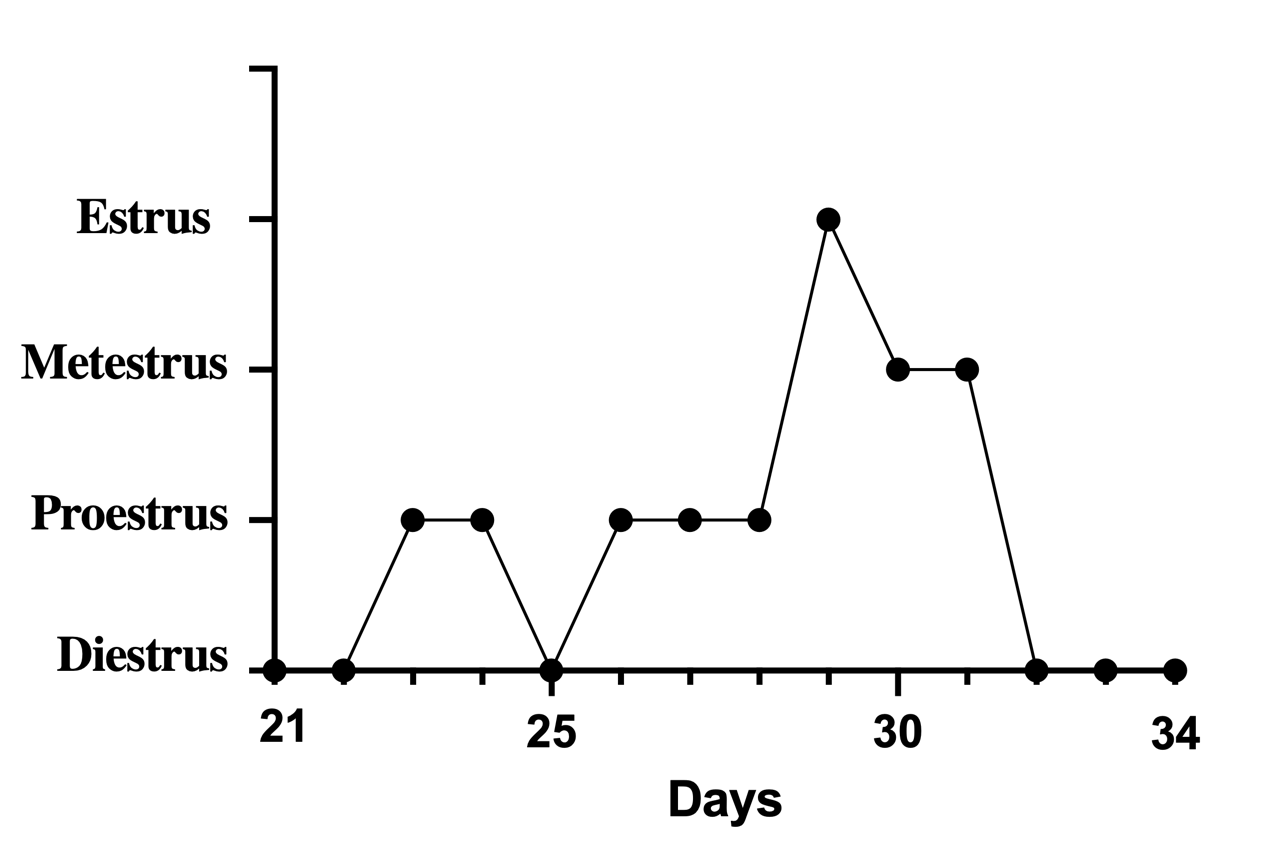 | 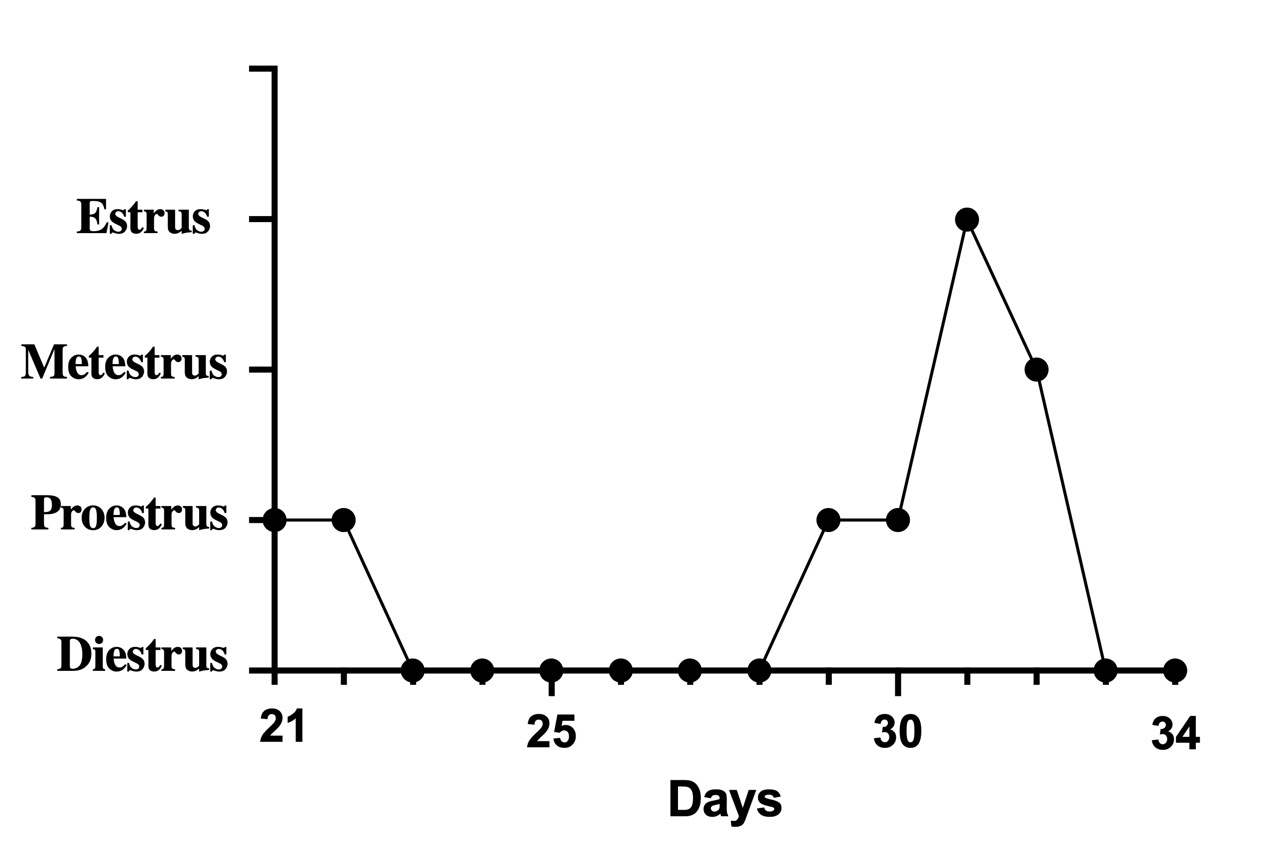 |
| 13 | 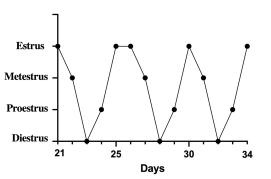 | 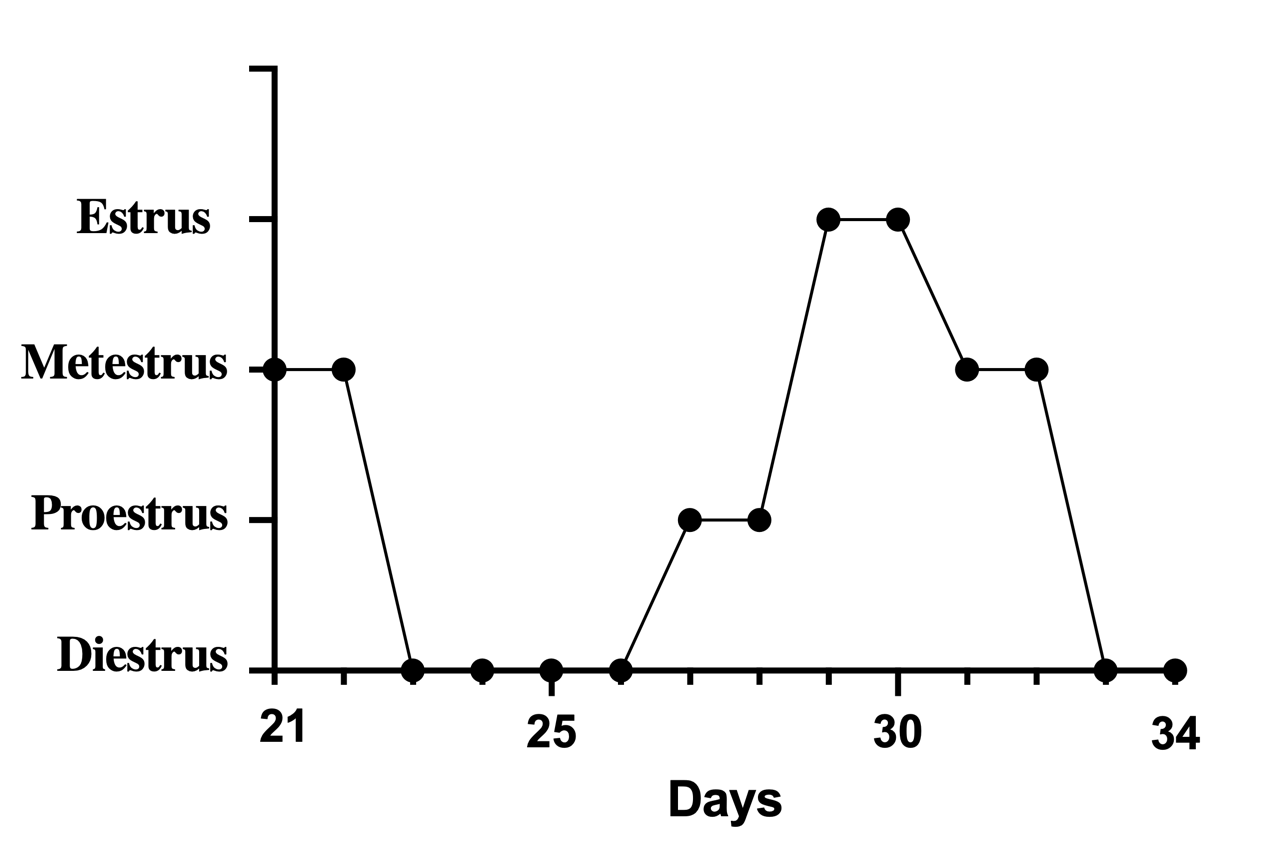 | 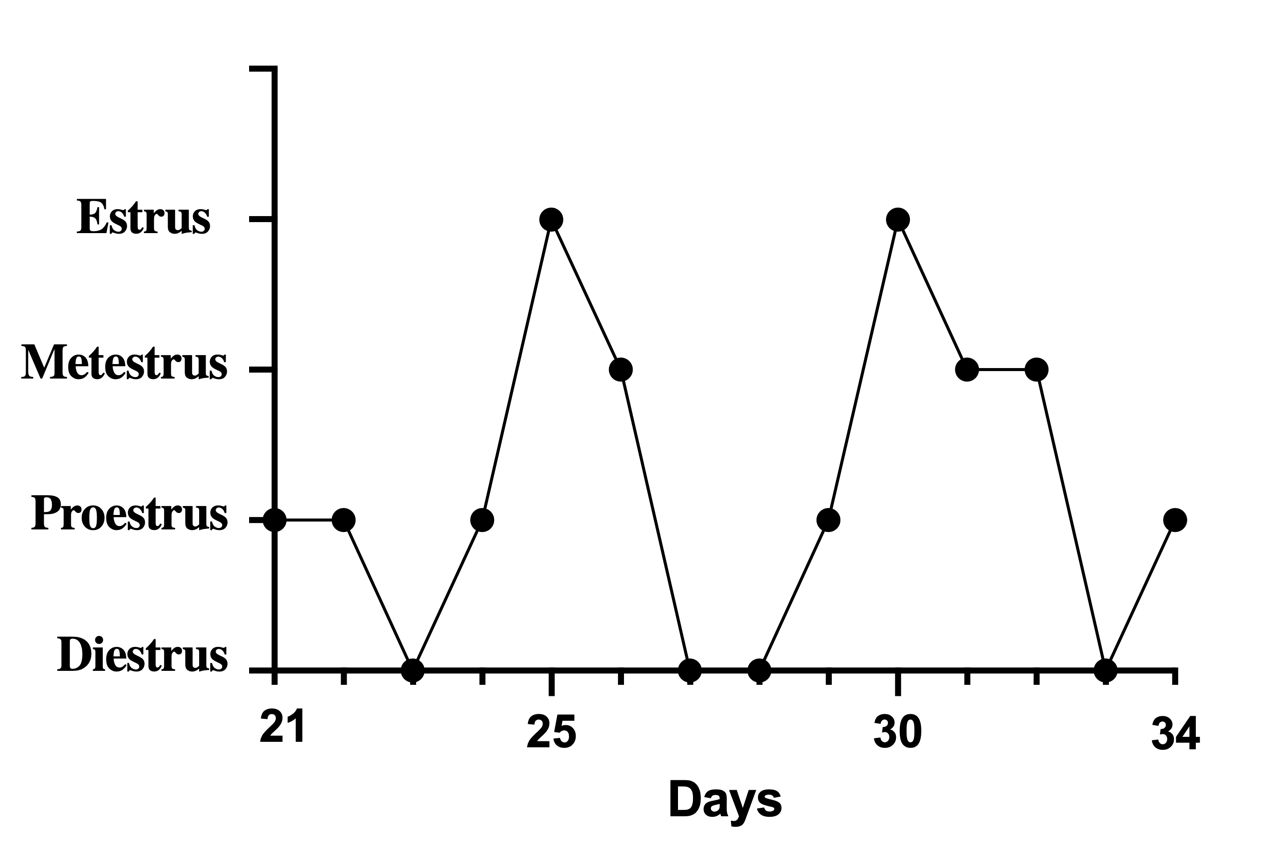 | 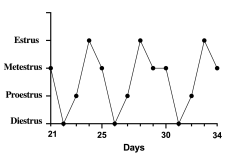 |
| 14 | 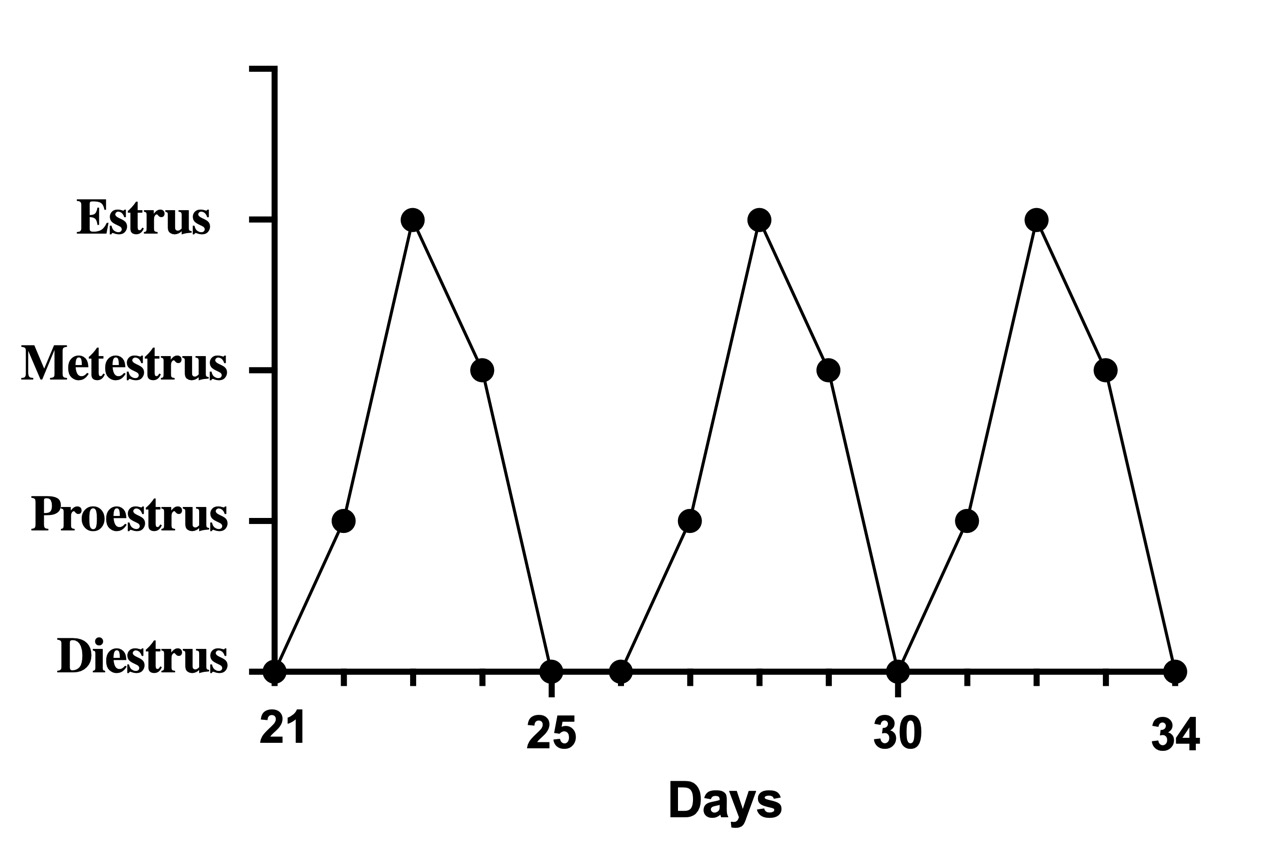 | 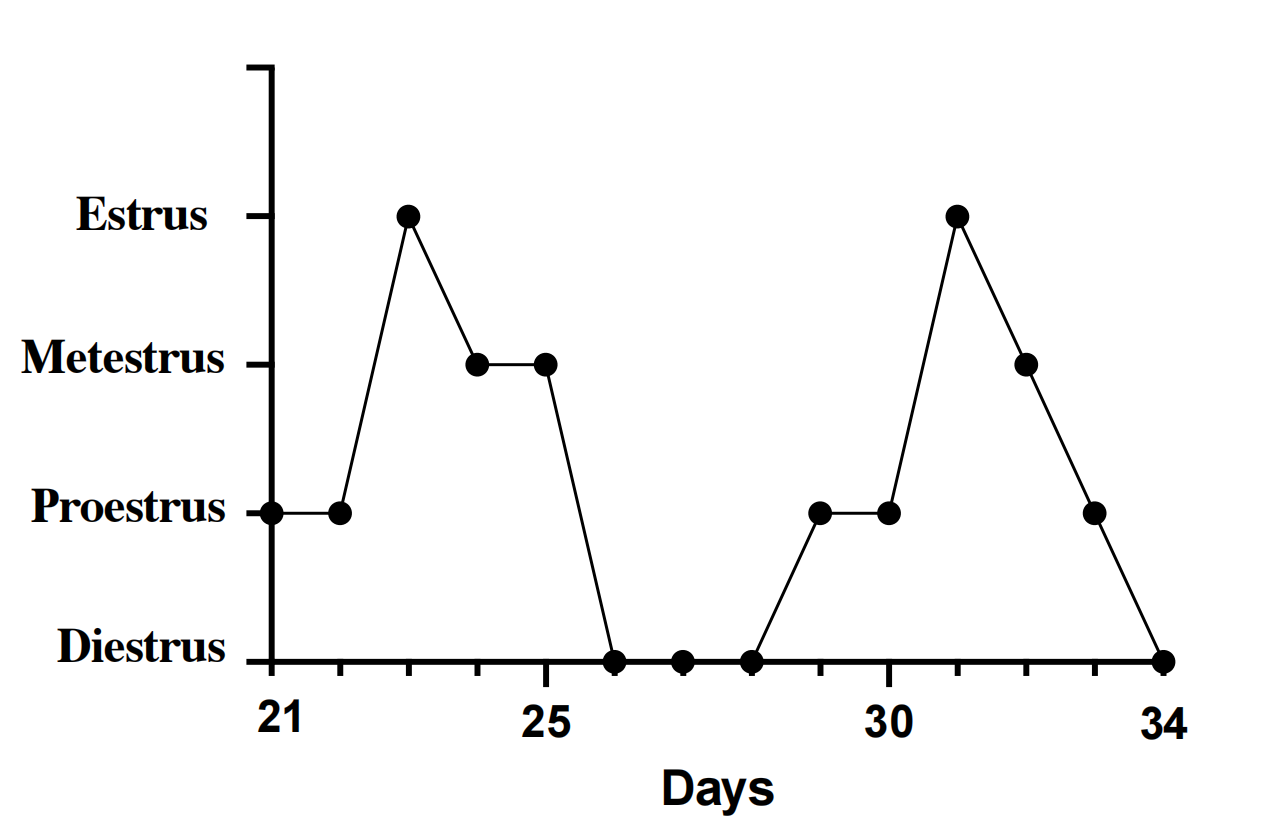 | 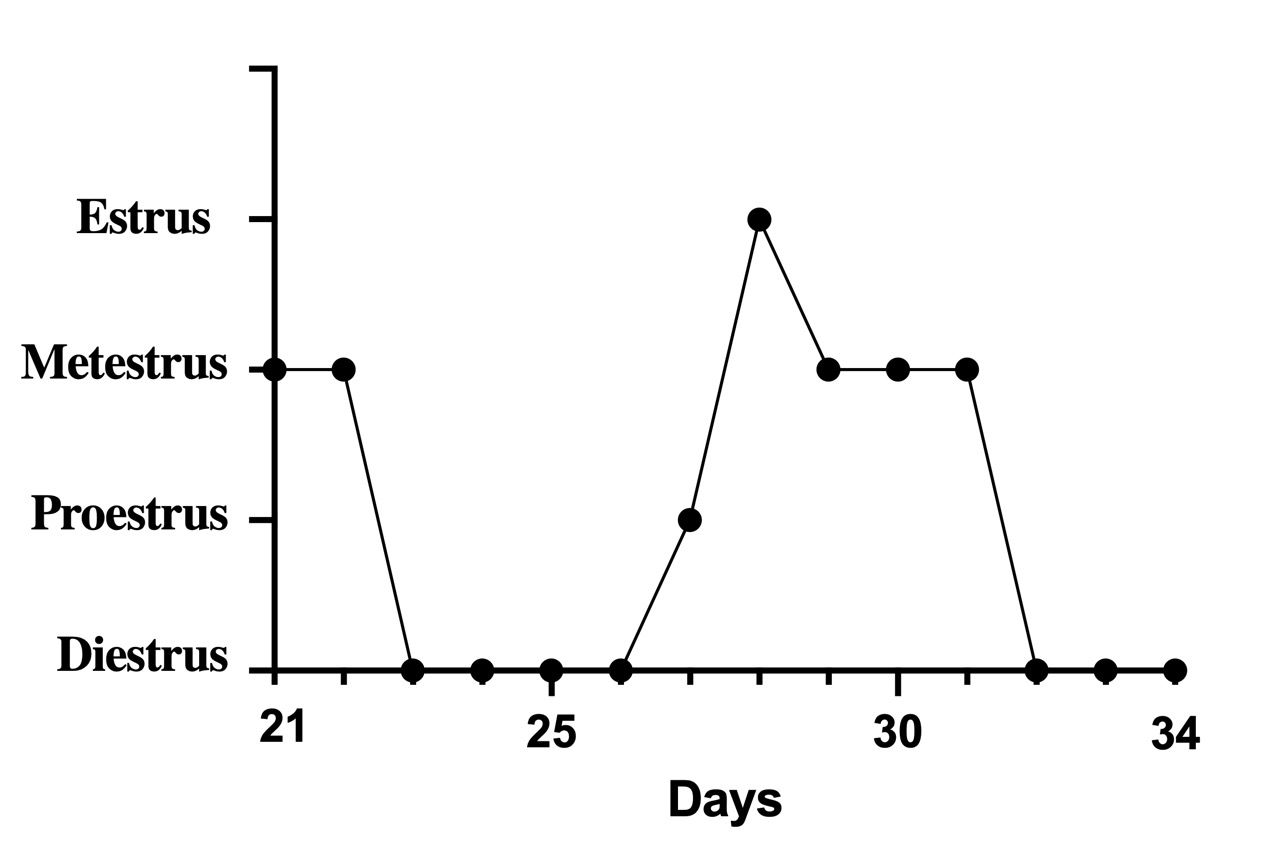 | 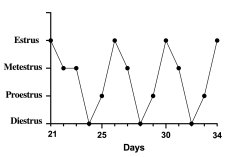 |
| 15 | 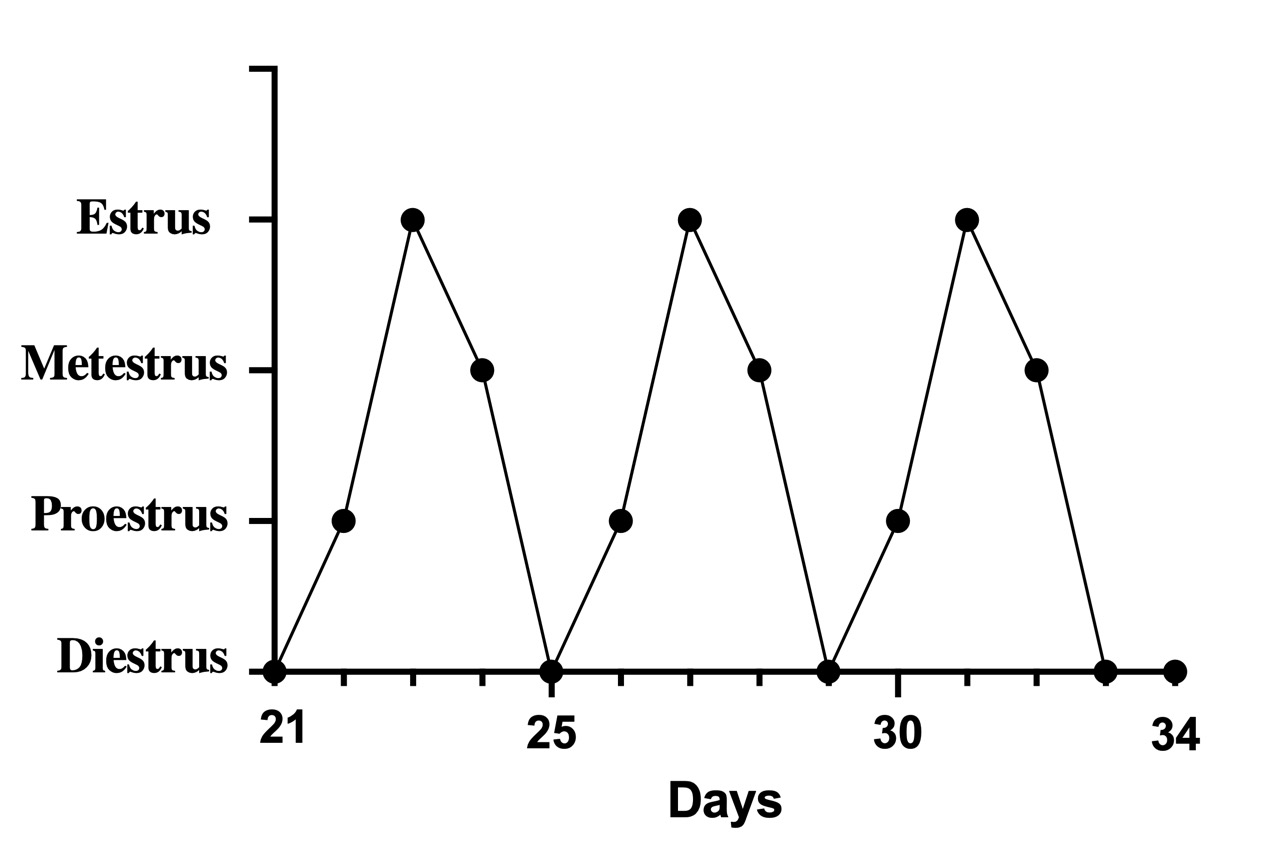 | 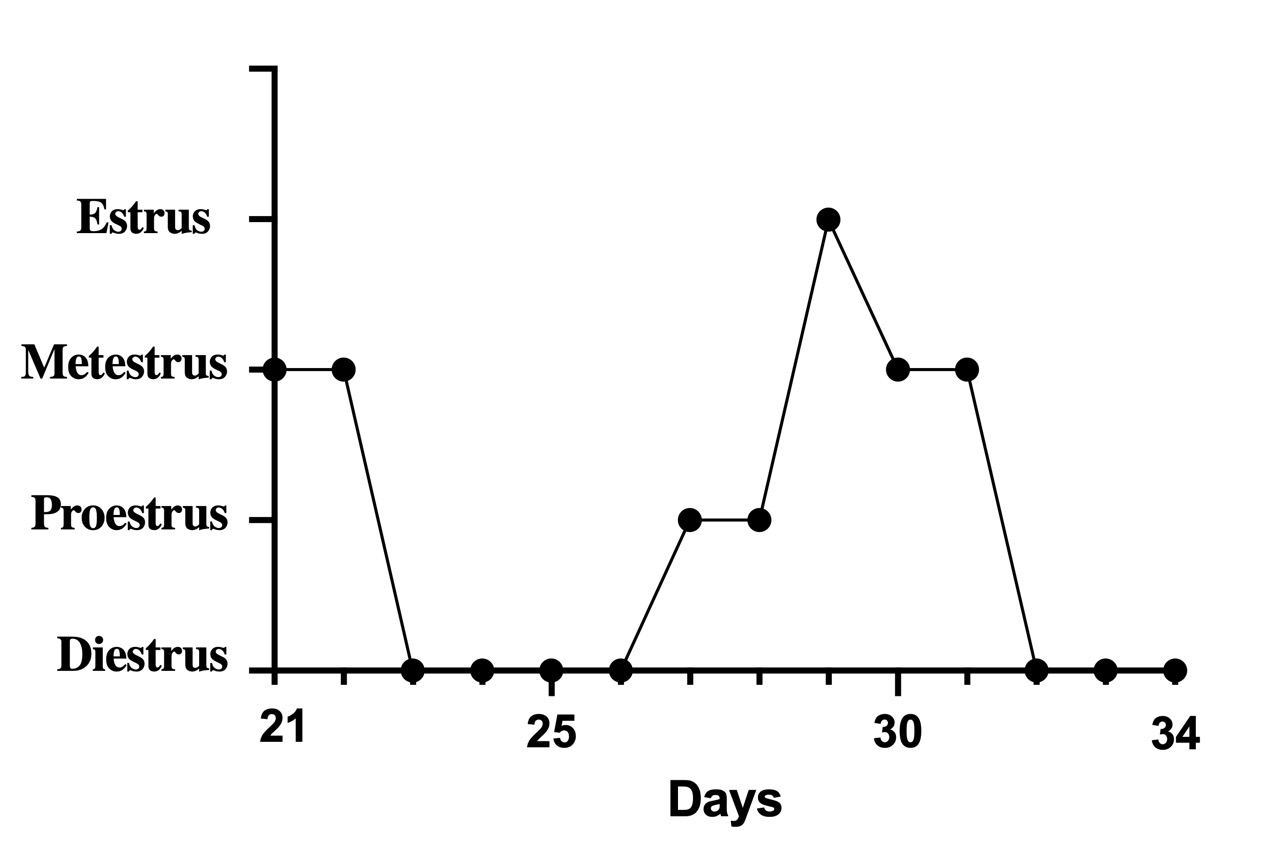 | 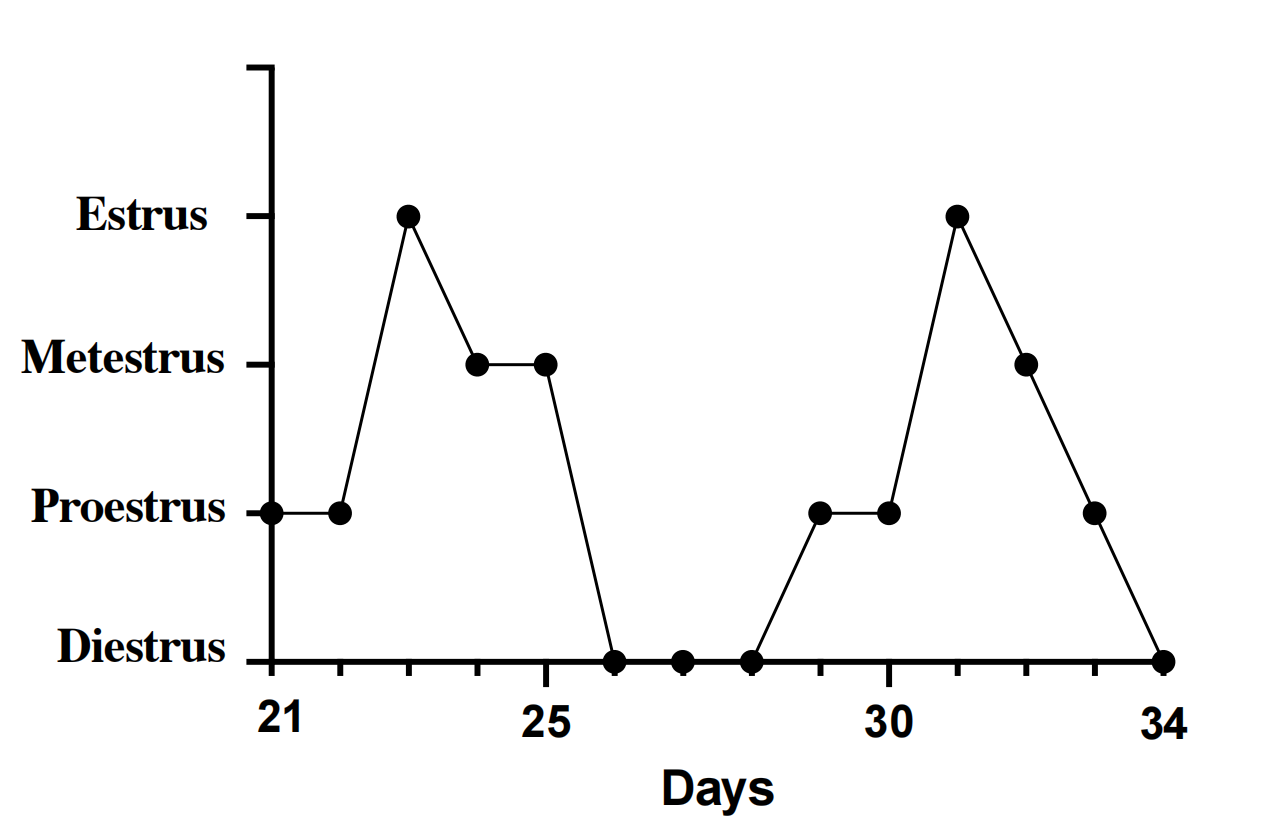 | 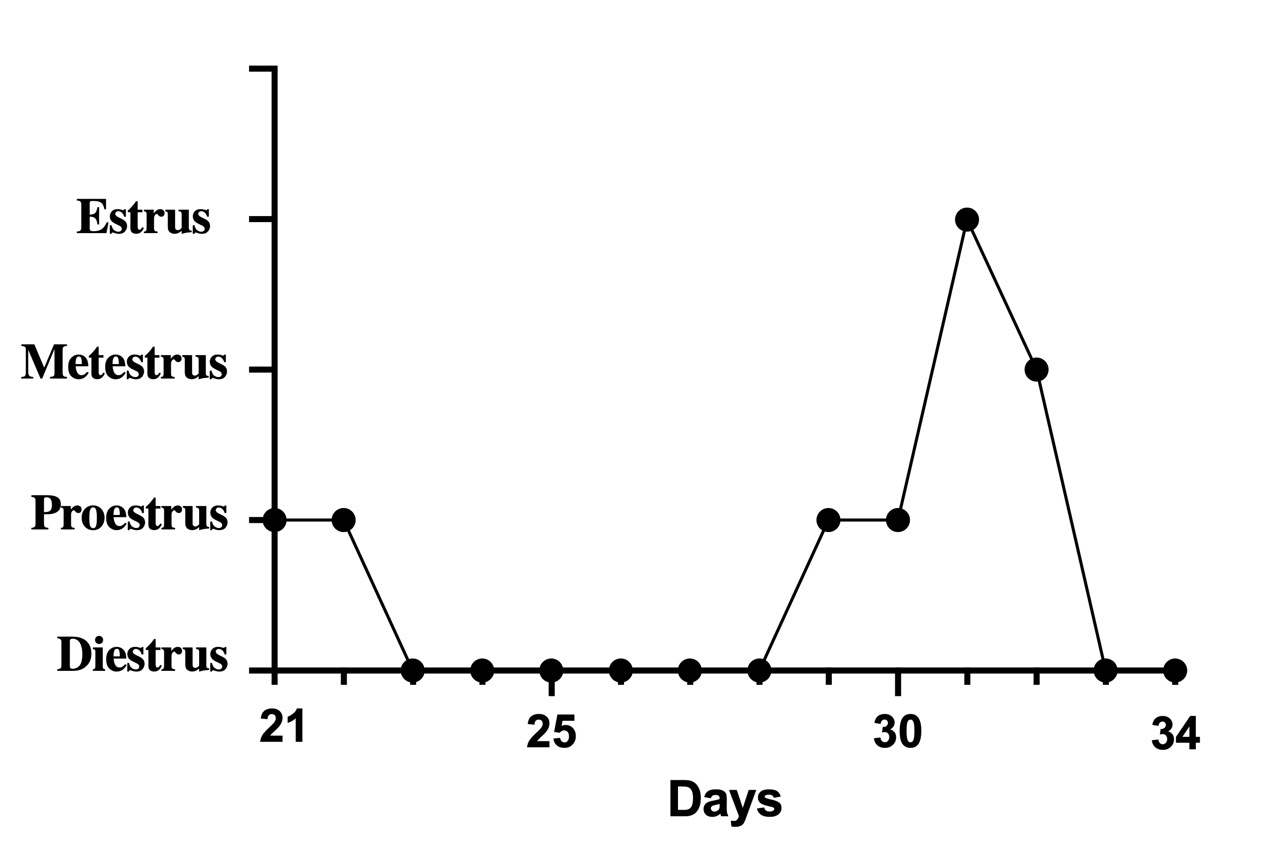 |

Supplement: Supplementary file 2 [file Supplementaryfile1.docx]

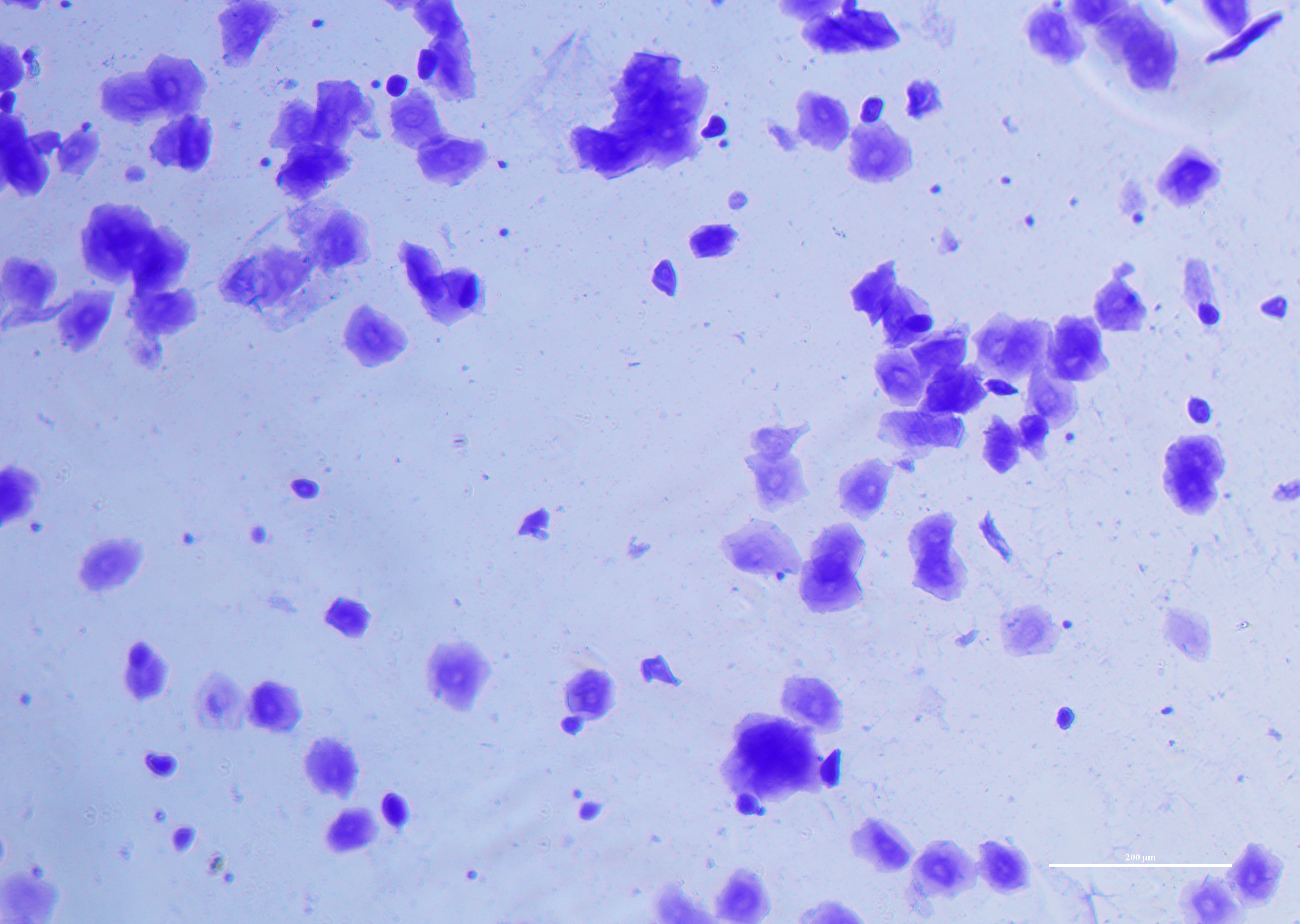

Supplement: Supplementary file 3 [file Image1.tif]

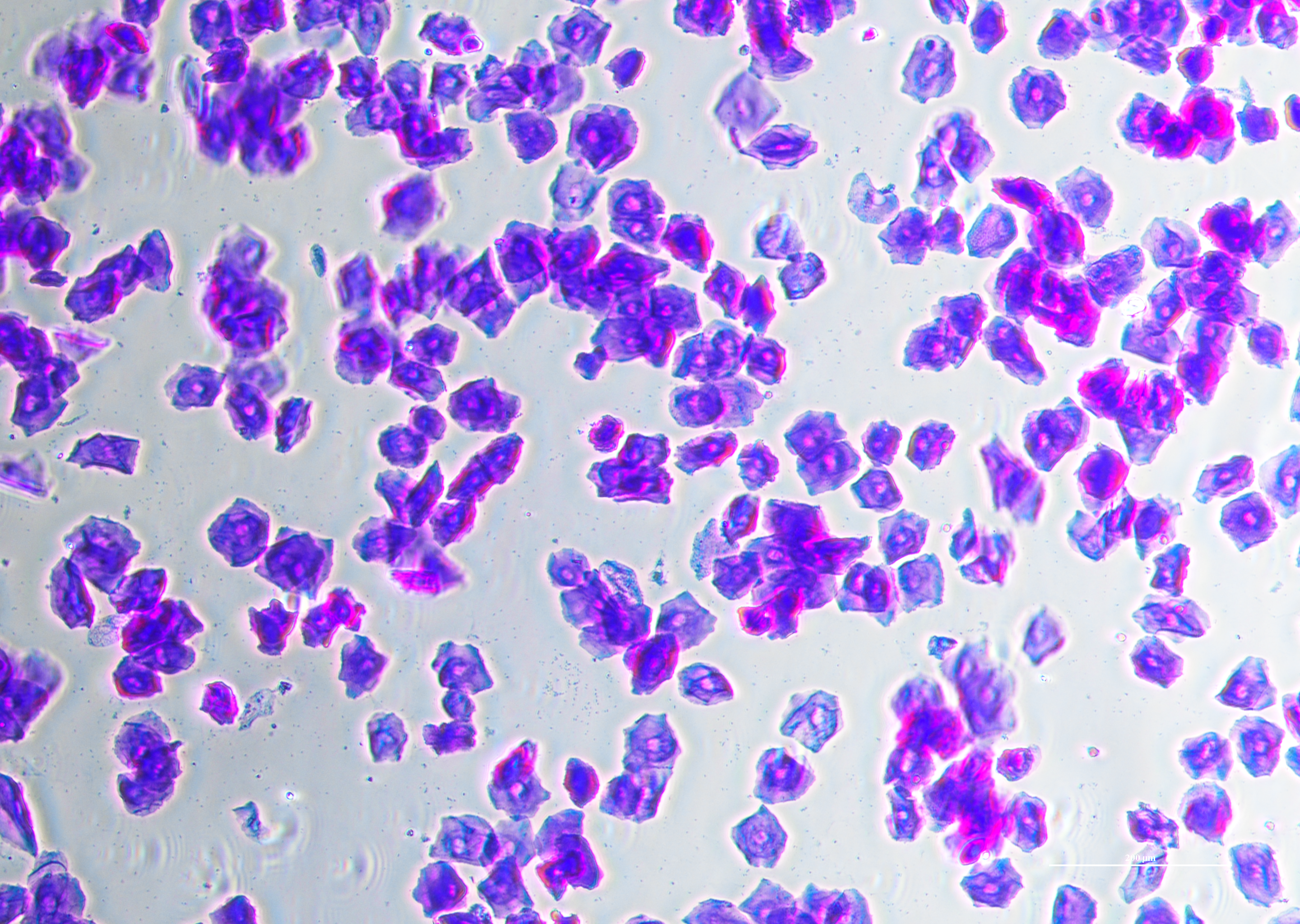

Supplement: Supplementary file 4 [file Image2.tif]

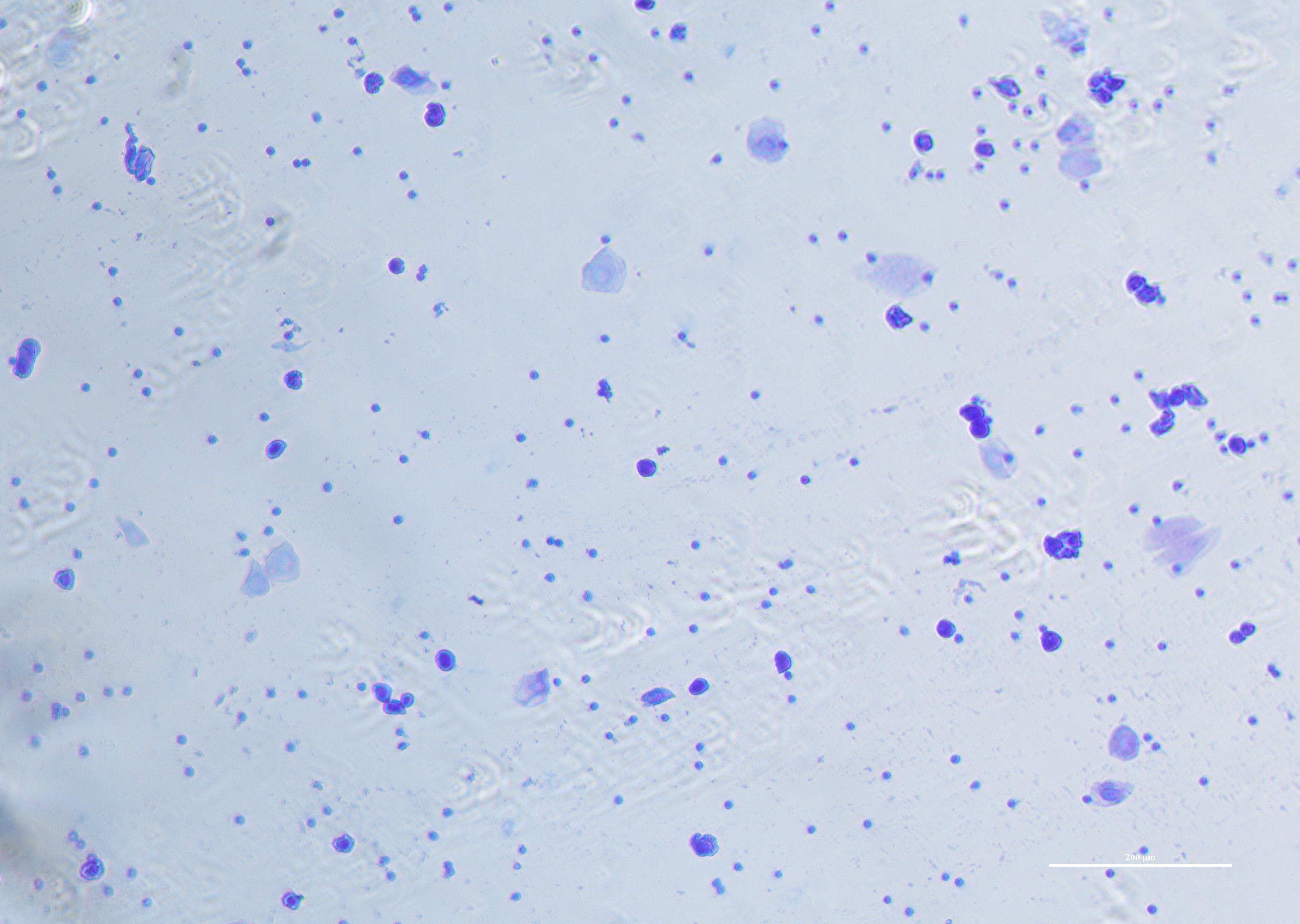

Supplement: Supplementary file 5 [file Image3.tif]

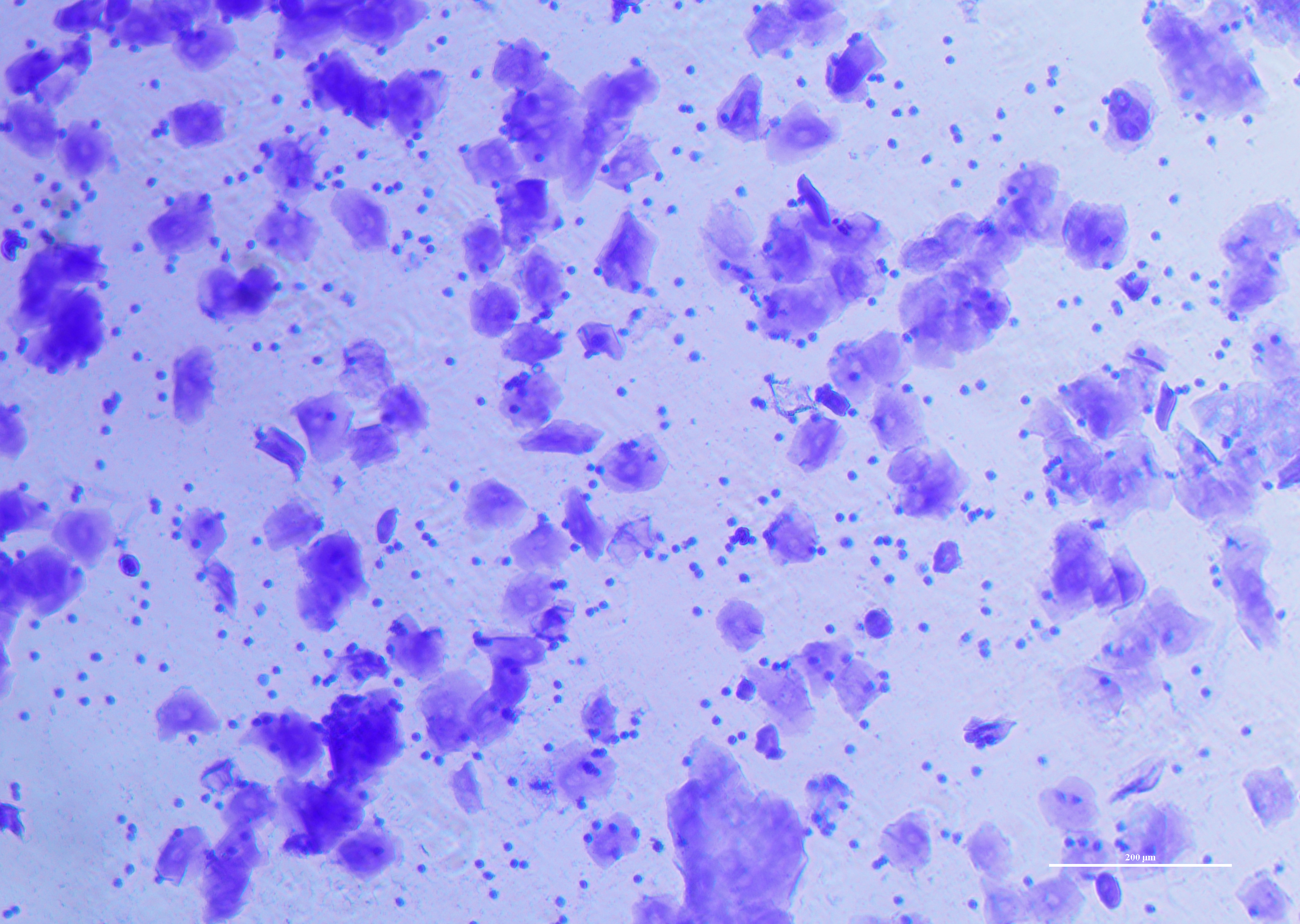

Supplement: Supplementary file 6 [file Image4.tif]
